# CEMiTool: a Bioconductor package for performing comprehensive modular co-expression analyses

**DOI:** 10.1186/s12859-018-2053-1

**Published:** 2018-02-20

**Authors:** Pedro S. T. Russo, Gustavo R. Ferreira, Lucas E. Cardozo, Matheus C. Bürger, Raul Arias-Carrasco, Sandra R. Maruyama, Thiago D. C. Hirata, Diógenes S. Lima, Fernando M. Passos, Kiyoshi F. Fukutani, Melissa Lever, João S. Silva, Vinicius Maracaja-Coutinho, Helder I. Nakaya

**Affiliations:** 1 Department of Clinical and Toxicological Analyses, School of Pharmaceutical Sciences, University of São Paulo, São Paulo, SP 05508-900 Brazil; 2 Advanced Center for Chronic Diseases (ACCDiS), Facultad de Ciencias Químicas y Farmacéuticas, Universidad de Chile, Santiago, Chile; 3 Department of Biochemistry, Immunology, and Cell Biology, University of São Paulo, Ribeirão Preto, São Paulo Brazil

**Keywords:** Co-expression modules, Gene networks, Modular analysis, Leishmaniasis, Transcriptomics

## Abstract

**Background:**

The analysis of modular gene co-expression networks is a well-established method commonly used for discovering the systems-level functionality of genes. In addition, these studies provide a basis for the discovery of clinically relevant molecular pathways underlying different diseases and conditions.

**Results:**

In this paper, we present a fast and easy-to-use Bioconductor package named CEMiTool that unifies the discovery and the analysis of co-expression modules. Using the same real datasets, we demonstrate that CEMiTool outperforms existing tools, and provides unique results in a user-friendly html report with high quality graphs. Among its features, our tool evaluates whether modules contain genes that are over-represented by specific pathways or that are altered in a specific sample group, as well as it integrates transcriptomic data with interactome information, identifying the potential hubs on each network. We successfully applied CEMiTool to over 1000 transcriptome datasets, and to a new RNA-seq dataset of patients infected with *Leishmania*, revealing novel insights of the disease’s physiopathology.

**Conclusion:**

The CEMiTool R package provides users with an easy-to-use method to automatically implement gene co-expression network analyses, obtain key information about the discovered gene modules using additional downstream analyses and retrieve publication-ready results via a high-quality interactive report.

**Electronic supplementary material:**

The online version of this article (10.1186/s12859-018-2053-1) contains supplementary material, which is available to authorized users.

## Background

Cellular processes are controlled by a host of interacting molecules whose activity and levels are frequently co-regulated or co-expressed. Detecting the groups (i.e. modules) of co-expressed genes in a myriad of biological conditions has generated important insights in brain evolution [1], coronary artery disease [2], and macrophage activation [3], among many other biological conditions.

Following evidence that genes interact with each other in a scale-free fashion [4], Zhang and Horvath developed an R package named WGCNA (Weighted Gene-Coexpression Network Analysis) that identifies co-expressed gene modules [5]. Although tutorials and examples are available for using the package, following its workflow verbatim is time-consuming and tiresome. Moreover, users are often required to manually select parameters and to filter the input genes prior running WGCNA. This hinders workflow automation and can impact reproducibility since different researchers may utilize different parameters, obtaining distinct results for the same data set. More importantly, WGCNA is limited in terms of the functional analyses available for the package users.

After identifying co-expressed gene modules, researchers are often interested in performing functional and integrative analyses. Over-representation analysis (ORA) can be used to reveal if a set of co-expressed genes is enriched for genes belonging to known pathways or functions. In addition, gene set enrichment analysis (GSEA) [6] can associate the activity of a module with the study phenotypes (i.e. sample group). Finally, integrating co-expression information with protein-protein interaction data can be useful to identify main regulators or hubs. Such analyses, however, require the combination of several packages and programs, and considerable bioinformatics skills.

To address these issues, we developed the Co-Expression Modules identification Tool (CEMiTool), an R package that allows users to easily identify and analyze co-expression modules in a fully automated manner. CEMiTool provides users with a novel unsupervised gene filtering method, automated parameter selection for identifying modules, enrichment and module functional analyses, as well as integration with interactome data. Our tool then reports everything in HTML web pages with high-quality plots and interactive tables.

Using the same real datasets, we compared the features of CEMiTool against existing tools, and showed that our tool outperforms them in several aspects. We also applied CEMiTool to over 1000 microarrays and RNA-seq datasets, demonstrating its power in automating the generation of co-expression gene modules and subsequent analyses. Finally, to gain a better insight of the pathophysiology of Leishmania infection, we ran CEMiTool on a novel RNA-seq dataset, which was generated from the blood of infected patients. Our analyses revealed that several modules contained genes not previously associated with Leishmaniasis. The R package is freely available in Bioconductor (DOI: 10.18129/B9.bioc.CEMiTool), and as a Docker image file as well (https://hub.docker.com/r/csblusp/cemitool).

## Implementation

**Fig. 1:**
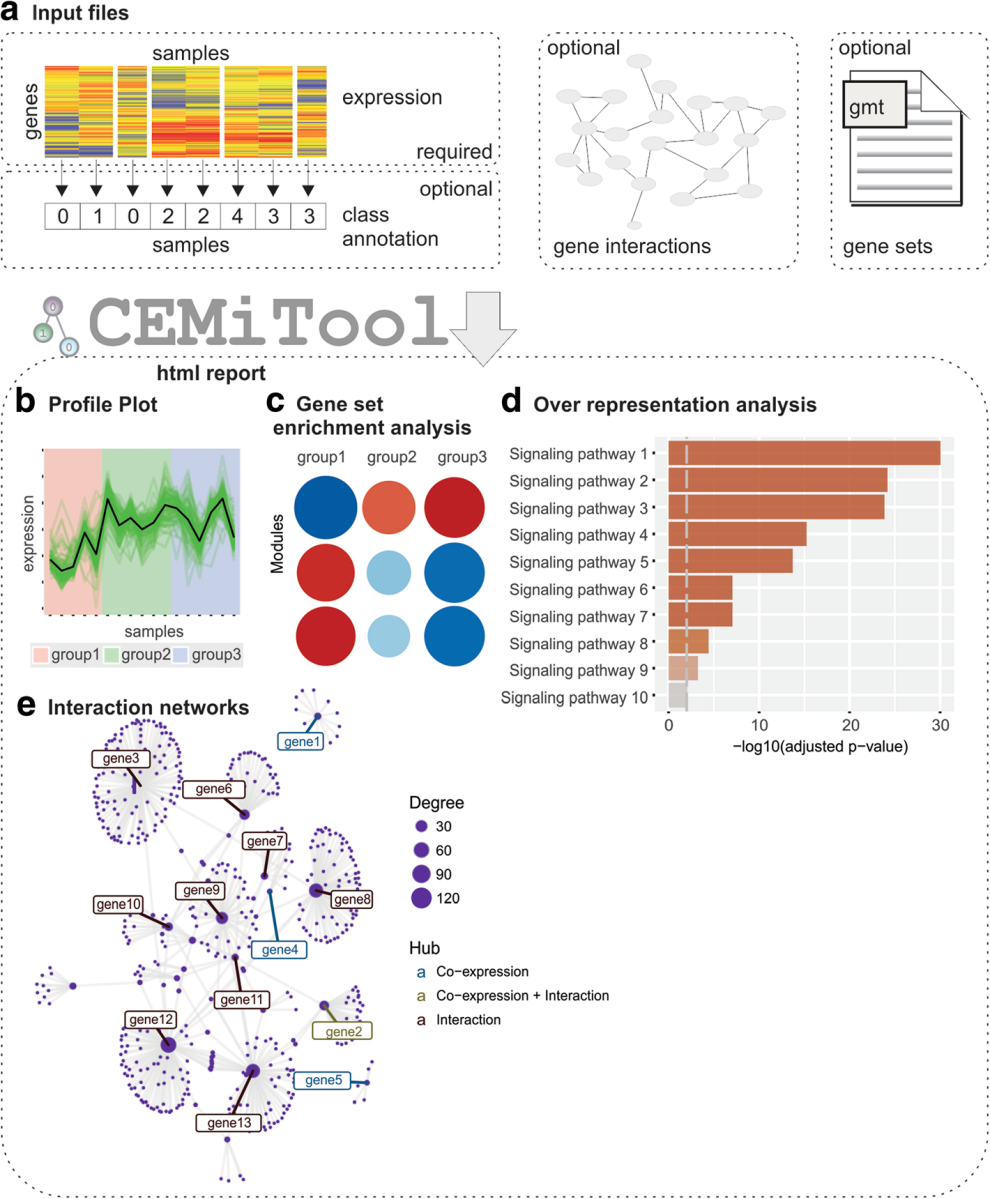
Overview of CEMiTool. **a** CEMiTool requires a gene expression file to identify the modules and optional files to: (**b**) visualize the expression profile of individual genes across samples from different groups, which are defined by the user and shown as different colors; (**c**) perform Gene Set Enrichment Analyses, showing the module activity on each group of samples; (**d**) run over representation analysis to define module functions; and (**e**) create gene networks, displaying the top ten most connected genes (hubs)

CEMiTool is an easy-to-use package, automating within a single R function (*cemitool*) the entire module discovery process - including gene filtering and functional analyses (Fig. 1). The process begins with a gene expression file containing the genes as rows and the samples as columns. This file is the only required input for CEMiTool’s analyses. An unsupervised filtering method based on the inverse gamma distribution (Additional file 1: Text) will then select the genes used in the analyses. Next, a soft-thresholding power β [5] is chosen using our modified algorithm (Additional file 1: Text), and this value is used to determine a similarity criterion between pairs of genes. The genes are then separated into modules using the Dynamic Tree Cut package [5, 7]. If an optional file containing gene interactions (e.g. protein-protein interaction data) is provided, the package will return network graphs composed of interacting genes within the same module. Additionally, if the user provides a sample annotation file, CEMiTool can perform gene set enrichment analysis (GSEA), allowing users to visualize which modules are induced or repressed in the different phenotypes. Finally, given an optional file containing gene sets, CEMiTool will perform an over representation analysis (ORA) based on the hypergeometric test to determine the most significant module functions.

### Over representation analysis of modules

To determine the biological functions possibly related to each module, CEMiTool is able to take a user-provided gene pathway list and perform an over representation analysis (ORA) via the clusterProfiler R package [8]. CEMiTool will then report the adjusted *p*-value negative logarithm for the top gene sets enriched on each co-expression module based on the hypergeometric test. This analysis is also available in the WGCNA package via the *userListEnrichment* function, however its output is in tabular form, while CEMiTool returns both a table and a bar graph of the most significantly enriched pathways for each module.

### Association of module activity to sample phenotypes

If the user submits a sample annotation file describing the phenotypes (i.e. disease, healthy, treated, etc) of samples, CEMiTool performs a gene set enrichment analysis using the fgsea (Fast Gene Set Enrichment Analysis) R package [9]. In this analysis, genes from co-expression modules will be treated as gene sets and the z-score normalized expression of the samples within each phenotype will be treated as rankings on the analysis. The results will assess if the activity of a module is altered across different phenotypes.

### Adding gene interactions to modules

Users can also provide a gene interaction file to visualize the interactions between the genes in each co-expression module. This allows users to customize their module graphs according to different interaction databases. The top ten network hubs (genes with the highest connectivities) are highlighted in the graph. The resulting network is provided as a graph (one per module) in the HTML report.

We compared the features provided by CEMiTool with existing tools for co-expression module identification and analysis, namely WGCNA, Petal [10], CoP [11], GeNET [12], DiffCoEx [13], CoXpress [14], DICER [15] and DINGO [16], as shown in Table 1. However, none of the tools evaluated have all the features provided by CEMiTool.

**Table 1:** Features Provided by Programs that Identify Co-Expression Modules. Over representation analysis of CoP, and GeNET programs is considered “limited” because they only allow the usage of specific gene sets (GO, Pfam or KEGG). The runtime of 2 studies using the same computer and default settings are shown for CEMiTool, WGCNA, and Petal

Features	CEMiTool	WGCNA	Petal	CoP	GeNET	DiffCoEx	CoXpress	DICER	DINGO
Automatic Gene Filtering	yes	no	no	no	no	no	no	no	no
Over representation analysis	yes	yes	no	limited	limited	no	no	no	no
Gene set enrichment analysis	yes	no	no	no	yes	no	no	no	no
Integration with interactome	yes	no	no	no	no	no	no	no	no
Report in HTML	yes	no	no	yes	no	no	no	no	no
Native plots	yes	yes	no	no	yes	no	no	no	no
Search for a gene or gene list	yes	no	yes	yes	yes	no	no	no	no
Merging modules	yes	yes	no	no	no	yes	no	yes	no
Allows 2+ sample groups	yes	yes	yes	yes	yes	no	no	no	no
R package	yes	yes	yes	no	no	no	yes	no	yes
Year of last update	2017	2017	2017	2010	Unknown	Unknown	2013	Unknown	Unknown
Runtime for study GSE18090	2min12s	3min10s	17min18s	–	–	–	–	–	–
Runtime for study GSE43777	3min03s	4min33s	40min10s	–	–	–	–	–	–

## Results and discussion

### Co-expressed gene module selection and benchmark

We utilized two publicly available microarray studies of Dengue infection (GSE18090 and GSE43777) to compare CEMiTool to two R packages: WGCNA and Petal [10]. CEMiTool was run using its default parameters and all optional files. After filtering, the analyses were performed on 2129 genes for study GSE18090, and 1765 genes for study GSE43777. Our assumption is that greater gene set enrichment in pathways relevant to the diseases are good proxies for the quality of a co-expression network analysis. For study GSE18090, CEMiTool selected a soft-threshold value of 6 and identified 12 different co-expression modules, out of which 9 had at least one significantly enriched pathway in the Over Representation Analysis. Notably, modules M4 and M6 were significantly enriched with interferon and cytokine signaling pathways, along with antiviral mechanisms, as expected from an infectious disease such as dengue. Furthermore, module M2 was significantly enriched for toll-like receptor cascades, which have been shown to lead to and induce the release of proinflammatory cytokines and chemokines in Dengue infections. These findings mirror what was found in the 7 significantly enriched (of a total of 11) co-expression modules observed for study GSE43777 (beta = 5). Running CEMiTool analyses with all possible optional files for both studies in an average computer took around 3 min (Table 1).

In order to compare WGCNA to CEMiTool, WGCNA was run on the Dengue studies using the top 4000 most variant genes of each dataset. Since WGCNA does not specify the optimal number of input genes, we utilized the same number of genes suggested in their tutorial. The analysis identified 18 modules for study GSE18090 using a soft-threshold of 9. Interestingly, however, over half of them (10) presented no significantly enriched pathways after Over Representation Analysis (*p*-value < 0.01). In contrast to the CEMiTool results, WGCNA did not report pathways related to toll-like receptor cascades. As for study GSE43777 (beta = 6), WGCNA returned 10 significantly enriched modules out of a total of 16. These results suggest that, despite running on a smaller number of genes, CEMiTool is able to successfully filter irrelevant genes and construct modules using the most important genes. Our custom WGCNA script was able to run the analysis in a similar time as CEMiTool (around 3 min, Table 1). However, this did not take into account the considerable time required to manually insert all steps needed to perform WGCNA analyses, select the user-specified parameters, and the steep learning curve necessary in order to understand the whole procedure.

To account for the difference in the number of input genes, we also ran WGCNA using the filtered datasets returned by CEMiTool’s filter. For study GSE18090, WGCNA identified 16 modules, with a soft-threshold of 7. Out of these, 10 modules had at least one significantly enriched pathway in the Over Representation Analysis. As expected, results became more similar to CEMiTool’s, with the inclusion of a module related to Toll-like receptor activity (M2), and different modules for interferon types gamma (M4) and alpha/beta (M5). As for study GSE43777, WGCNA (beta value of 6) was able to identify 6 significantly enriched modules out of a total of 12, giving it 2 more non-significantly enriched modules than CEMiTool. These subtle differences are likely to be derived from the difference in the selected beta values and showcase CEMiTool’s ability to produce results comparable to established tools such as WGCNA with greater ease and convenience.

We ran Petal using the same input genes utilized by WGCNA analysis (4000 most variant genes). Petal is a software which attempts to define a co-expression network using an automatically defined threshold to indicate similar expression between genes [10]. However, after 20 min for study GSE18090 and 40 min for study GSE43777, the program was unable to select any threshold for either study. This happened again when the filtered datasets from CEMiTool were attempted, albeit with lower runtimes (9 min for study GSE18090 and 4 min for study GSE43777). We encountered several other problems, such as confusing command line output; no output plots or complementary analyses; massive cluttering of user’s workspace with no option to redirect the several output files; lack of user tutorial or vignette; and inconsistent naming schemes, resulting in an unpleasant user experience.

Other packages, such as CoXpress, DINGO and DiffCoEx were not considered for benchmarking since they analyze more than 2 groups of samples (Table 1). Given these results, we chose to focus the remainder of our benchmarking on the differences between CEMiTool and WGCNA.

The WGCNA method [5] receives an input “m x n” gene expression matrix, containing n samples under specific conditions and m genes, where each element in the matrix gives the expression of one gene in a particular sample. The correlation between each pair of genes is then transformed into an m x m adjacency matrix through an adjacency function. The adjacency matrix may be signed or unsigned. In the former, correlations in the [− 1, 1] interval are scaled into the [0, 1] interval, while in the latter, negative correlations are made positive. During the process, these values are then raised to a power of β, called the soft-threshold, which effectively adjusts how smoothly the connection strengths transition from their lowest to their highest values. The selection of β directly impacts on how adherent to the scale-free model the network will be. In general, the WGCNA authors recommend to use the “scale-free topology criterion” [5], in which the chosen β value is the one that leads the network’s topology to be, at least approximately, scale-free. Adherence to a scale-free topology is measured by a linear regression fit (R^2^) that quantifies the extent to which the degree distribution of the genes in the network follows a power law. Thus, for WGCNA, the chosen β value is the lowest one with which an R^2^ > 0.85 (or R^2^ > 0.8 in the original paper [5]).

However, the selection of the best soft-threshold is relatively arbitrary and can differ from study to study. By looking at a plot showing R^2^ values for each β ranging from 1 to 20, WGCNA users are required to manually define the value of β by considering the trade-off between R^2^ and connectivity - a higher β may make the network more scale-free, but also lowers the mean connectivity.

Despite the WGCNA authors have demonstrated that networks are relatively robust to the selection of the soft-thresholding parameter [5], a more rigorous framework for the selection of beta is still lacking, being usually defined visually by the user, hindering reproducibility and workflow automation. Although WGCNA provides a function named *pickSoftThreshold* that can automatically select the β value, we have created an alternative algorithm, which is based on the concept of Cauchy sequences [17], that improves the automatic selection of the β value, allowing for more reliable and consistent results (See Methods).

Briefly, our method investigates if all possible pairs of β values (in a certain range) possess a difference between their R^2^ values within a pre-defined range *ϵ*, and selects the first beta value in this sequence to satisfy this property. Moreover, our algorithm allows for a lower threshold for R^2^ (R^2^ > 0.8) when compared to WGCNA default threshold (R^2^ > 0.85) - which, in turn, allows for lower values of β. Once the β value is defined, the remaining steps for creating the modules follow the standard WGCNA procedure.

**Fig. 2:**
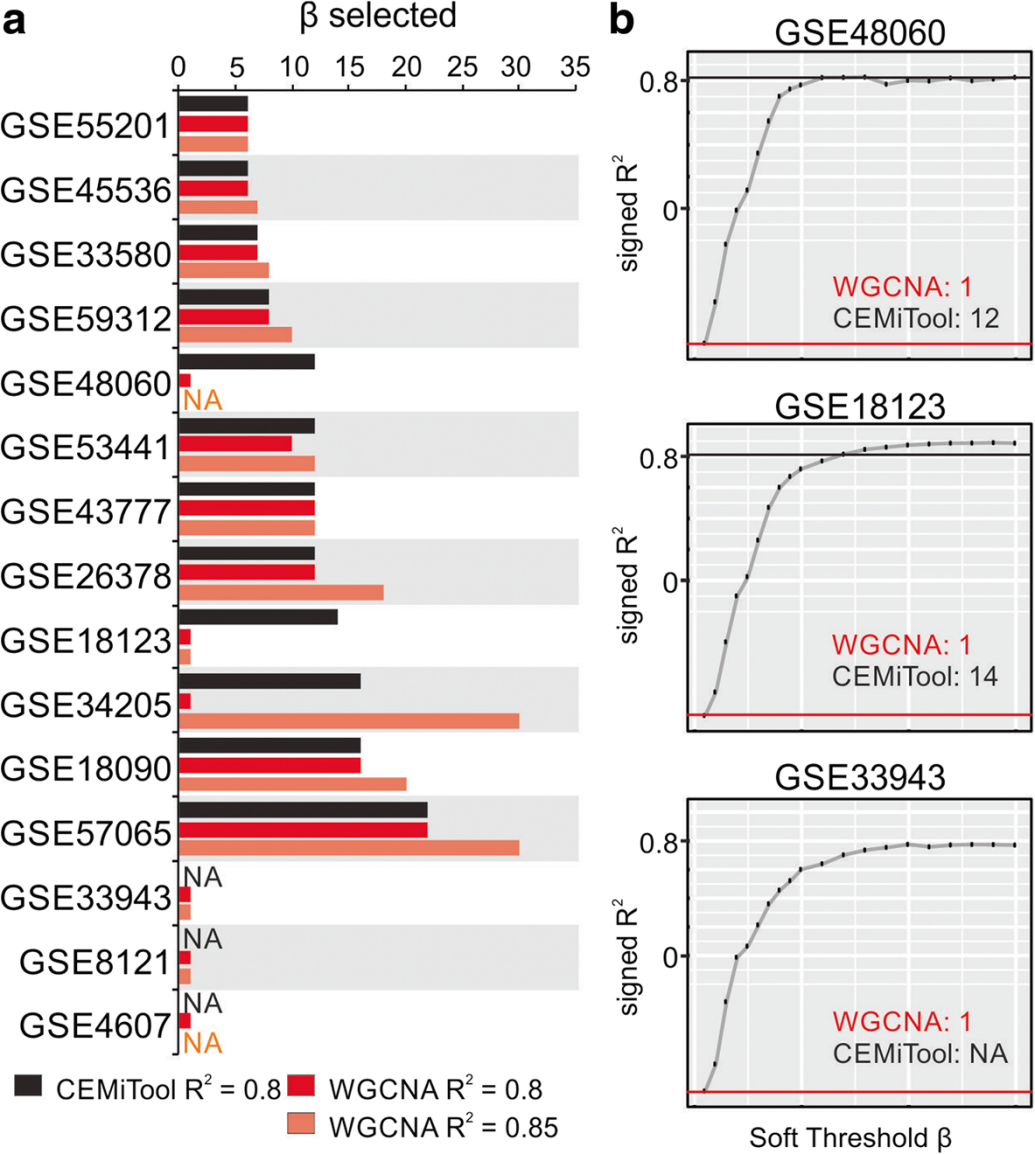
Automatic Selection of Beta parameter. **a** Beta parameters selected by WGCNA (red and brown bars) or CEMiTool (black bars) for 15 microarray studies using the same input genes. **b** β x R^2^ curve for 3 representative studies. Beta values selected by WGCNA (red lines) and by CEMiTool (black line) are shown

To benchmark the selection of β, we compared the method implemented in WGCNA (*pickSoftThreshold* function) with our algorithm (Additional file 1: Text) on 15 publicly available microarray studies. Using the same genes as input, we utilized three different methods for module identification: WGCNA’s *pickSoftThreshold* function with R^2^ values > 0.8 and > 0.85 (WGCNA’s default), as well as CEMiTool’s *cemitool* function with R^2^ > 0.8. Figure 2a shows the value of β for each implementation. With the exception of study GSE53441, the value of β returned by CEMiTool was always equal to or lower than the one returned by WGCNA.

It is worth mentioning that the soft-thresholding impacts not only the network’s topology, but also its information content: the higher the β value, the lower its mean connectivity - since connection strengths in the adjacency matrix are bounded by [0,1] [18]. Consequently, a trade-off between the network’s connectivity and its adherence to a scale-free topology must be considered. Therefore, in the context of this work we consider lower β values to be of more interest than higher values, as long as their R^2^ values are similar.

The difference between WGCNA and CEMiTool in selecting the β parameter can be largely explained by the lower R^2^ threshold implemented in our tool (0.8 in CEMiTool versus 0.85 in WGCNA). We picked this lower R^2^ threshold observing the WGCNA authors’ original recommendation [5]. Also, CEMiTool utilizes a stringent algorithm, based on Cauchy sequences, to select the lowest β parameter that stabilizes the sequence (i.e. keeps its R^2^ values within a pre-defined range), while keeping the R^2^ above the threshold. When the same R^2^ threshold (0.8) is applied, CEMiTool usually returns the same β parameter value as WGCNA’s *pickSoftThreshold* function. In several cases, however, WGCNA returned an inappropriate β value of 1 (Fig. 2).

### Input gene selection

Prior to identifying co-expression modules, it is recommended to filter input genes by either mean expression or variance, rather than by differential expression since this would invalidate the scale-free topology assumption [19]. Nevertheless, the number of genes to be chosen is left undetermined, leading to arbitrary choices that might affect downstream analyses. We thus opted for a flexible, yet objective method of gene selection (Additional file 1: Text). Briefly, by modeling the variance of genes as an inverse gamma distribution, as suggested in [20], we can select genes based on a *p*-value (in our analyses, we set *p* = 0.1 as cutoff). For certain types of RNA-seq data normalizations, our method allows for a correction of the mean-variance dependency [21, 22] by modeling the expression data as a negative binomial distribution [22], and then performing the adequate Variance Stabilizing Transformation (VST) [23] (Additional file 1: Text). To remove potential noise, our package also removes by default the 25% genes with lowest mean expression across all samples prior to filtering.

**Fig. 3:**
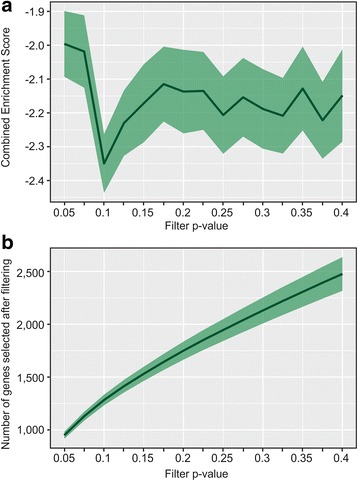
Automating filtering of genes to CEMiTool. The effect of the filter *p*-value threshold on the enrichment of modules was tested by running CEMiTool on 300 studies. **a** The combined enrichment score (CES, see Additional file 1: Text) of the resultant modules for each study and for each filter p-value was calculated using Reactome Pathways as gene sets. The black line represents the mean CES of all 300 studies, while the green shaded area represents the 95% confidence interval from the mean. **b** The number of genes selected for CEMiTool for each filter p-value. The black line represents the mean number of all 300 studies, and the green shaded area represents the 95% confidence interval from the mean

In order to determine the most suitable default filtering parameters, we applied CEMiTool to 300 microarray studies obtained from the GEO (Gene Expression Omnibus [24, 25]) database using differing filter *p*-value thresholds, and assessed the biological significance of the resulting modules (Additional file 2: Table S1). This was determined by calculating the Combined Enrichment Score (CES) of the output modules with respect to the Reactome pathways (Fig. 3). Briefly, the CES allows us to condense the overall enrichment results into a single number - the lower this number is, the more enriched the modules are (Additional file 1: Text). As the filtering *p*-value increases from 0.05 to 0.3, the CES reaches a global minimum at p ≈ 0.1, suggesting that the noise introduced by non-correlated genes outweighs the gain in information (Fig. 3). The filtering p-value is therefore set to 0.1 as a default, but is also easily adjustable by the user via the filter_pval argument to the *cemitool* function to allow the analysis to be more or less stringent, as needed.

### Influence of the number of samples on the scale-free topology model fit

**Fig. 4:**
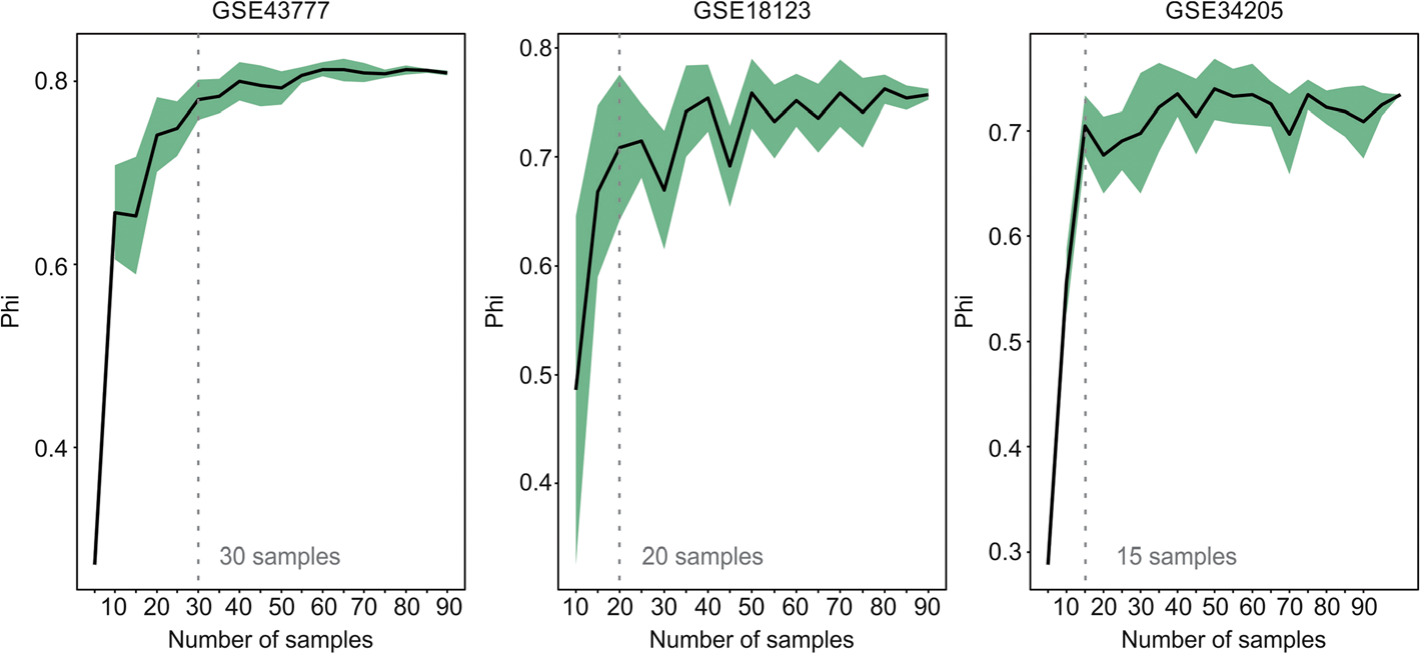
Relationship between the number of samples and phi. Different numbers of random samples from 3 studies (GSE43777, GSE18123 and GSE34205) were picked for CEMiTool analyses. The bold line represents the mean for 10 “sampling” sets. The shaded green area represents the 95% confidence intervals of the mean. The vertical grey line points to the minimum number of samples required to provide a network whose topology does not vary much with increasing sample number, as indicated by the ɸ parameter

To assess the minimum optimal number of samples for analyses, we devised a quality control parameter for the β x R^2^ curve, ɸ . We define ɸ as the ratio of the area under the curve relative to the area of the rectangle made by β × 1, which is the highest possible value for R^2^. Higher values of ɸ mean that the topology of the network converges sharply to a scale-free degree distribution. To estimate the minimum number of samples that returns the highest ɸ value before reaching a plateau, we bootstrapped the number of samples for 3 microarray studies, selecting at first 5 random samples, and then incrementing the sample number by 5 at each step. CEMiTool was run 10 times at each step using default parameters. As shown in Fig. 4, the parameter ɸ tends to stabilize at around 20 samples (which is in accordance to previous findings [26]), indicating that the β x R^2^ curve, and thus network topology, should not vary so much in behavior starting at that sample number.

### Application to RNA-seq datasets

We also ran CEMiTool on 8 RNAseq studies, 4 of which had been previously normalized by log2 CPM (GSE69015, GSE77926, GSE92754, GSE94855), 2 normalized by RPKM (GSE44183 and GSE54456), 1 by FPKM (GSE77564) and 1 only adjusted for fragment and length biases (GSE65540).

**Fig. 5:**
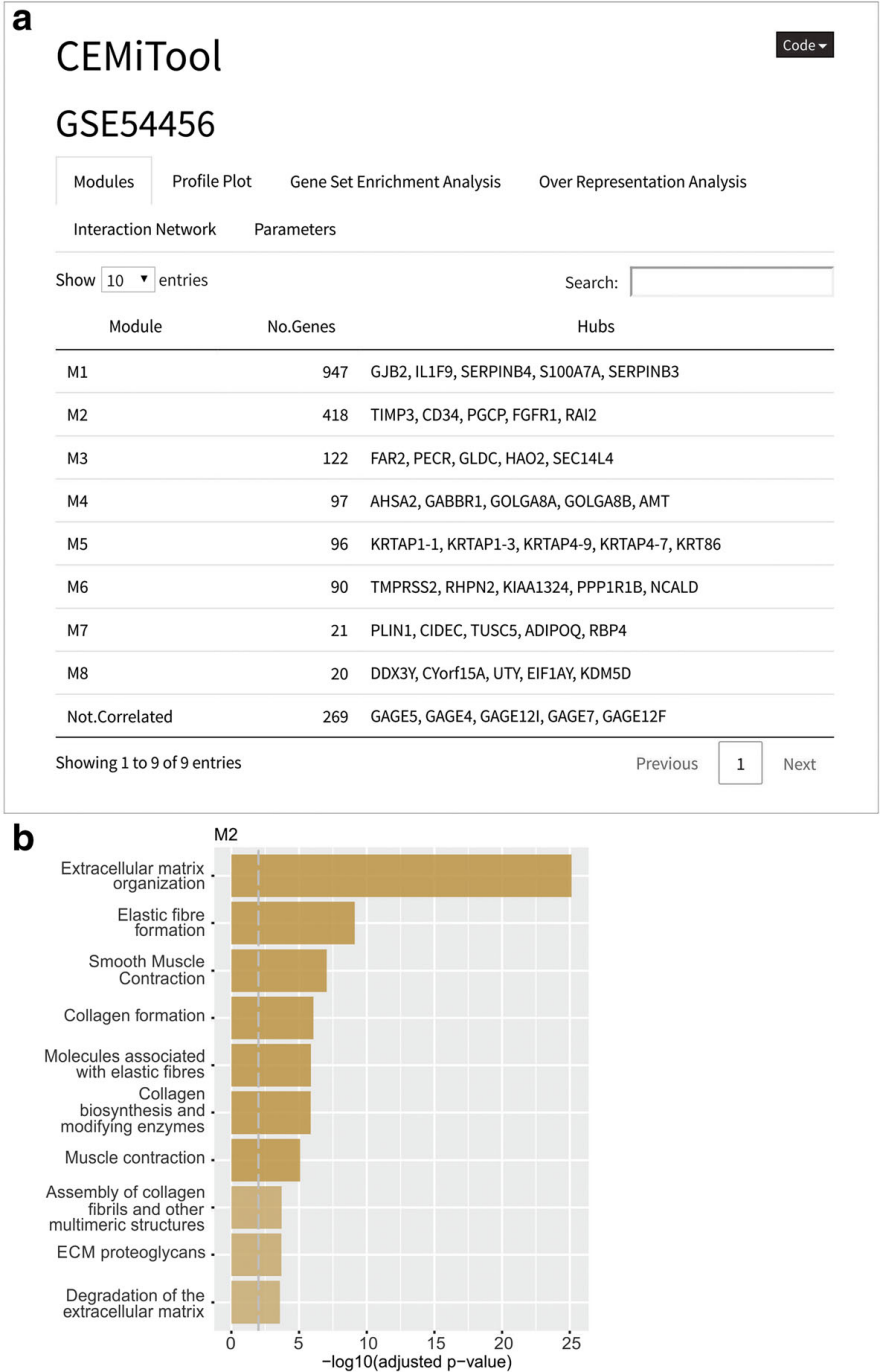
CEMiTool applied to an RNA-seq study of patients with psoriasis. RNA-seq expression data (RPKM normalization) of lesional psoriatic and normal skin samples were download from the GEO database (accession number GSE54456). **a** CEMiTool interactive report showing the results of the main analyses using the optional annotation, pathways and protein-protein interaction files. On the main page, the most connected network hubs can be seen for each module. **b** Significantly enriched pathways for module M2. Metabolic processes such as ‘Extracellular matrix organization’, related to psoriasis, are enriched for module M2

The study GSE54456 [27] has measured 174 transcriptomes of lesional psoriatic and normal skin samples. Among the 8 modules identified by CEMiTool (Fig. 5), the module M1 was enriched for immune system pathways, including interferon alpha signaling, which is known to be related to the disease [28, 29]. One notable hub gene for module M1 was S100A7A. Although this gene was not mentioned in the original publication [27], others have shown that the expression of S100A7A is upregulated in lesioned-skin psoriasis patients [30]. CEMiTool analyses also revealed a module related to extracellular matrix organization and collagen formation (Fig. 5), suggesting that the expression of genes responsible for maintaining the structure of the skin may be coordinately altered by the disease.

### CEMiTool applied to over 1000 publicly available microarray studies

**Fig. 6:**
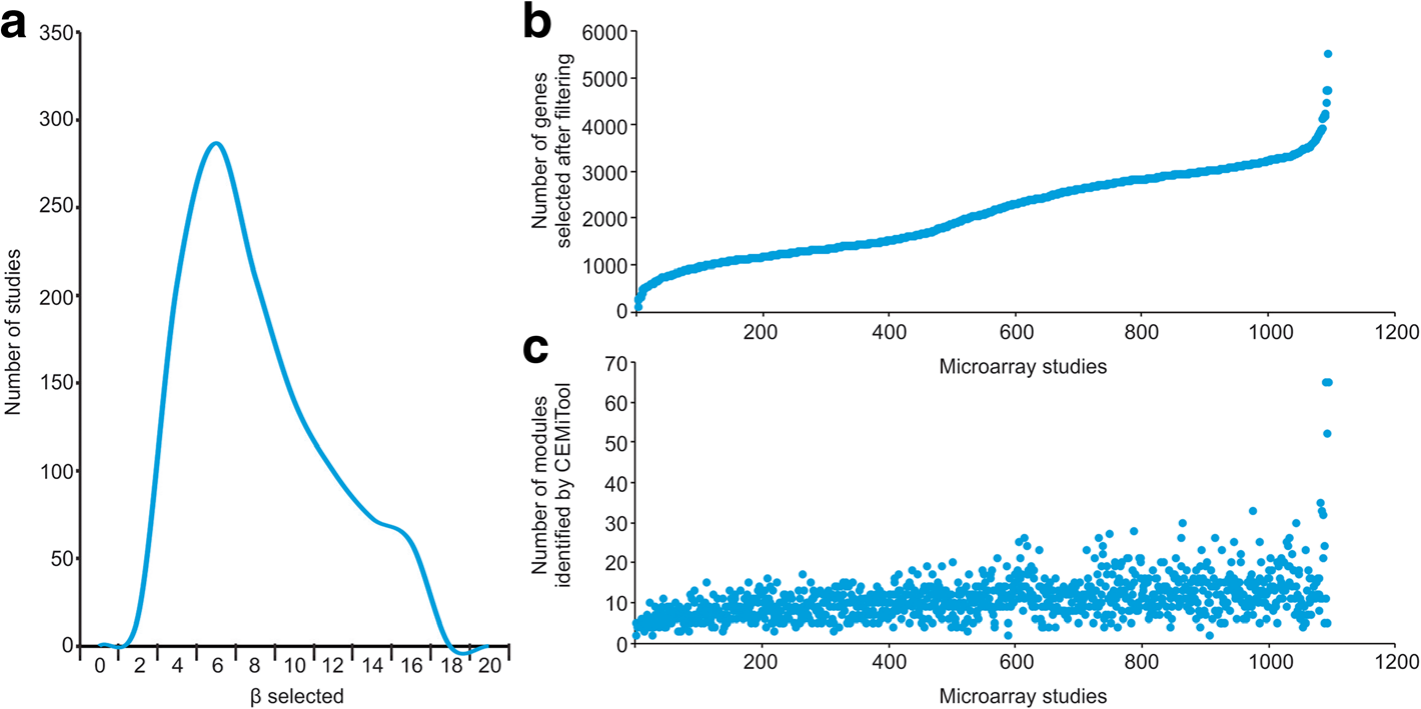
CEMiTool applied to 1000+ microarray studies. **a** Distribution of beta values selected by CEMiTool for all 1.094 studies. **b** Number of genes selected after filtering (*P*-value = 0.1 cutoff). Studies were ordered by the number of genes selected after filtering. **c** Number of modules identified by CEMiTool for each study. Studies are in the same order as in (**b**)

To demonstrate that CEMiTool can be easily automated, we ran the package on 1094 microarray studies obtained from the GEO database. For each study, we downloaded the authors’ normalized data and ran the *cemitool* function using the default parameters. Figure 6 shows the distribution of β values, and the number of modules and filtered genes selected for the analyses.

Almost 12,000 gene modules were identified by CEMiTool, containing in total over 2 million genes. The studies span hundreds of different biological conditions, including cancer, drug treatments, infectious diseases, and inflammatory and neurological pathologies. The list of all studies can be found in Additional file 3: Table S2.

### Applying CEMiTool to study dengue

**Fig. 7:**
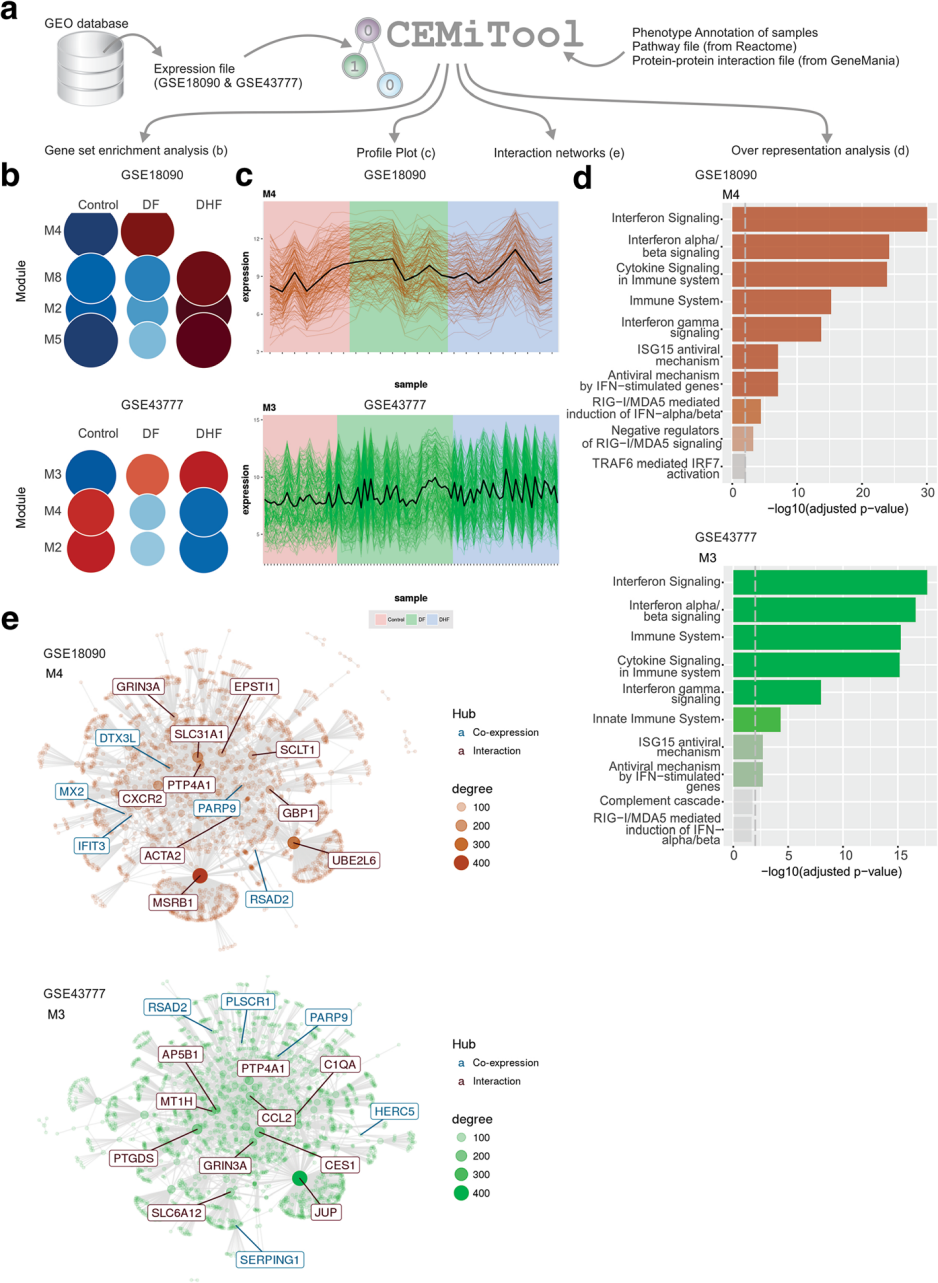
CEMiTool applied to study Dengue infection. **a** Expression data from two microarray studies of patients infected or not with Dengue virus were downloaded from GEO. CEMiTool was independently run on those studies using an annotation file, pathway gene set list and protein-protein interaction file. Selected results of the 4 CEMiTool analyses are displayed in panels (**b**) to (**e**). **b** Gene Set Enrichment Analyses showing the module activity on each class of samples. **c** Profile plots of modules M4 (GSE18090) and M3 (GSE43777). The expression levels of individual genes from each module are shown as colored lines. The black line represents the mean expression of all genes inside the module. Samples are shown in the x-axis and colored by classes. **d** Over Representation Analysis of modules M4 (GSE18090) and M3 (GSE43777). Bar graphs shows the -log_10_ Adjusted P-value of the enrichment between genes in modules and gene sets from Reactome Pathway database. The vertical dashed grey line indicates an adjusted P-value of 0.01. **e** Gene networks of modules M4 (GSE18090) and M3 (GSE43777). The top ten most connected genes (hubs) are labeled and colored based on their “origin”: if originally present in the CEMiTool module, they are colored blue; if inserted from the interactions file, they are colored red. The size of the node is proportional to its degree

To gain novel insights about immunity to infectious diseases, we ran the package on two publicly available microarray studies containing the blood transcriptome of patients infected or not with the Dengue virus (GEO accession numbers GSE18090 and GSE43777). We then annotated samples using the phenotypes provided by the original authors: control (non-infected patients), DF (patients diagnosed as Dengue fever) and DHF (patients diagnosed as dengue hemorrhagic fever). Protein-protein interaction data from GeneMania [31] and gene sets from Reactome Pathways Database [32] were also used in the CEMiTool analyses. The results obtained by such analyses are partially displayed in Fig. 7. The over representation analysis shows that the package identified modules (M4 in GSE18090 and M3 in GSE43777) related to anti-viral immunity, such as interferon signaling and the ISG15 antiviral mechanism. Moreover, Gene Set Enrichment Analyses show that the activity of these modules is higher in DF or DHF compared to Control samples (Fig. 7). Also, the module network graph of study GSE43777 highlights important genes as network hubs, such as CCL2, (coding for chemokine C-C motif ligand 2), which is known to be associated with severe dengue and dengue shock syndrome [33]. However, CCL2 was not highlighted as a key gene for Dengue infection in the original article associated with the study GSE43777 [34].

### Modular analyses of visceral leishmaniasis

**Fig. 8:**
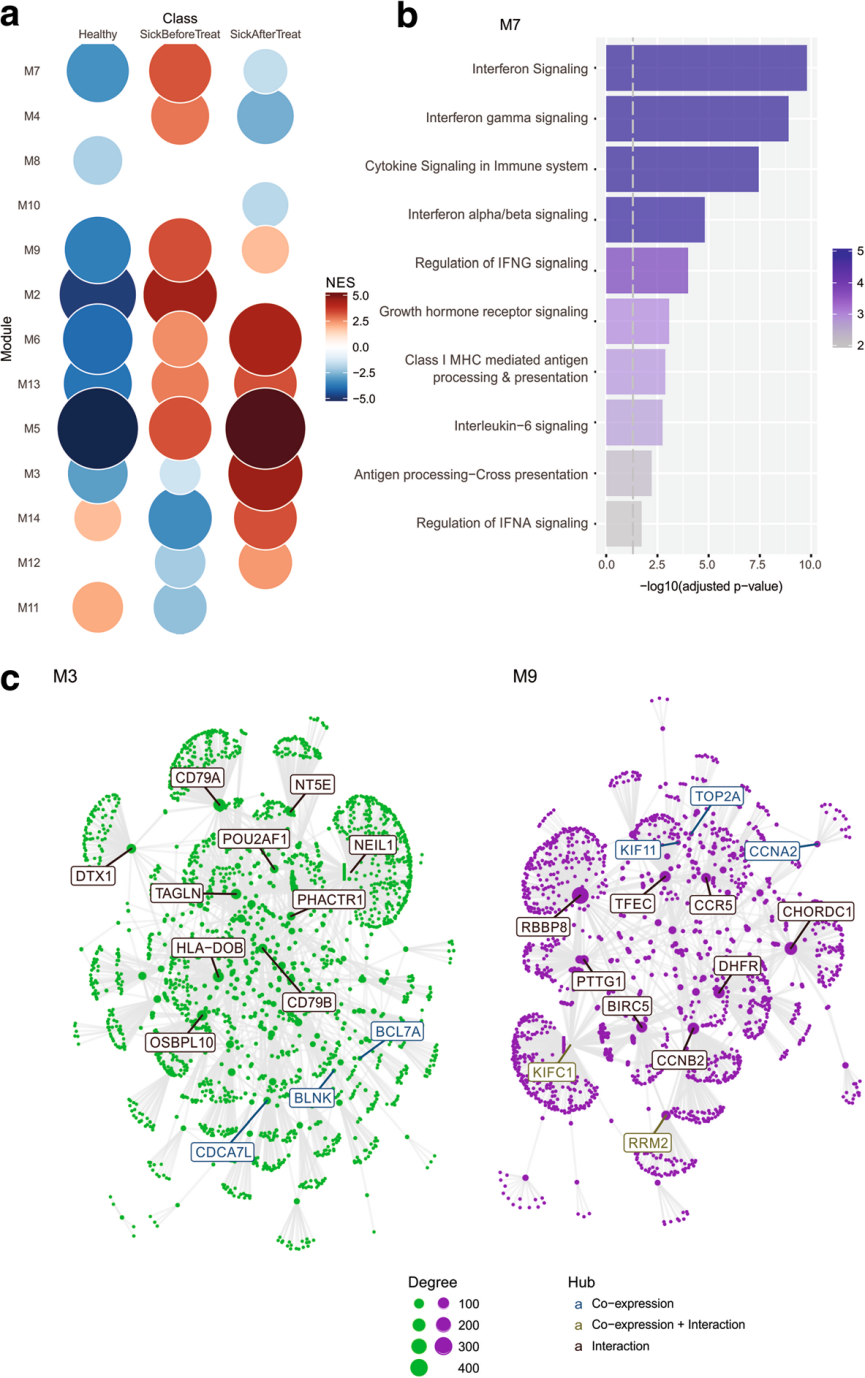
Modular analysis of Leishmaniasis. **a** Gene Set Enrichment Analyses showing the module activity on each class of samples. “Healthy” = uninfected subjects; “SickBeforeTreat” = Leishmania-infected patients before treatment; “SickAfterTreat” = Leishmania-infected patients after treatment. **b** Over Representation Analysis of modules M7. Bar graphs shows the -log_10_ Adjusted P-value of the enrichment between genes in modules and gene sets from Reactome Pathway database. The vertical dashed grey line indicates an adjusted P-value of 0.01. **c** Gene networks of modules M3 and M9. The top ten most connected genes (hubs) are labeled and colored based on their “origin”: if originally present in the CEMiTool module, they are colored blue; if inserted from the interactions file, they are colored red. The size of the node is proportional to its degree

Finally, we used CEMiTool to investigate the blood transcriptome of patients infected with visceral leishmaniasis (VL), a major public health problem in Brazil and worldwide. For this, we performed 17 RNA-seq experiments using the whole-blood obtained from 6 patients infected with *Leishmania infantum*, before and after treatment, as well as 5 uninfected healthy individuals (Additional file 1: Text). CEMiTool has generated 14 modules containing 1700 genes (Fig. 8 and Additional file 4: html report). Of those, modules M7 and M10 refer to interferon-mediated immune responses (IFNgamma and type I IFN, respectively), which are well known to be involved in experimental models of leishmaniasis [35]. However, although IFNgamma response (M7) has been reported in clinical studies as well, little is known about the role of type I IFN (M10) in VL patients. In fact, type I IFN genes are typically elicited in viral infections, and not by protozoan parasites such as *Leishmania infantum*. Further experiments must be conducted to assess how the type I IFN genes may drive the functions of innate and adaptive immune cells during VL infection. Also, CEMiTool was able to unravel the dynamics of genes involved in B cell-mediated immunity during VL treatment, as shown by module M3 (Fig. 8). Integration with protein-protein interaction data revealed *CD79A* and *CD79B* as potential hubs in module M3 (Fig. 8). Both proteins form a dimer associated with the B-cell antigen receptor (BCR), and are critical for B cell immunity. The finding that CD79A and CD79B genes, as well as other members of the modules related to B cell development, are co-expressed and that the module activity is increased on VL treatment demonstrate that CEMiTool can provide new insights about the host response to treatment and to the disease.

Gene set enrichment analysis revealed novel insights about the molecular disturbances caused by the infection (Fig. 8). For instance, the transcriptional activity of module M4, which is enriched by genes associated with “platelet degranulation” and “hemostasis”, is significantly high in VL patients before receiving treatment (Additional file 4: html report). Indeed, VL is associated with several haematological manifestations, including anaemia, leucopenia, and disseminated intravascular coagulation [36]. In addition, the pattern of activity of module M9, which is associated with cell cycle (Additional file 4: html report and Fig. 8), suggests that an intense proliferation of cells expressing CCR5 (such as macrophages, dendritic cells and memory T cells) may be occurring during VL infection. Taken together, our findings may define which genes are driving these haematological manifestations, and thus suggesting effective drug treatments to VL.

## Conclusions

Given the inherently modular profile of biological systems, gene co-expression networks have been extensively used in order to better understand how specific groups of genes are able to orchestrate the several different metabolic pathways present in organisms, as well as identify how they change in response to different conditions and diseases. CEMiTool can identify biologically relevant gene co-expression modules in an automated and easy-to-use way, as well as to perform a comprehensive set of analyses to better understand the biological functions present in the underlying system.

## Methods

### Soft-threshold selection and gene module discovery

Although WGCNA provides a function named *pickSoftThreshold* that can automatically select the soft-threshold β value, we have created an alternative algorithm, which is based on the concept of Cauchy sequences [17], that improves the automatic selection of this parameter, allowing for more reliable and consistent results. Moreover, our algorithm allows a lower threshold for R^2^ (R^2^ > 0.8) when compared to WGCNA’s default threshold (R^2^ > 0.85). This, in turn, allows for lower values of β. Once a β value is chosen, subsequent steps for creating modules follow standard WGCNA procedure.

**Fig. 9:**
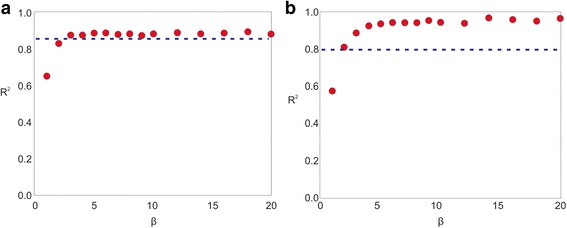
Hypothetical *R*^2^ x β curves for two hypothetical studies. **a** A good choice for β parameter since the corresponding R2 value is above a specified threshold (indicated by the blue line), and subsequent β values brings no additional significant increase in adherence to the scale-free model (**b**) A poor choice for β parameter since even though it’s the smallest possible beta value above the given threshold, it presents significantly less adherence to the scale-free model than subsequent values of beta

Assuming the reader is familiar with the language of Langfelder and Horvath [37], we define the notation *R*^2^(*β*) to denote the value of *R*^2^ obtained for a given *β*. With the *R*^2^ × *β* curve in hand, we should pick a threshold τ. WGCNA’s method consists of taking the smallest *β* such that *R*^2^(*β*) > τ . Suppose that this value is *β*_1_, and it corresponds to *R*^2^ (*β*_1_) = τ + δ for some δ > 0 (i.e., the equivalent *R*^2^ value is only slightly above τ). Take now the next value, *β*_2_. If *R*^2^ (*β*_2_) ≈ *R*^2^ (*β*_1_), then WGCNA’s choice was a good one, as there is little to be gained by raising the soft-threshold any further and much to be lost in terms of network connectivity – this case is shown in Fig. 9. This becomes the majority of cases when τ is close to one, but this comes at the price of unnecessarily high values of β when *R*^2^ → 1 in a slow fashion (or worse, it fails to select a soft-threshold).

On the other hand, selecting a lower value of τ leads to a loss of “scale-freeness” (indicated by *R*^2^) if *R*^2^ (*β*_2_) is significantly larger than *R*^2^ (*β*_1_) – say, *R*^2^ (*β*_2_) = *R*^2^ (*β*_1_) + ∆, for ∆ > δ, as shown in Fig. 9. CEMiTool’s algorithm avoids such cases by interrogating whether | *R*^2^ (*β*_1_) − *R*^2^ (*β*_2_)| < *ϵ* for a pre-defined value of *ϵ* – if not, then CEMiTool rejects *β*_1_ as a soft-threshold and moves on, stopping when the curve appears to stabilize. This means that it exploits all possible significant gains of “scale-freeness” before settling on a value, which allows us to use a lower value of τ without settling for poor values of *R*^2^.

### Availability

The CEMiTool package is available at Bioconductor (DOI: https://doi.org/10.18129/B9.bioc.CEMiTool) and can be downloaded using the command biocLite (“CEMiTool”) (package BiocInstaller v. > = 1.28.0). A Docker image with an environment specifically tailored for CEMiTool analyses is also available at DockerHub (https://hub.docker.com/r/csblusp/cemitool/). RNA-seq data of Leishmania-infected patients have been deposited in the ArrayExpress database at EMBL-EBI under accession number E-MTAB-6137 (https://www.ebi.ac.uk/arrayexpress/experiments/E-MTAB-6137/).

## Additional files

Additional file 1: **Text.** Detailed description of methods. (DOCX 122 kb)

Additional file 2: **Table S1.** List of 300 microarray studies utilized in Fig. 3. (XLSX 99 kb)

Additional file 3: **Table S2.** List of 1000 microarray studies utilized in Fig. 6. (XLSX 37 kb)

Additional file 4: **html report.** CEMiTool output html file using the RNA-seq data of Leishmania-infected patients. (ZIP 14736 kb)

## Abbreviations

BCR: B-cell antigen receptor
CCL2: Chemokine C-C motif ligand 2
CCR5: C-C chemokine receptor type 5
CD79: Cluster of differentiation 79
CEMiTool: Co-expression modules identification tool
CES: Combined enrichment score
CoP: Co-expressed biological processes
CPM: Counts per million
DF: Dengue fever
DHF: Dengue haemorragic fever
diceR: Diverse cluster ensemble in R
DINGO: Differential network analyis in genomics
fgsea: Fast gene set enrichment analysis
FPKM: Fragments per kilobase million
GEO: Gene expression omnibus
GSEA: Gene set enrichment analysis
IFN: Interferon
ORA: Over-representation analysis
RPKM: Reads per kilobase million
VL: Visceral leishmaniasis
VST: Variance stabilizing transformation
WGCNA: Weighted gene co-expression network analysis
