# Supplementary material for: CEMiTool: a Bioconductor package for performing comprehensive modular co-expression analyses
Source: BMC Bioinformatics. 2018 Feb 20;19:56. doi: 10.1186/s12859-018-2053-1 (PMC5819234; doi:10.1186/s12859-018-2053-1)
Supplement: Supplementary file 1 — Text. Detailed description of methods. (DOCX 122 kb) [file 12859_2018_2053_MOESM1_ESM.docx]

**Supplementary Information for:**

**CEMiTool: A Bioconductor package for performing comprehensive modular co-expression analyses**

*Pedro S T Russo****^†,1^****, Gustavo R Ferreira****^†,1^****, Lucas E Cardozo****^1^****, Matheus C Bürger****^1^****, Raul Arias-Carrasco^2^, Sandra R Maruyama^3^, Thiago D C Hirata****^1^****, Diógenes S Lima****^1^****, Fernando M. Passos****^1^****, Kiyoshi F Fukutani^3^, Melissa Lever****^1^****, João S Silva^3^, Vinicius Maracaja-Coutinho^2^, Helder I Nakaya****^1*^***

** Corresponding author: Helder I Nakaya hnakaya@usp.br*

*†These authors contributed equally to this work*

*1 – School of Pharmaceutical Sciences, University of São Paulo, São Paulo, Brazil*

*2 – Centro de Genómica y Bioinformática, Facultad de Ciencias, Universidad Mayor, Santiago, 8580000, Chile*

*3 – Department of Biochemistry, Immunology, and Cell Biology, University of São Paulo, Brazil*

**1. Publicly available transcriptome datasets**

The raw data of each study was downloaded from the Gene Expression Omnibus website (GEO, www.ncbi.nlm.nih.gov/geo/). The arrayQualityMetrics package from Bioconductor (Kauffmann *et al.*, 2009) was applied to each study and samples that failed at least 3 of the 5 tests were excluded. The remaining samples were then normalized by the affy package’s RMA function (Bioconductor) (Gautier *et al.*, 2004). Eight pre-normalized and processed RNAseq studies (GSE69015, GSE77926, GSE92754, GSE94855, GSE44183, GSE54456, GSE77564 and GSE65540) were also downloaded from the GEO database and used as input for CEMiTool.

**2. Variance-based filtering method applied to gene expression**

Let $g_{ij}$ be the expression value of gene $i$ on sample $j$. We assume, as proposed in (Wright and Simon, 2003), that $g_{ij}$ ∼ $N\left( \mu_{i}, \sigma_{i}^{2} \right)$ and that $\sigma_{i}^{2}$ ∼ IG(α, β), with IG denoting the inverse gamma distribution. Then, a p-value is associated to each gene corresponding to the null hypothesis $H_{0}: g_{ij} \sim N\left( \mu_{i}, \sigma_{i}^{2} \right)$, calculated as $p_{i}=Pr[\sigma^{2}\geq s_{i}^{2}]$, where $s_{i}^{2}$ is the estimated variance for the $i$-th gene.

Now, since $\sigma_{i}^{2}$ ∼ IG(α, β), this means that $p_{i}$ can be written as

$$p_{i}=\int_{s_{i}^{2}}^{\infty} f(x|\alpha,\beta)dx$$

where $f(x|\alpha,\beta)$ is the probability density function of an inverse gamma distribution with parameters $\alpha$ and $\beta$. Then, we rewrite $p_{i}$ using the gamma functions:

$$p_{i}=1- \frac{\Gamma\left( \alpha, \frac{b}{x_{i}} \right)}{\Gamma(\alpha)}$$

Here, $\Gamma\left( \cdot, \cdot\right)$ is the upper incomplete gamma function, and $\Gamma\left( \cdot\right)$ is the Euler gamma function. In order to calculate these values, one need only estimate $\alpha$ and $\beta$.

**Parameter Estimation**

We use a method of moments (MM) estimator for alpha and beta, using the fact that $X \sim IG(\alpha, \beta)$ implies $E\left[ X \right]= \frac{\beta}{\alpha-1}$ and $Var[X] = \frac{\beta^{2}}{\left( \alpha-1 \right)^{2}(\alpha-2)}$. Thus, for a sample $(S_{1}^{2}, \ldots, S_{n}^{2})$ of each gene’s estimated variance, the MM gives us:

$$\left\{ \begin{aligned} \bar{S}^{2}= \frac{\hat{\beta}}{\hat{\alpha}-1} \\ Var\left[ S^{2} \right]= \frac{\hat{\beta}^{2}}{\left( \hat{\alpha}-1 \right)^{2}(\hat{\alpha}-2)} \end{aligned} \right.$$

where $\bar{S}^{2}$ denotes the sample mean of $S^{2}$ and $Var[S^{2}]$ is its estimated variance. Then:

$$\left\{ \begin{aligned} \hat{\alpha}= \frac{{\bar{S}^{2}}^{2}}{Var\left[ S^{2} \right]} \\ \hat{\beta}= \bar{S}^{2}(\hat{\alpha}-1) \end{aligned}+2 \right.$$

1. **Considerations for RNA-seq data**

If the expression data were generated by high-throughput sequencing, it might be necessary to remove the mean-variance dependence characteristic of these data (Langfelder and Horvath, 2014; Anders and Huber, 2010).

In order to decide whether this treatment is necessary, a double-log scatter plot of each gene’s mean and variance can be computed, as shown in **Fig. 1**. If the plot shows a strong linear dependence between the two (as is the case in **Fig. 1**), the expression table should be submitted to a Variance Stabilizing Transform [VST; see (Anders and Huber, 2010; Yu, 2009)] before subsequent analyses. This transformation is automatically applied when running the *cemitool* function, but can be turned off by setting the apply_vst argument to FALSE.

**Testing**

Our testing procedure was designed to assess the filter’s impact on the biological aspects of the subsequent analyses. For this purpose, the modules found through CEMiTool were subjected to an Over-Representation Analysis [ORA; see, e.g., (Leong and Kipling, 2009; Huang *et al.*, 2009)] and the obtained p-values were combined and compared by means of the following result:


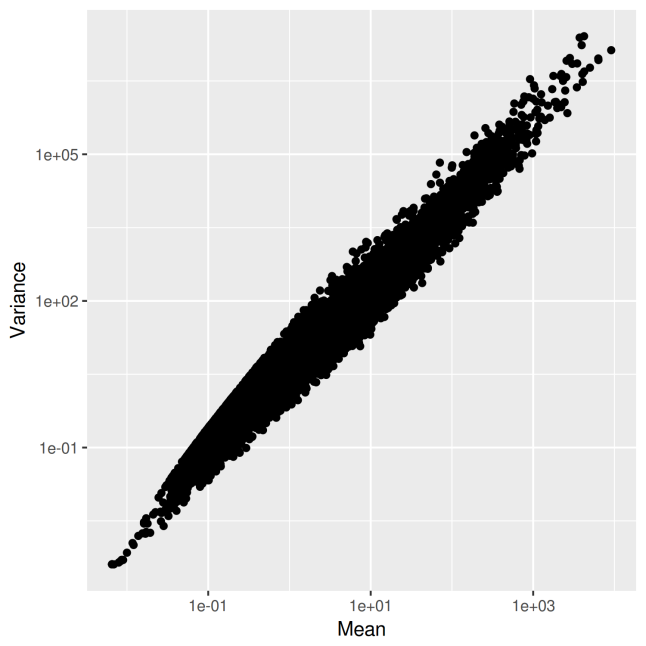

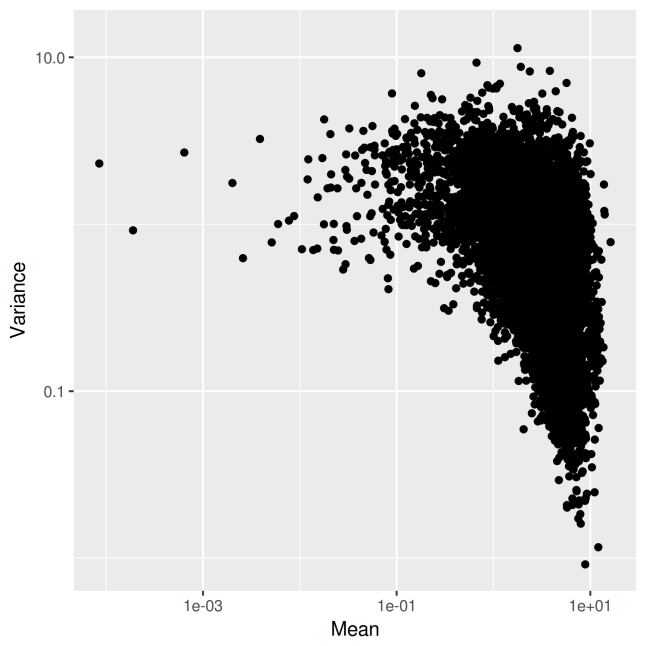


**Figure 1. Mean-variance plots for two studies with different normalizations.** Left: An RPKM-normalized study, with a mean-variance dependence that must be removed. Right: A log2 CPM-normalized study, with a weak mean-variance dependence. No transformation is required.

**Lemma:** Let $p_{jk}$stand for the ORA p-value of the j-th module relative to the k-th

interrogated pathway $(j = 1, 2, . . . , M and k = 1, 2, . . . , L)$. Then, the statistic

$$y= -\sum_{j=1}^{M} \ln\left( 1-\min_{1\leq k\leq L} p_{jk} \right)$$

has a gamma distribution with parameters $M$ and $L$.

**Proof:** According to (Fisher, 1925), for every module $j$ the p-values $p_{j1}, p_{j2}, \ldots, p_{jL}$ follow a uniform distribution in the unit interval. Define now $x_{j} := \min_{1\leq k\leq L} p_{jk}$, and a known theorem on order statistics (see (Ross, 2010)) tells us that $x_{j} \sim Beta(1, L)$for each $j$, and thus − $\ln(1 - x_{j} ) \sim Exp(L)$. Finally, the sum of $M$ i.i.d. exponential distributions with parameter $L$ is a gamma distribution with parameters $M$ and $L$.

Heeding this lemma, the Combined Enrichment Score (CES) defined as $CES= \frac{L}{\sqrt{M}}\left( y-\frac{M}{L} \right)$

has mean zero and unit variance, allowing us to compare across studies with different numbers of modules; since the transformation involved in calculating y is monotonically increasing, the smaller its value, the more significant the modules’ enrichment.

1. **Blood transcriptome of Leishmania-infected Patients**

Whole peripheral blood was collected using BD Vacutainer and PAXgene Blood RNA tubes (PreAnalytix, BD) from healthy control individuals (n=5, average age 23 ± 6.3 years, males). patients with active visceral leishmaniasis (VL-sick: n=6, 3 males and 3 females, average age 14 ± 9 years) and the same VL patients after six months of conventional treatment when they were considered cured (VL-cured). VL diagnosis was confirmed by direct observation of *Leishmania* amastigotes in bone marrow aspirates or positive culture in NNN media (Sigma-Aldrich, St. Louis, MO), or positive rK39 serological test (KalazarDetect Rapid Test: InBios International Inc., Seattle, WA). All procedures involving humans were approved by the Ethics Committee of the University Hospital of the Federal University of Sergipe and Comissão Nacional de Ética em Pesquisa (CONEP). All subjects, or their legal guardians, signed an informed consent form prior to the study.

Total RNA samples were purified with the PAXgene Blood miRNA Kit (PreAnalytix, BD) and the globin mRNA depletion using GLOBINclear-Human kit (Applied Biosystems/Ambion). Depleted total RNA samples were quantified using Qubit® 3.0 Fluorometer (Thermo Fisher Scientific Inc) and RNA integrity was assessed using a lab-on-chip Agilent 2100 Bioanalyzer.

RNA-seq data were generated on Illumina HiSeq 2500 platform (Illumina, San Diego, CA) at the Genomics Center of the Laboratory of Animal Biotechnology, ESALQ, University of São Paulo, Piracicaba, SP, Brazil. Libraries were prepared using TruSeq™ Stranded RNA Sample Preparation kit with Poli(A)+ selection, quantified through qPCR and sequenced using HiSeq SBS V4 kit (2 x 125 bp paired-end reads).

Read quality control of raw sequencing data in fastq files were performed with FastQC [BABRAHAM INSTITUTE] and high quality reads (Phred quality score above 20) trimmed from adaptors and sequencing primers were obtained with filtering using Trimmomatic (BOLGER et al, 2014), which filtered out approximately 8% of low quality reads per fastq file. The reads were mapped to the human reference genome GRCh38 version assembly (Ensembl release 84 provided by GENCODE project available at <http://www.gencodegenes.org/>) and quantified to gene features using STAR (DOBIN et al, 2013). Concordant uniquely mapped reads (approximately of 92% mapped reads) were used for further analyses.

**5. References**

Anders,S. and Huber,W. (2010) Differential expression analysis for sequence count data. *Genome Biol*, **11**, R106.

Bolger, A. M, Lohse, M, Usadel, B. Trimmomatic: a flexible trimmer for Illumina sequence data. Bioinformatics. 2014;30:2114–20.

Dobin, A; Davis, C. A; et al. STAR: ultrafast universal RNA-seq aligner. Bioinformatics. 2013;29:15–21.

Fisher,R.A. (1925) Statistical methods for research workers Oliver and Boyd, Edinburgh, Oliver and Boyd, 1970.

Gautier,L. *et al.* (2004) affy--analysis of Affymetrix GeneChip data at the probe level. *Bioinformatics*, **20**, 307–315.

Huang,D.W. *et al.* (2009) Systematic and integrative analysis of large gene lists using DAVID bioinformatics resources. *Nat Protoc*, **4**, 44–57.

Kauffmann,A. *et al.* (2009) arrayQualityMetrics--a bioconductor package for quality assessment of microarray data. *Bioinformatics*, **25**, 415–416.

Langfelder,P. and Horvath,S. (2014) WGCNA package: Frequently Asked Questions.

Langfelder,P. and Horvath,S. (2008) WGCNA: an R package for weighted correlation network analysis. *BMC Bioinformatics*, **9**, 559.

Leong,H.S. and Kipling,D. (2009) Text-based over-representation analysis of microarray gene lists with annotation bias. *Nucleic Acids Res*, **37**, e79.

Ross,S.M. (2010) A First Course in Probability 9 ed. Pearson Custom Publishing, Boston, MA; Pearson Custom Publishing.

Wright,G.W. and Simon,R.M. (2003) A random variance model for detection of differential gene expression in small microarray experiments. *Bioinformatics*, **19**, 2448–2455.

Yu,G. (2009) Variance stabilizing transformations of Poisson, binomial and negative binomial distributions. *Stat Probab Lett*, **79**, 1621–1629.
