# Supplementary material for: CEMiTool: a Bioconductor package for performing comprehensive modular co-expression analyses
Source: BMC Bioinformatics. 2018 Feb 20;19:56. doi: 10.1186/s12859-018-2053-1 (PMC5819234; doi:10.1186/s12859-018-2053-1)
Supplement: Supplementary file 4 — html report. CEMiTool output html file using the RNA-seq data of Leishmania-infected patients. (ZIP 14736 kb) [file 12859_2018_2053_MOESM4_ESM.zip › no_asymp_zscore_pval0.2.html]

CEMiTool


Code 

- Show All Code
- Hide All Code

# CEMiTool

# Study

## Modules

## Profile Plot

## Gene Set Enrichment Analysis

## Over Representation Analysis

### M1

### M2

### M3

### M4

### M5

### M6

### M7

### M8

### M9

### M10

### M11

### M12

### M13

### M14

## Interaction Network

## Parameters
