# Supplementary material for: CEMiTool: a Bioconductor package for performing comprehensive modular co-expression analyses
Source: BMC Bioinformatics. 2018 Feb 20;19:56. doi: 10.1186/s12859-018-2053-1 (PMC5819234; doi:10.1186/s12859-018-2053-1)
Supplement: Supplementary file 4 — html report. CEMiTool output html file using the RNA-seq data of Leishmania-infected patients. (ZIP 14736 kb) [file 12859_2018_2053_MOESM4_ESM.zip › Leishmania_Report.pdf]

CEMiTool

Contents

1 Study **1**
1.1 Modules 1
1.2 Profile Plot 1
1.3 Gene Set Enrichment Analysis 10
1.4 Over Representation Analysis 11
1.5 Interaction Network 25
1.6 Parameters 31

1 Study

1.1 Modules

1.2 Profile Plot

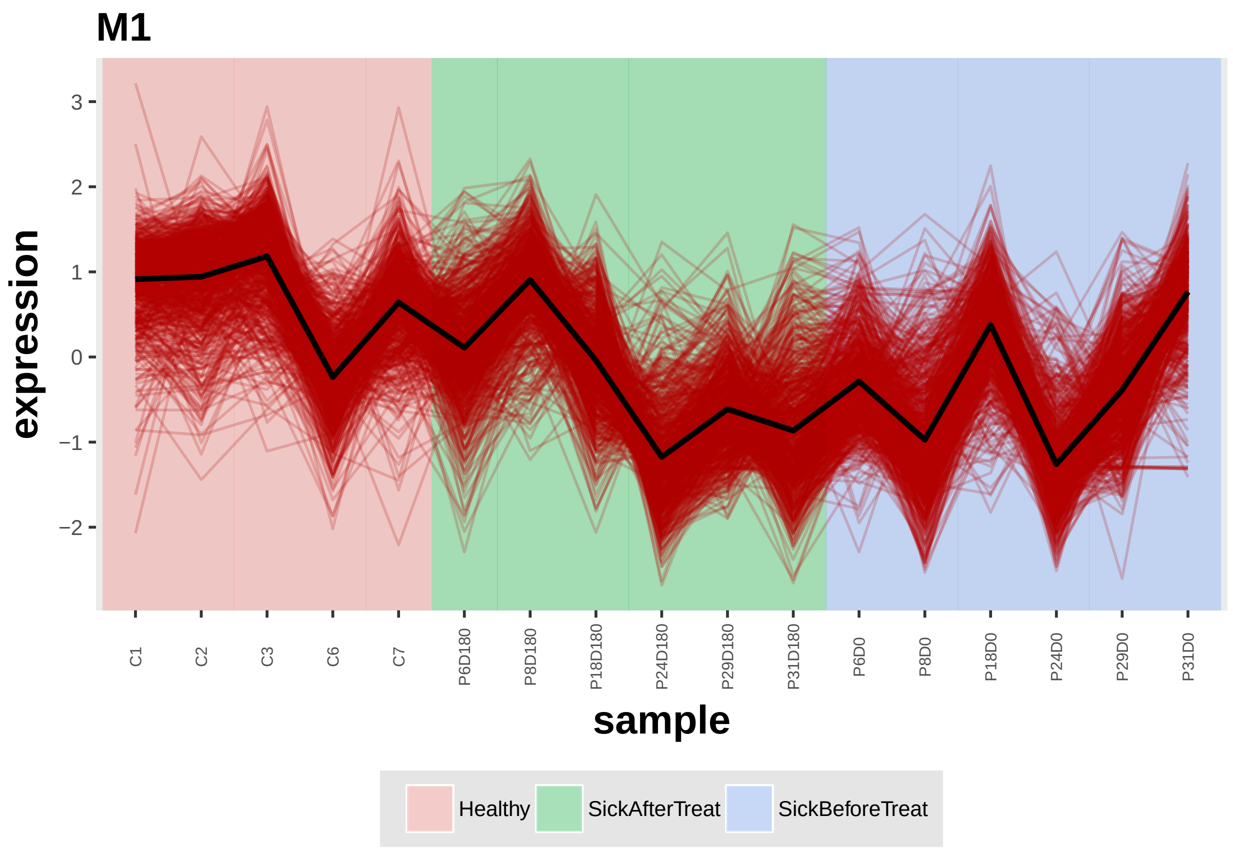

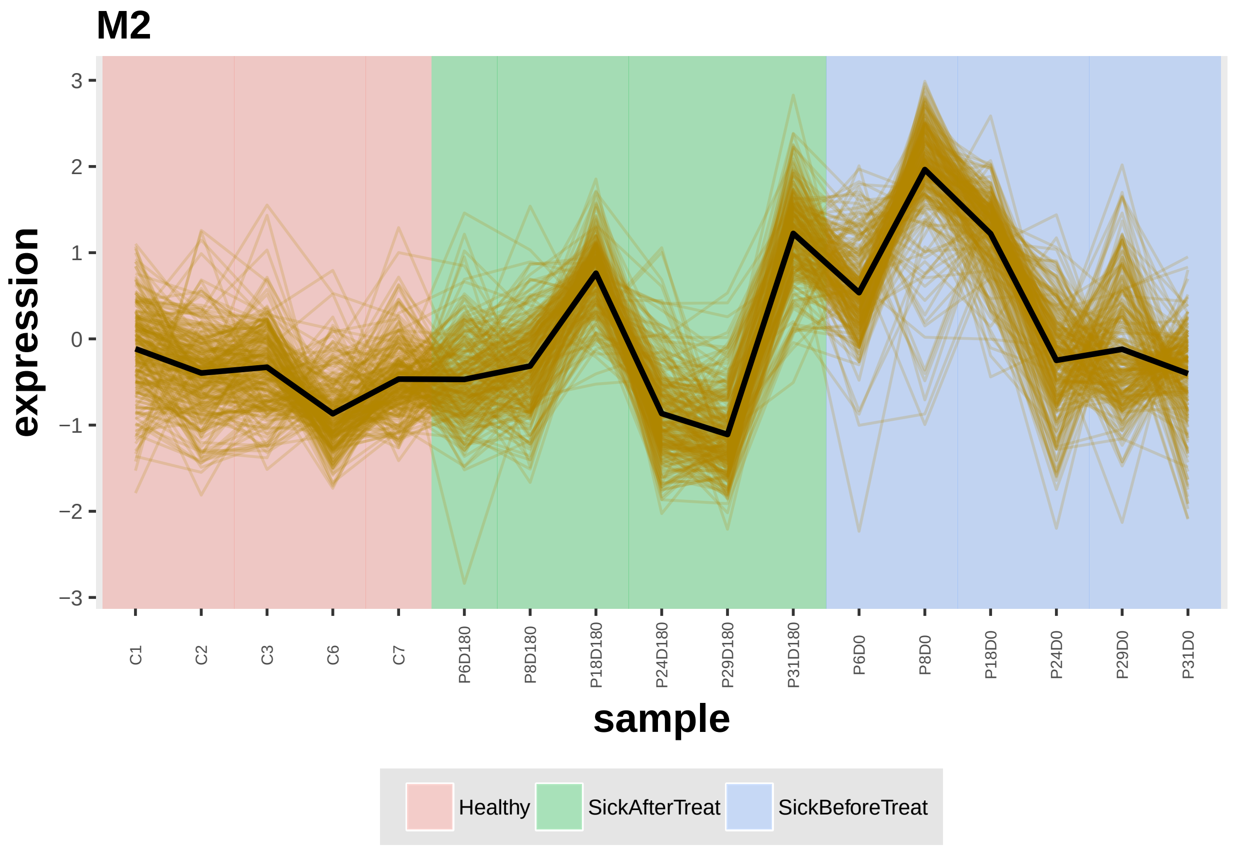
M2
expression
3
2
1
0
−1
−2
−3
C1
C2
C3
C6
C7
P6D180
P8D180
P18D180
P24D180
P29D180
P31D180
P6D0
P8D0
P18D0
P24D0
P29D0
P31D0
sample
Healthy
SickAfterTreat
SickBeforeTreat

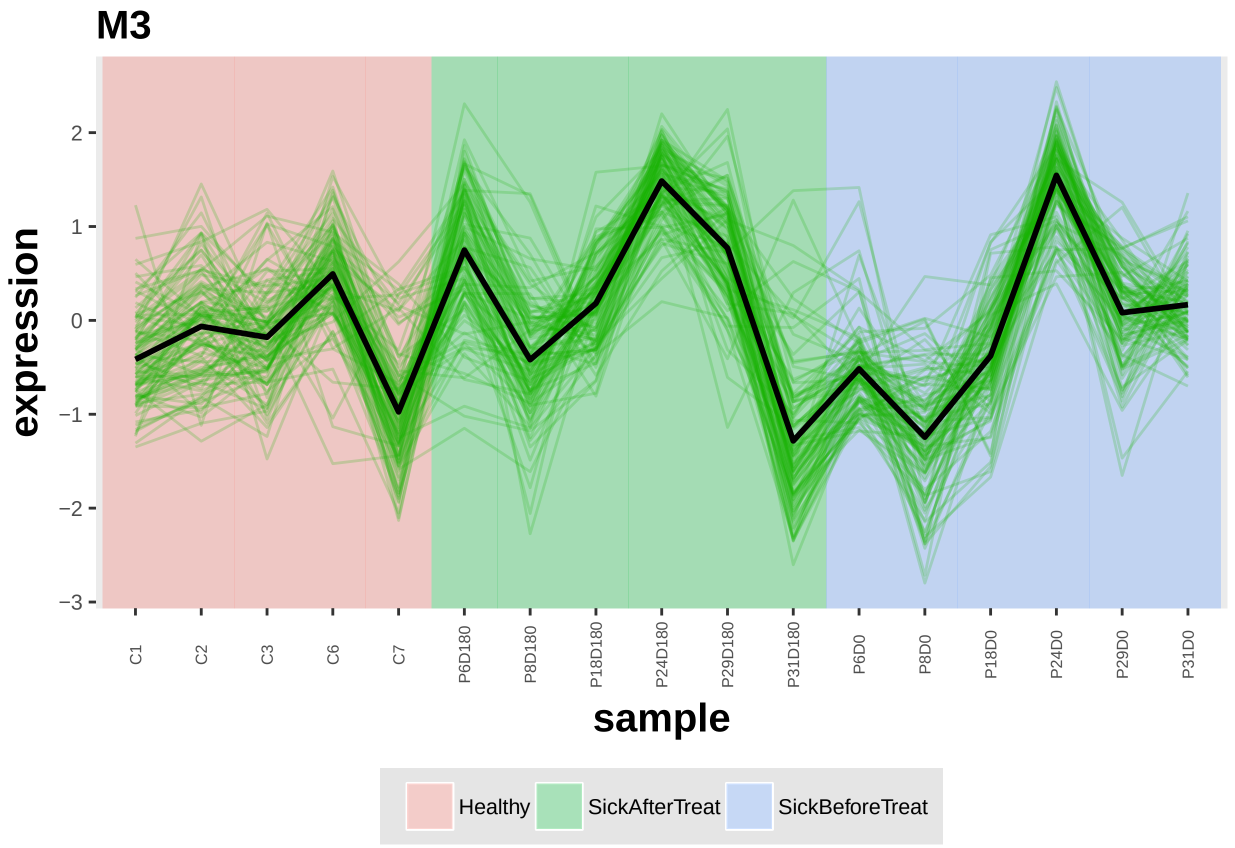
M3
expression
2
1
0
−1
−2
−3
C1
C2
C3
C6
C7
P6D180
P8D180
P18D180
P24D180
P29D180
P31D180
P6D0
P8D0
P18D0
P24D0
P29D0
P31D0
sample
Healthy
SickAfterTreat
SickBeforeTreat

M4

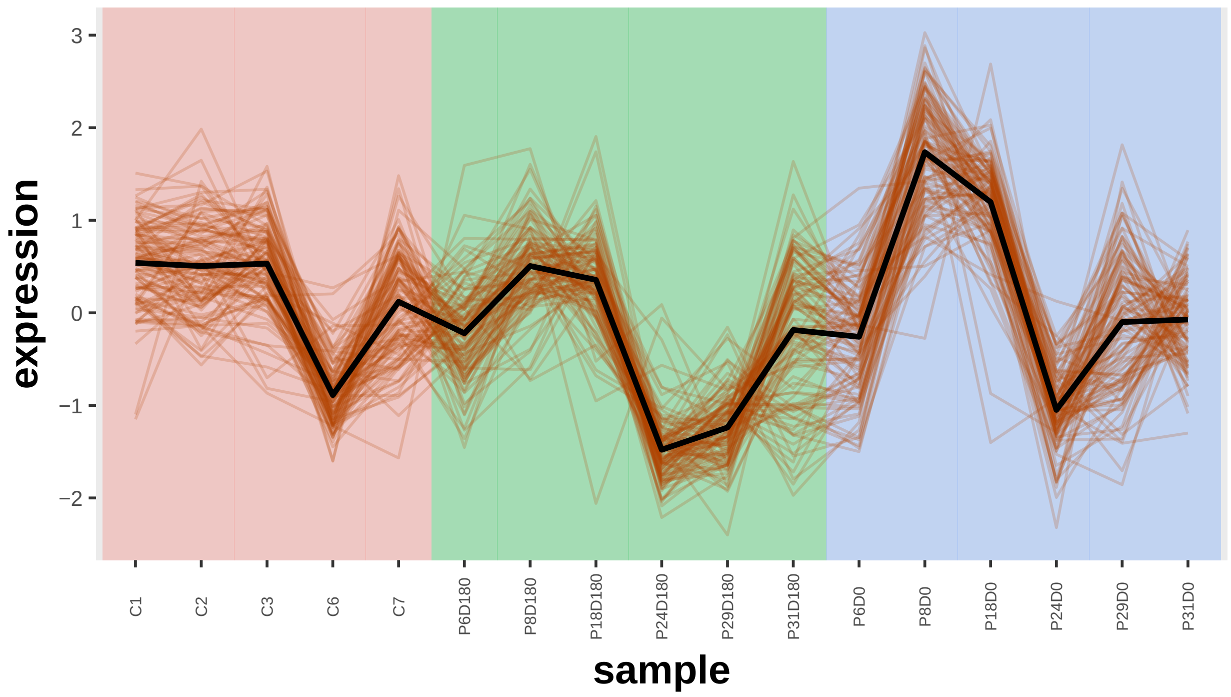

Healthy SickAfterTreat SickBeforeTreat

M5

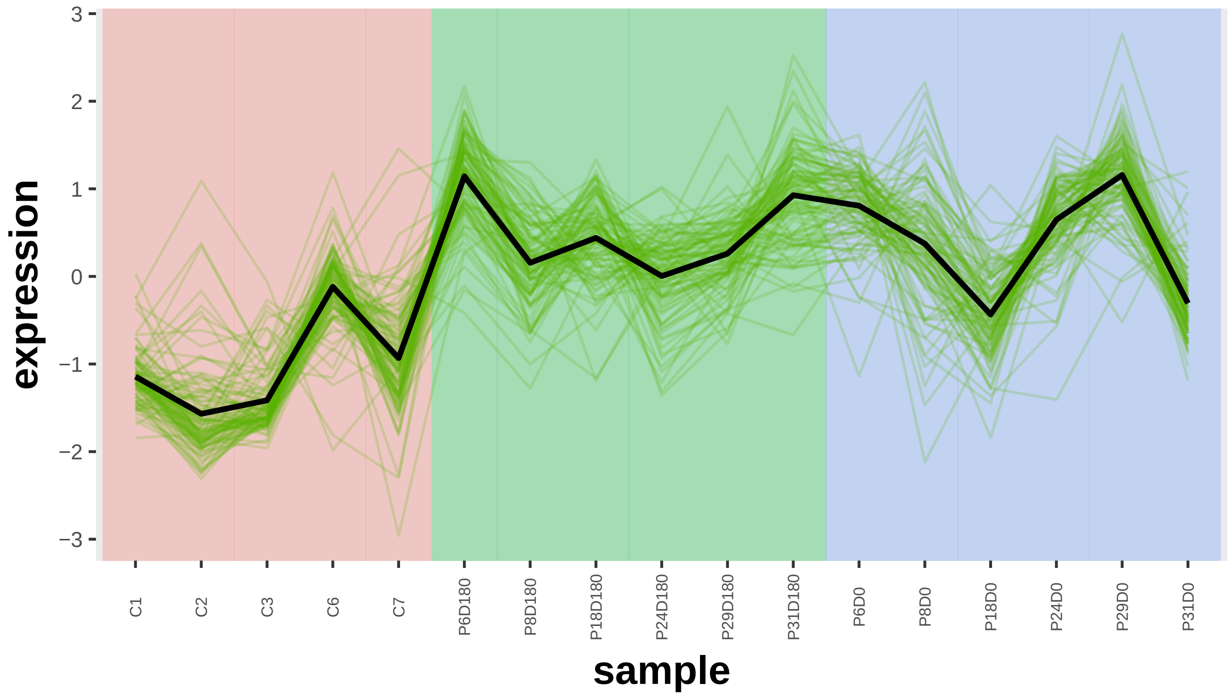

Healthy SickAfterTreat SickBeforeTreat

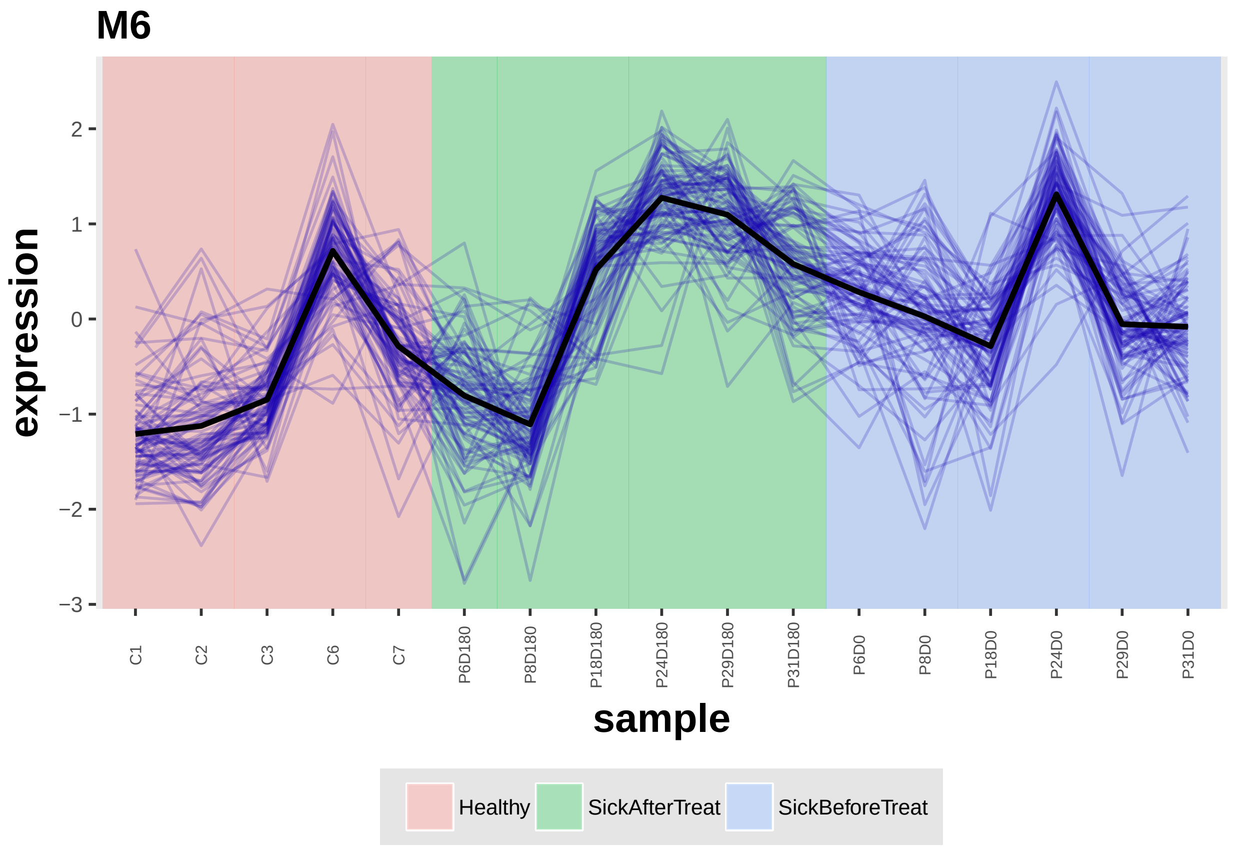
M6
expression
2
1
0
−1
−2
−3
C1
C2
C3
C6
C7
P6D180
P8D180
P18D180
P24D180
P29D180
P31D180
P6D0
P8D0
P18D0
P24D0
P29D0
P31D0
sample
Healthy
SickAfterTreat
SickBeforeTreat

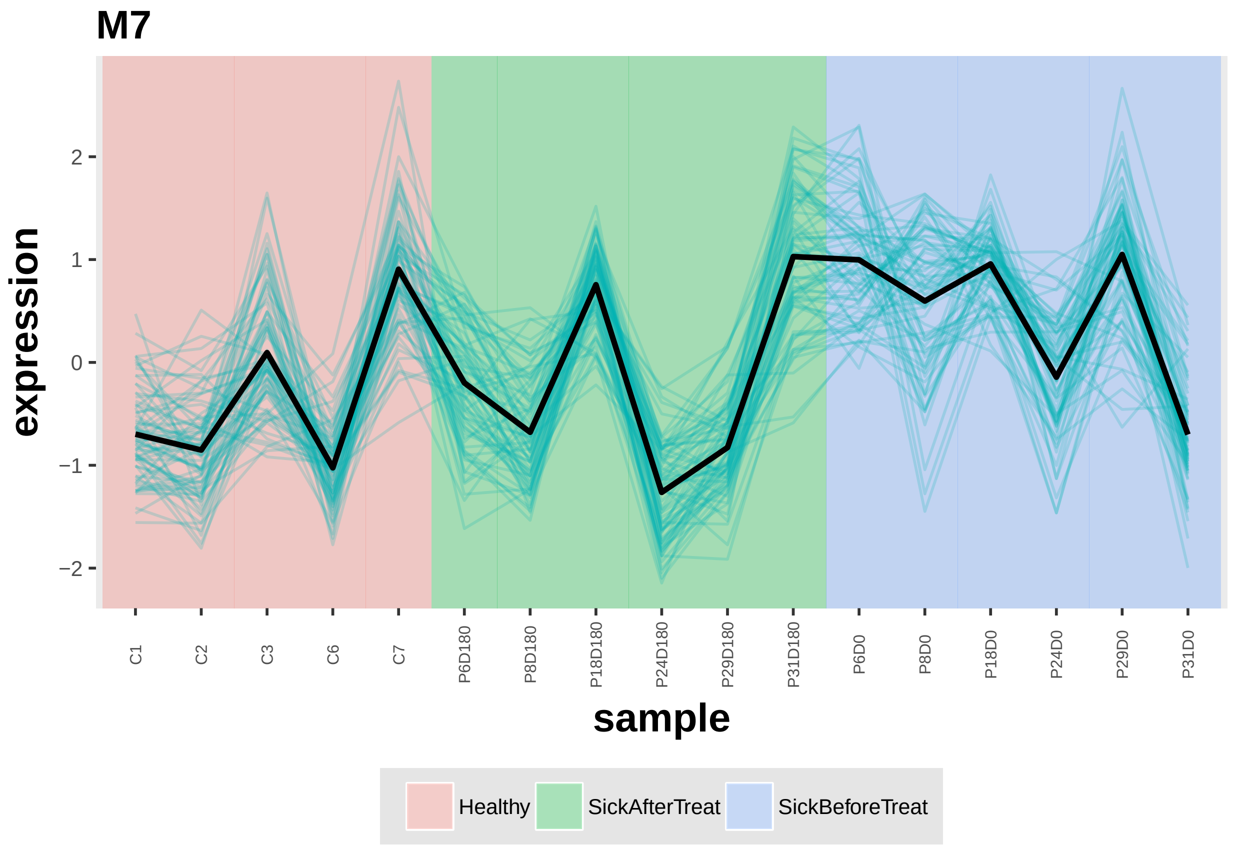
M7
expression
2
1
0
−1
−2
C1
C2
C3
C6
C7
P6D180
P8D180
P18D180
P24D180
P29D180
P31D180
P6D0
P8D0
P18D0
P24D0
P29D0
P31D0
sample
Healthy
SickAfterTreat
SickBeforeTreat

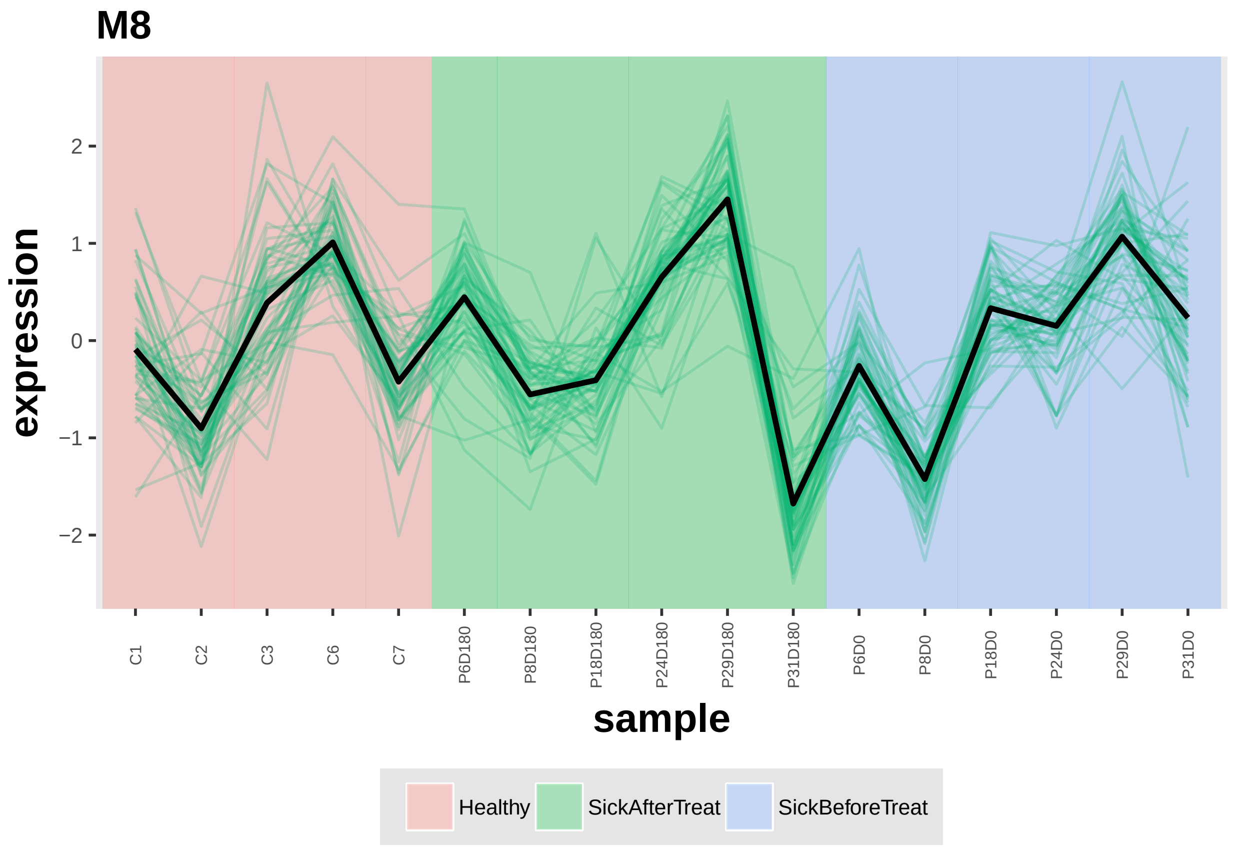
M8
expression
2
1
0
−1
−2
C1
C2
C3
C6
C7
P6D180
P8D180
P18D180
P24D180
P29D180
P31D180
P6D0
P8D0
P18D0
P24D0
P29D0
P31D0
sample
Healthy
SickAfterTreat
SickBeforeTreat

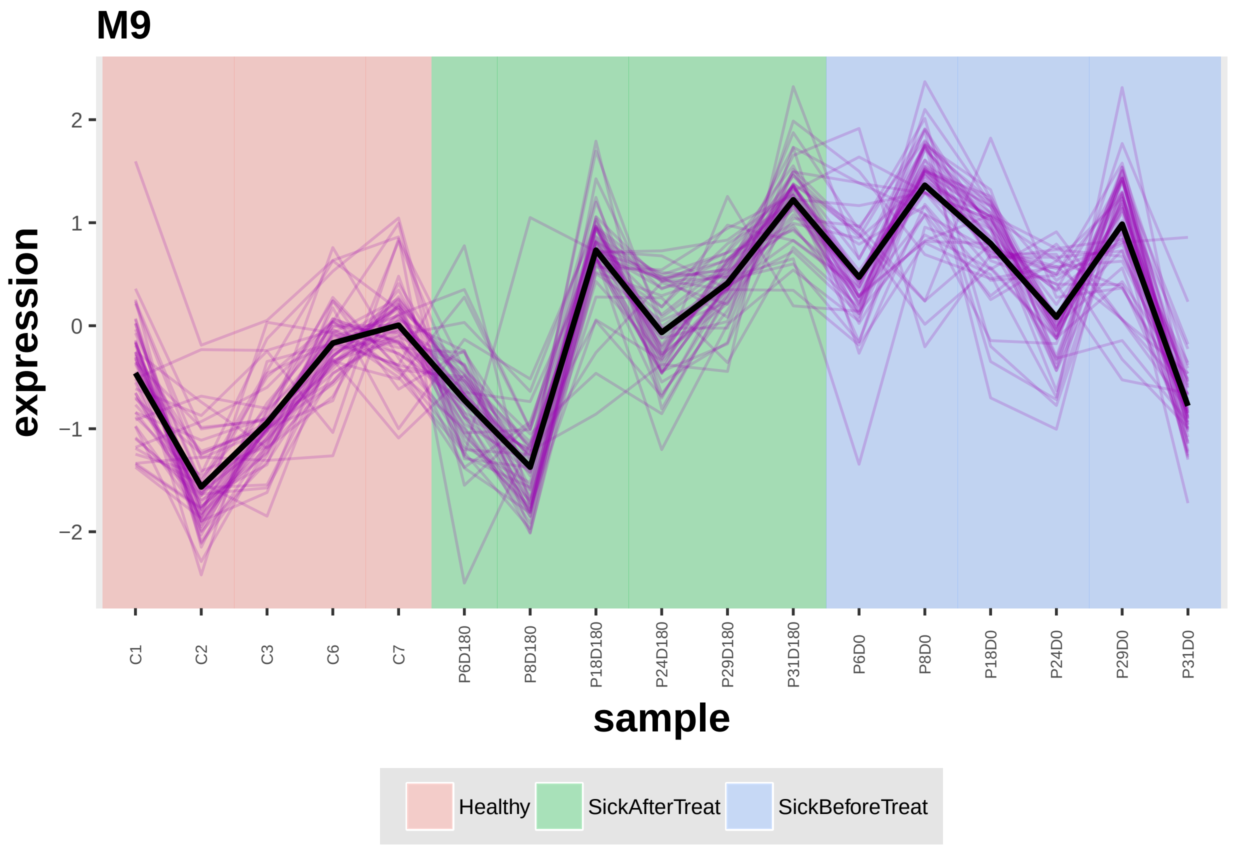
M9
expression
2
1
0
−1
−2
C1
C2
C3
C6
C7
P6D180
P8D180
P18D180
P24D180
P29D180
P31D180
P6D0
P8D0
P18D0
P24D0
P29D0
P31D0
sample
Healthy
SickAfterTreat
SickBeforeTreat

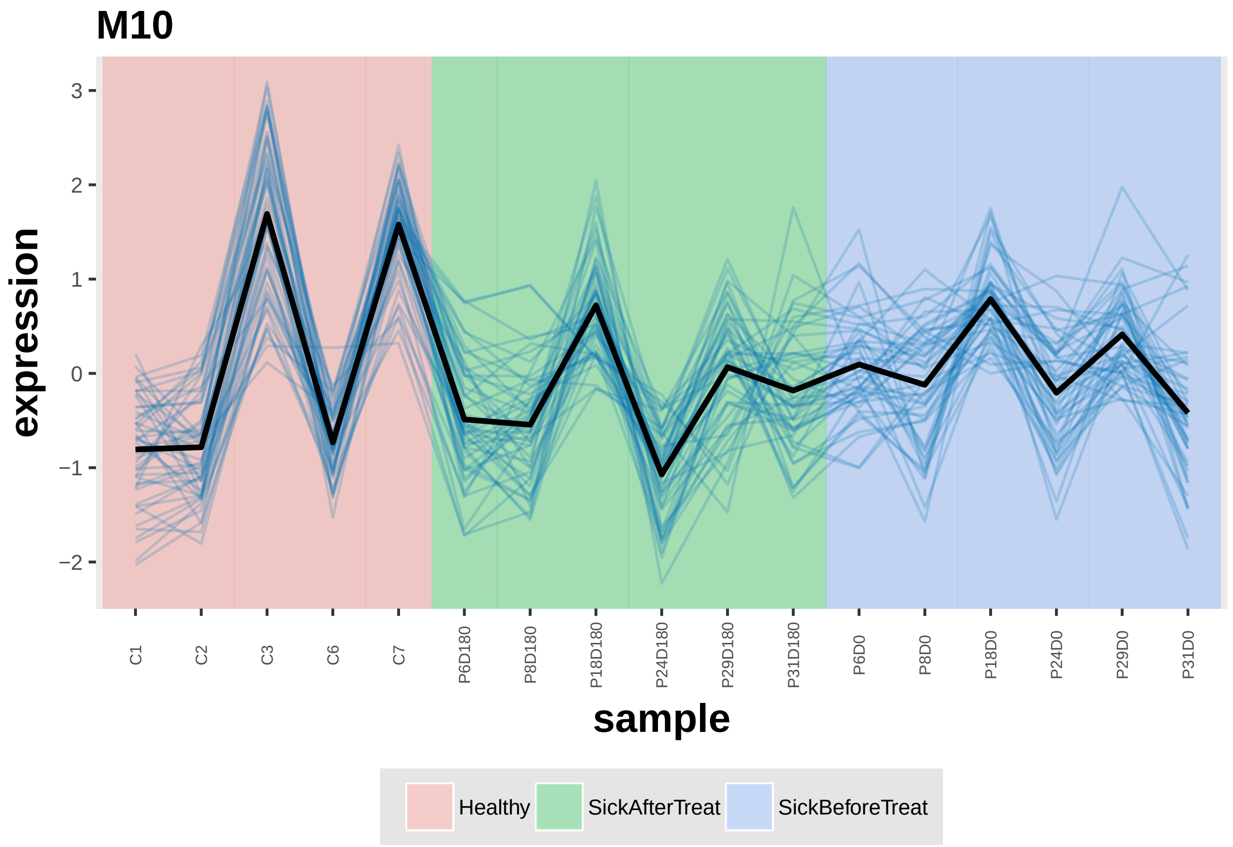
M10
expression
3
2
1
0
−1
−2
C1
C2
C3
C6
C7
P6D180
P8D180
P18D180
P24D180
P29D180
P31D180
P6D0
P8D0
P18D0
P24D0
P29D0
P31D0
sample
Healthy
SickAfterTreat
SickBeforeTreat

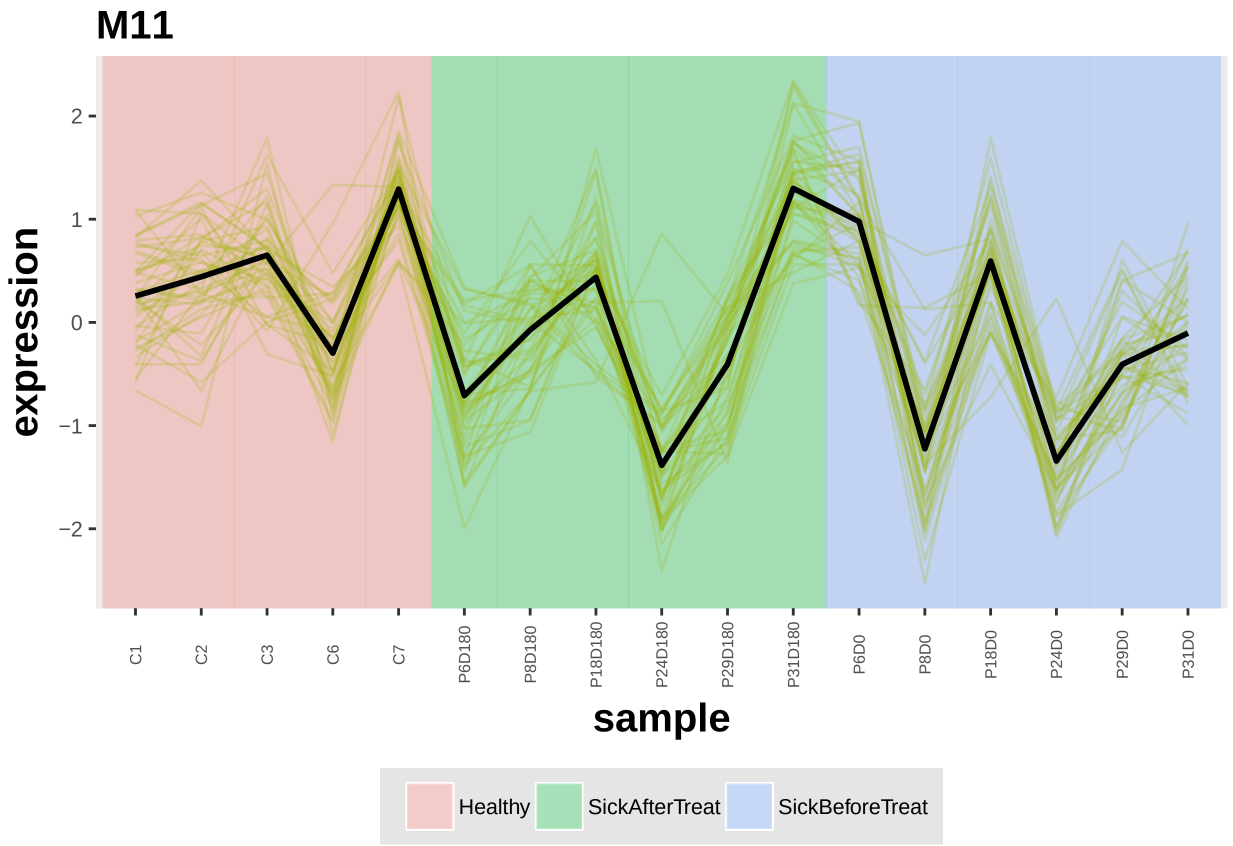
M11
expression
2
1
0
−1
−2
C1
C2
C3
C6
C7
P6D180
P8D180
P18D180
P24D180
P29D180
P31D180
P6D0
P8D0
P18D0
P24D0
P29D0
P31D0
sample
Healthy
SickAfterTreat
SickBeforeTreat

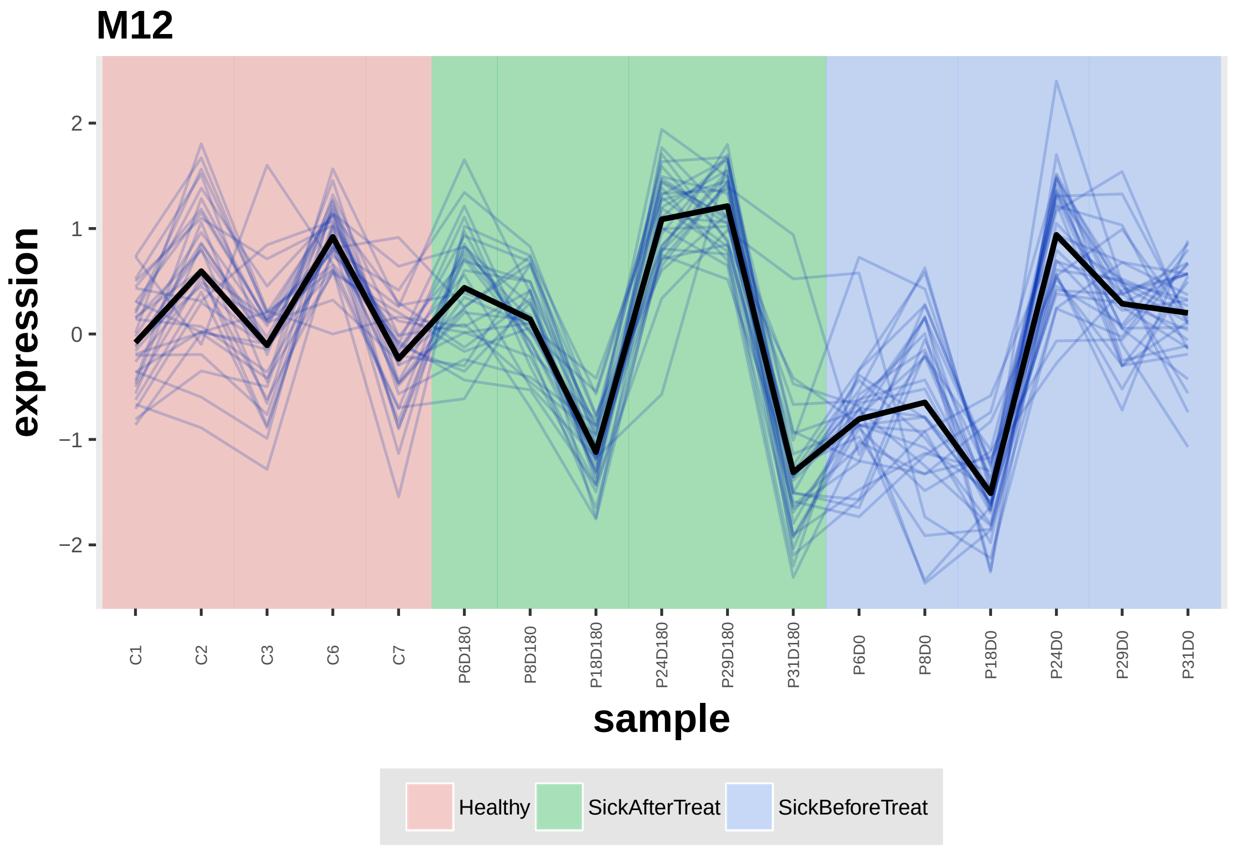
M12
expression
2
1
0
−1
−2
C1
C2
C3
C6
C7
P6D180
P8D180
P18D180
P24D180
P29D180
P31D180
P6D0
P8D0
P18D0
P24D0
P29D0
P31D0
sample
Healthy
SickAfterTreat
SickBeforeTreat

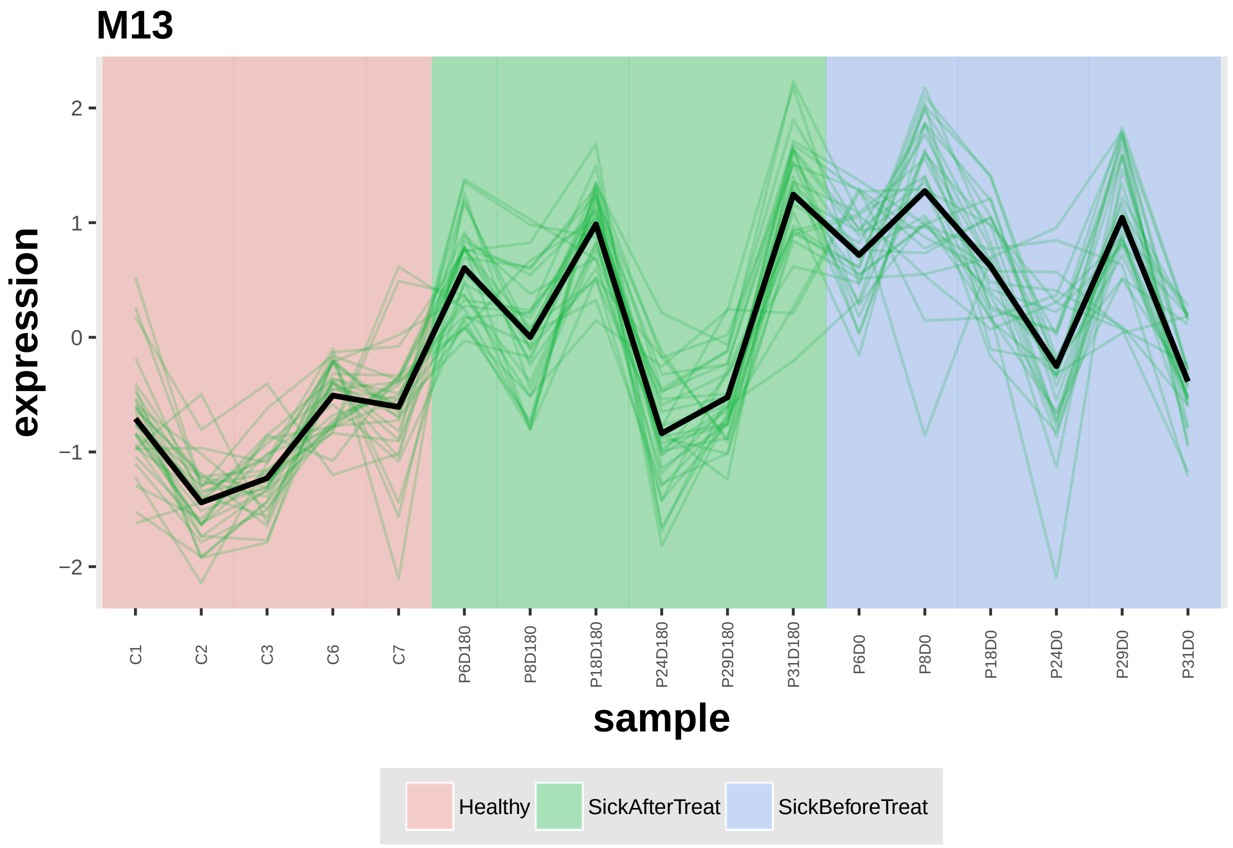
M13
expression
2
1
0
−1
−2
C1
C2
C3
C6
C7
P6D180
P8D180
P18D180
P24D180
P29D180
P31D180
P6D0
P8D0
P18D0
P24D0
P29D0
P31D0
sample
Healthy
SickAfterTreat
SickBeforeTreat

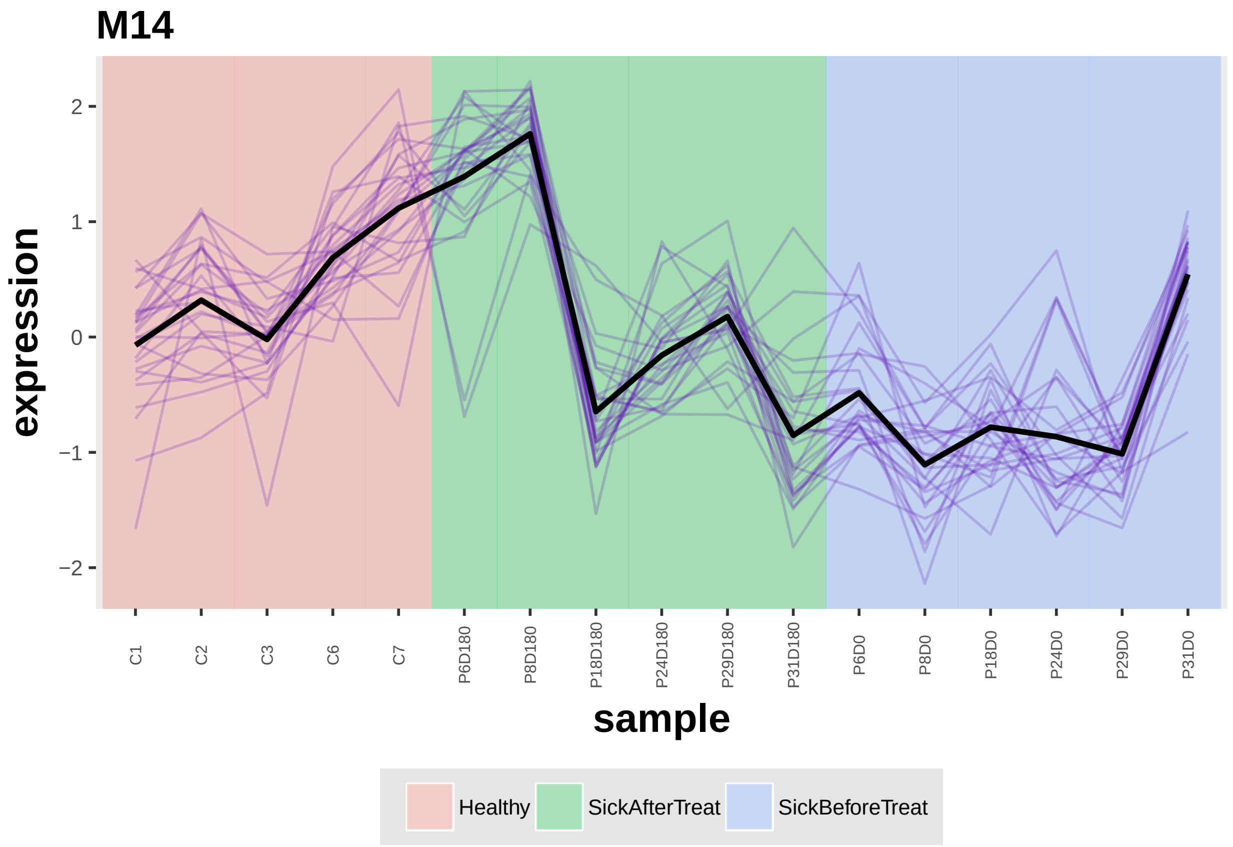
M14
expression
2
1
0
−1
−2
C1
C2
C3
C6
C7
P6D180
P8D180
P18D180
P24D180
P29D180
P31D180
P6D0
P8D0
P18D0
P24D0
P29D0
P31D0
sample
Healthy
SickAfterTreat
SickBeforeTreat

1.3 Gene Set Enrichment Analysis

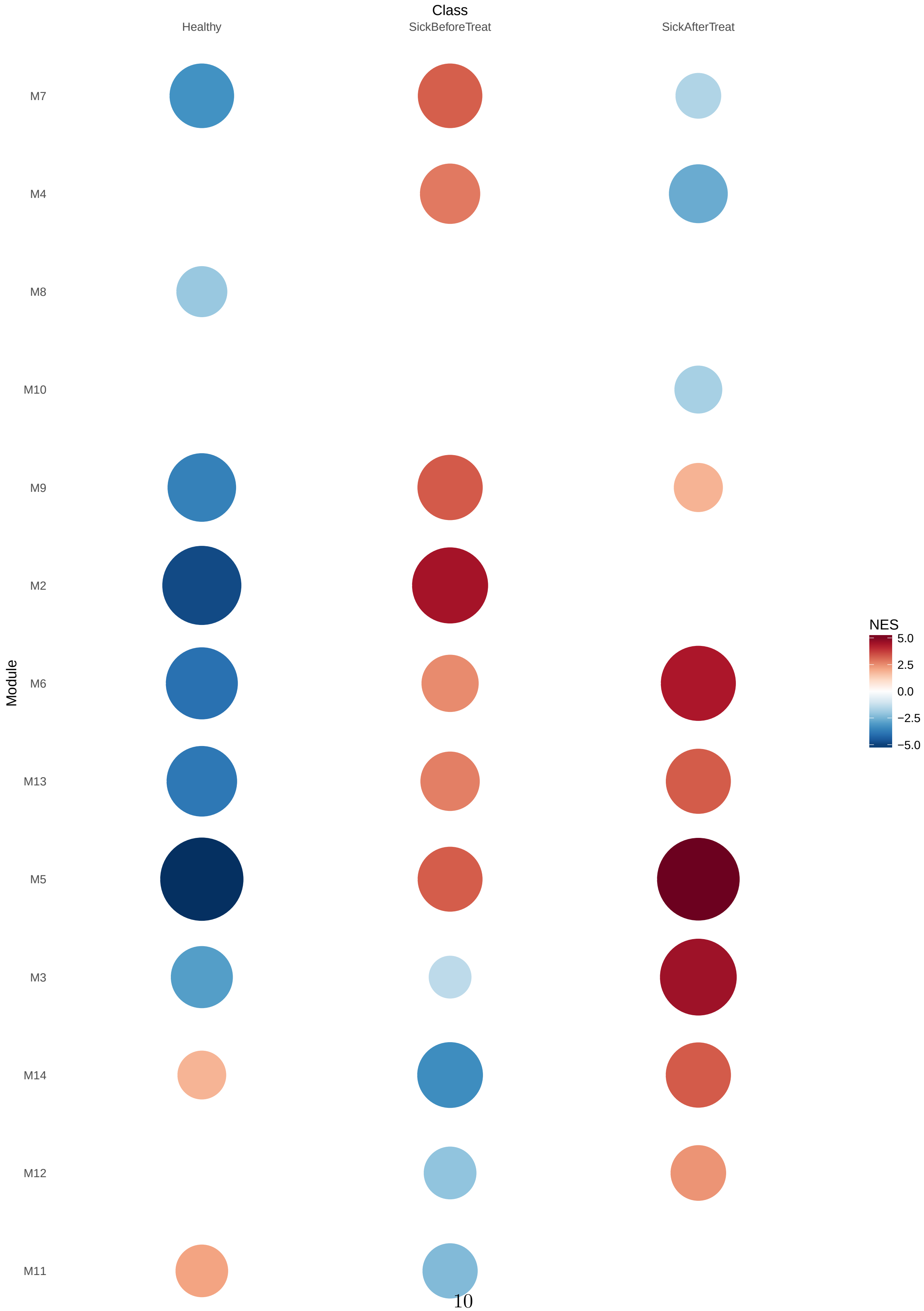

1.4 Over Representation Analysis

1.4.1 M1

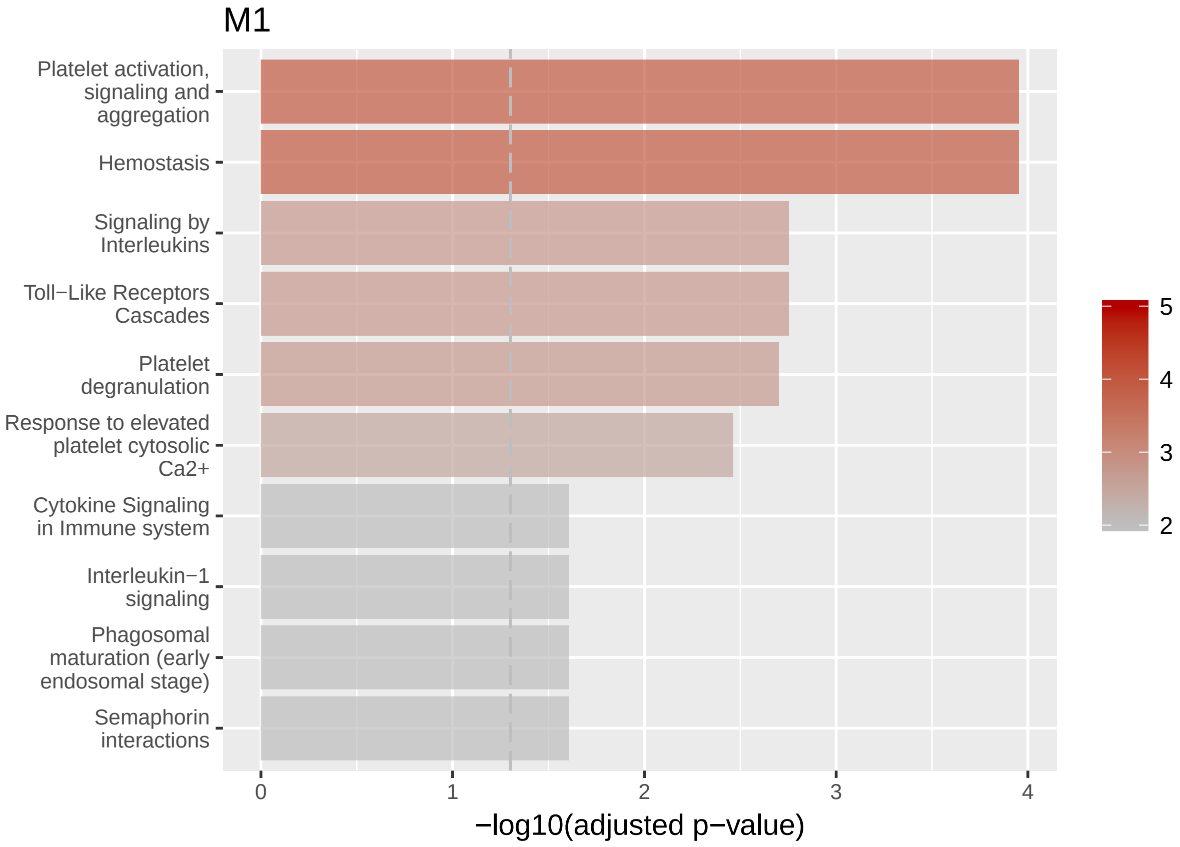

1.4.2 M2

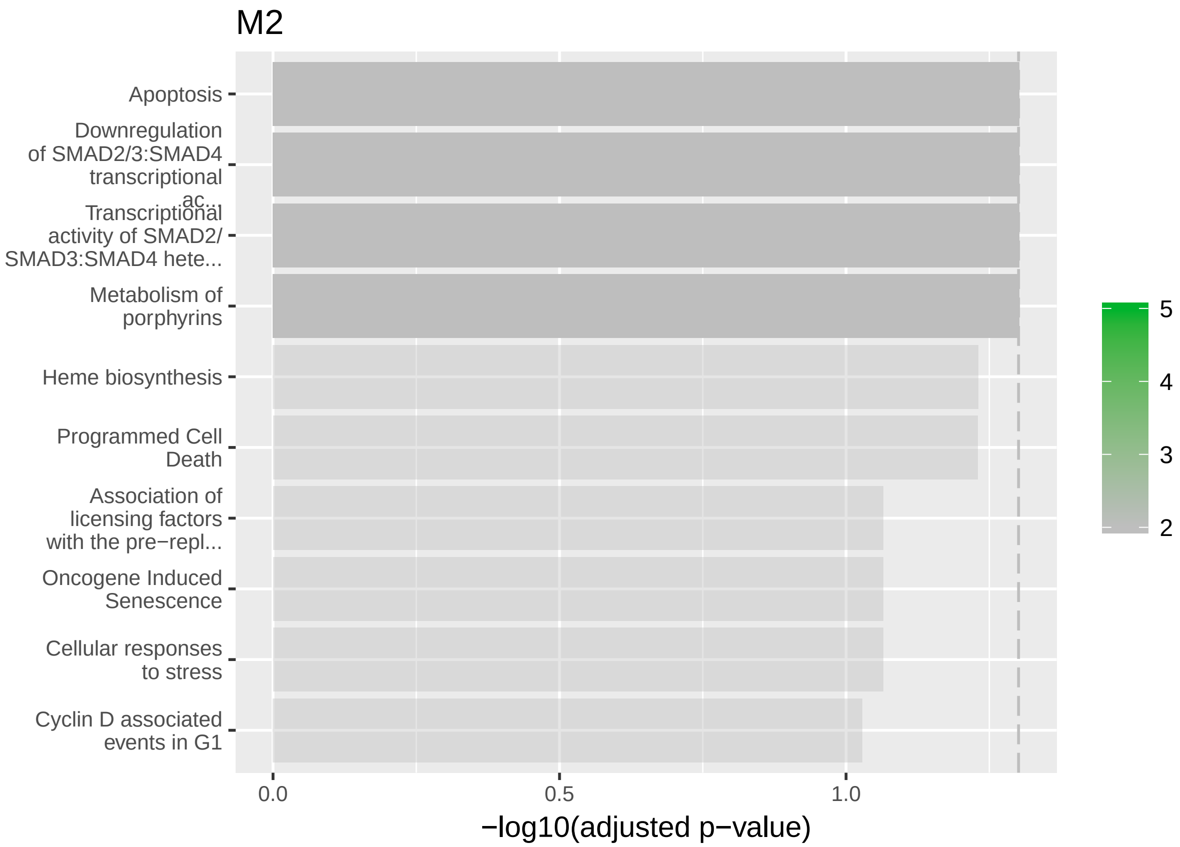
M2
Apoptosis
Downregulation of SMAD2/3:SMAD4 transcriptional ac...
Transcriptional activity of SMAD2/ SMAD3:SMAD4 hete...
Metabolism of porphyrins
Heme biosynthesis
Programmed Cell Death
Association of licensing factors with the pre−repl...
Oncogene Induced Senescence
Cellular responses to stress
Cyclin D associated events in G1
0.0
0.5
1.0
−log10(adjusted p−value)
5
4
3
2

1.4.3 M3

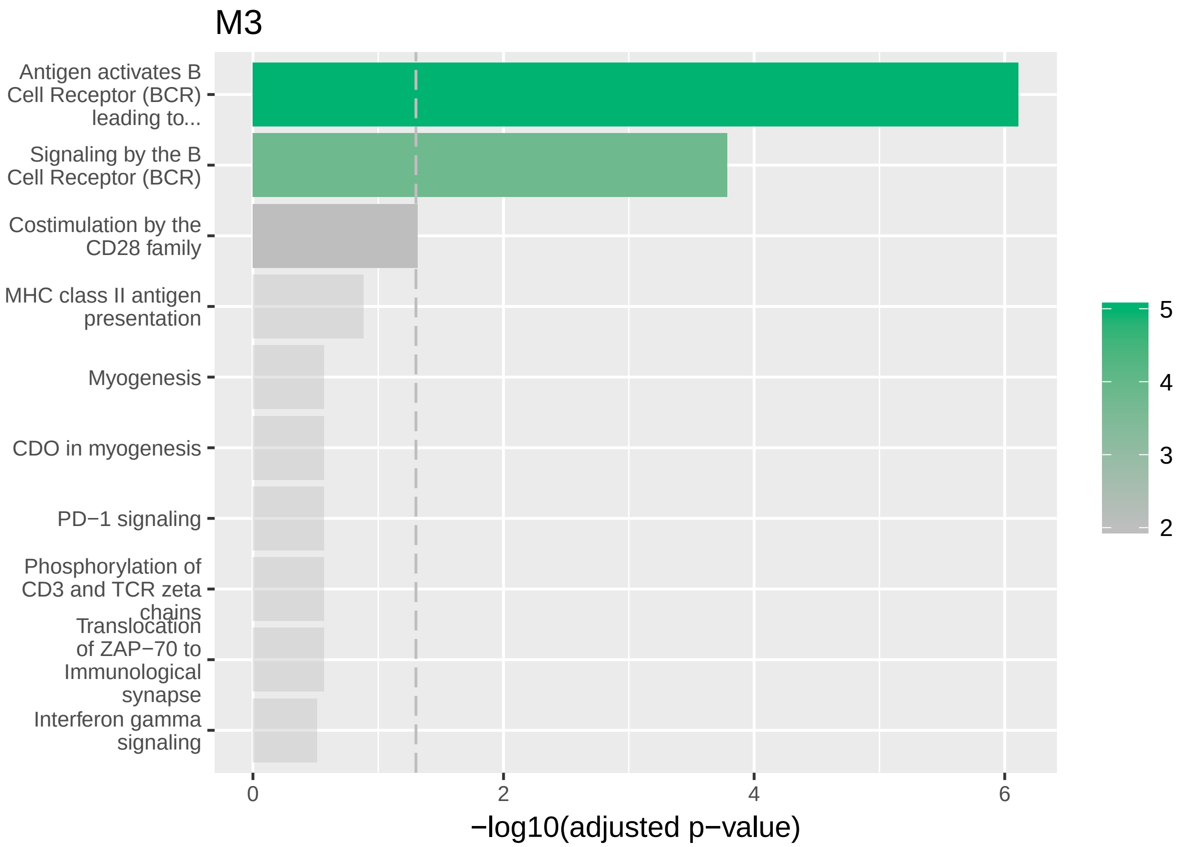

M3
Antigen activates B Cell Receptor (BCR) leading to...
Signaling by the B Cell Receptor (BCR)
Costimulation by the CD28 family
MHC class II antigen presentation
Myogenesis
CDO in myogenesis
PD−1 signaling
Phosphorylation of CD3 and TCR zeta chains
Translocation of ZAP−70 to Immunological synapse
Interferon gamma signaling
0
2
4
6
−log10(adjusted p−value)
5
4
3
2

1.4.4 M4

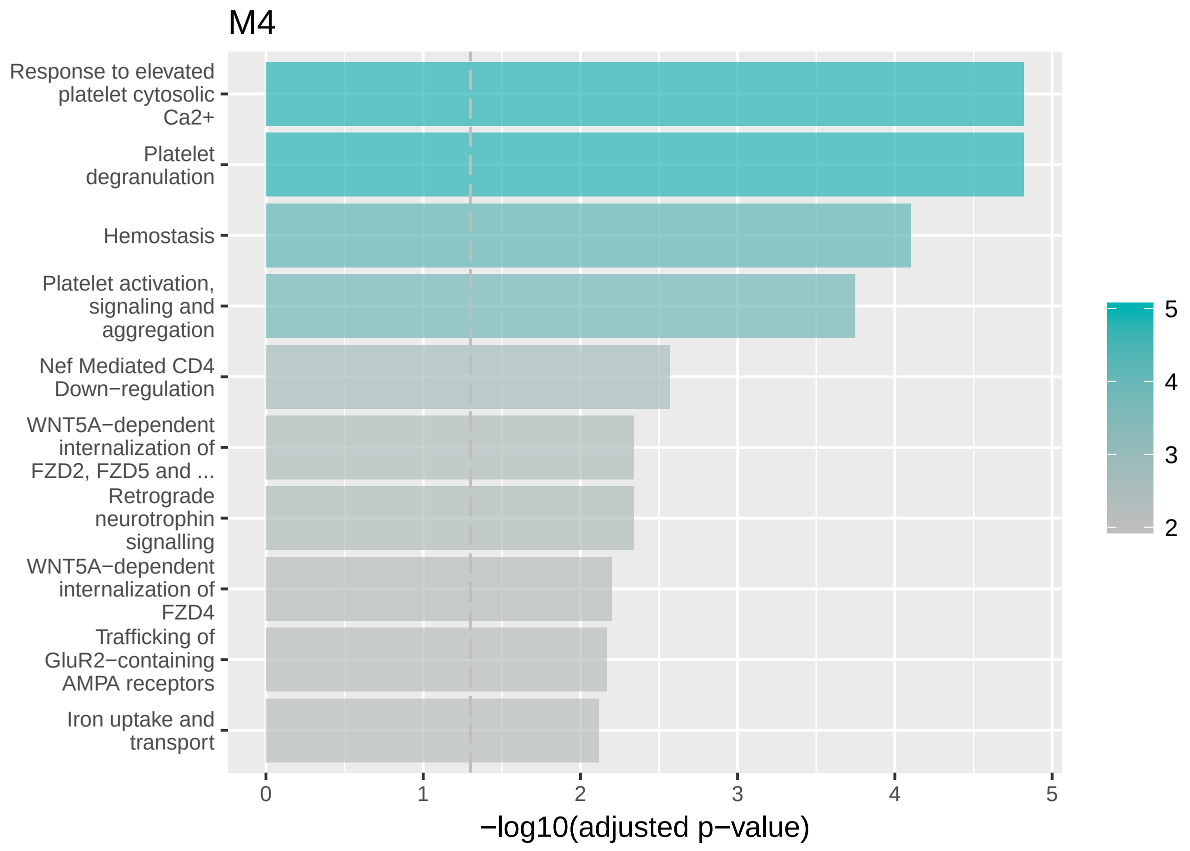

M4
Response to elevated platelet cytosolic Ca2+
Platelet degranulation
Hemostasis
Platelet activation, signaling and aggregation
Nef Mediated CD4 Down−regulation
WNT5A−dependent internalization of FZD2, FZD5 and ...
Retrograde neurotrophin signalling
WNT5A−dependent internalization of FZD4
Trafficking of GluR2−containing AMPA receptors
Iron uptake and transport
0
1
2
3
4
5
−log10(adjusted p−value)
5
4
3
2

1.4.5 M5

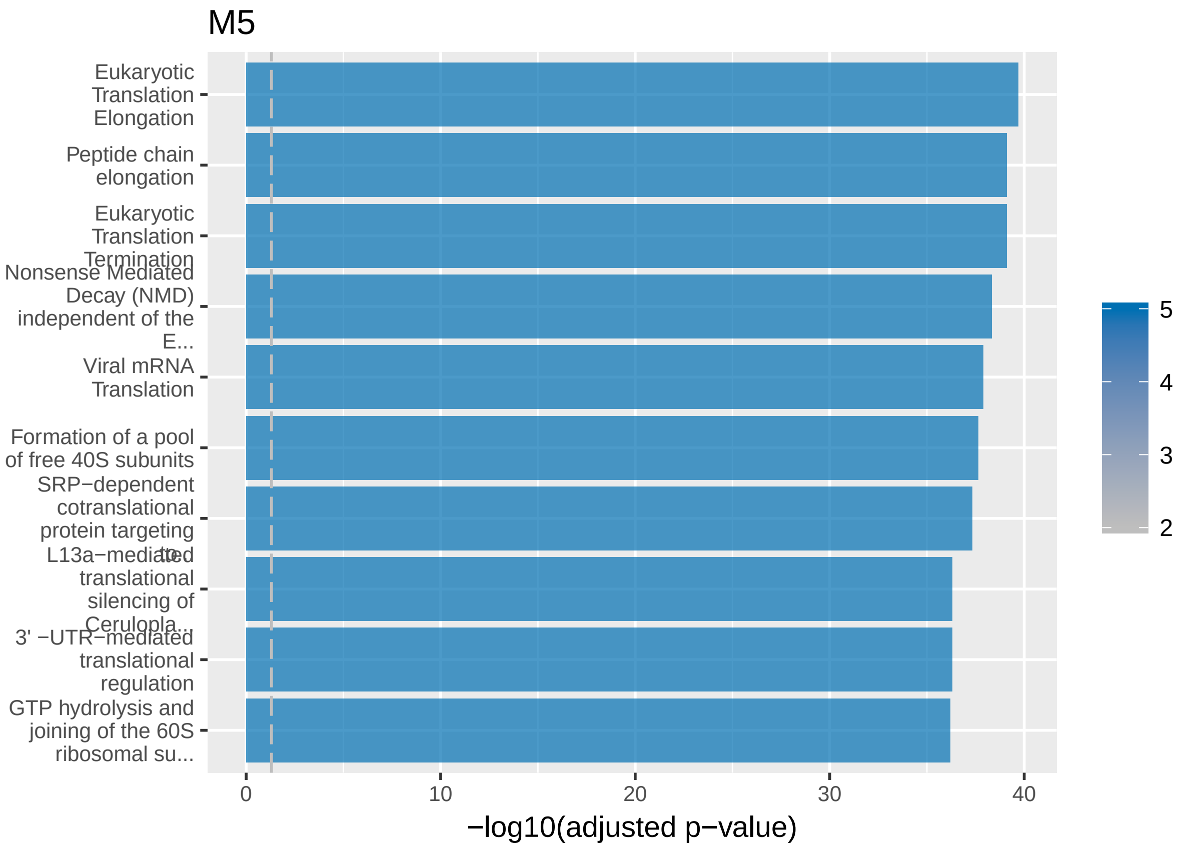

M5
Eukaryotic Translation Elongation
Peptide chain elongation
Eukaryotic Translation Termination
Nonsense Mediated Decay (NMD) independent of the E...
Viral mRNA Translation
Formation of a pool of free 40S subunits
SRP−dependent cotranslational protein targeting
L13a−mediated translational silencing of Cerulopla...
3' −UTR−mediated translational regulation
GTP hydrolysis and joining of the 60S ribosomal su...
0
10
20
30
40
−log10(adjusted p−value)
5
4
3
2

1.4.6 M6

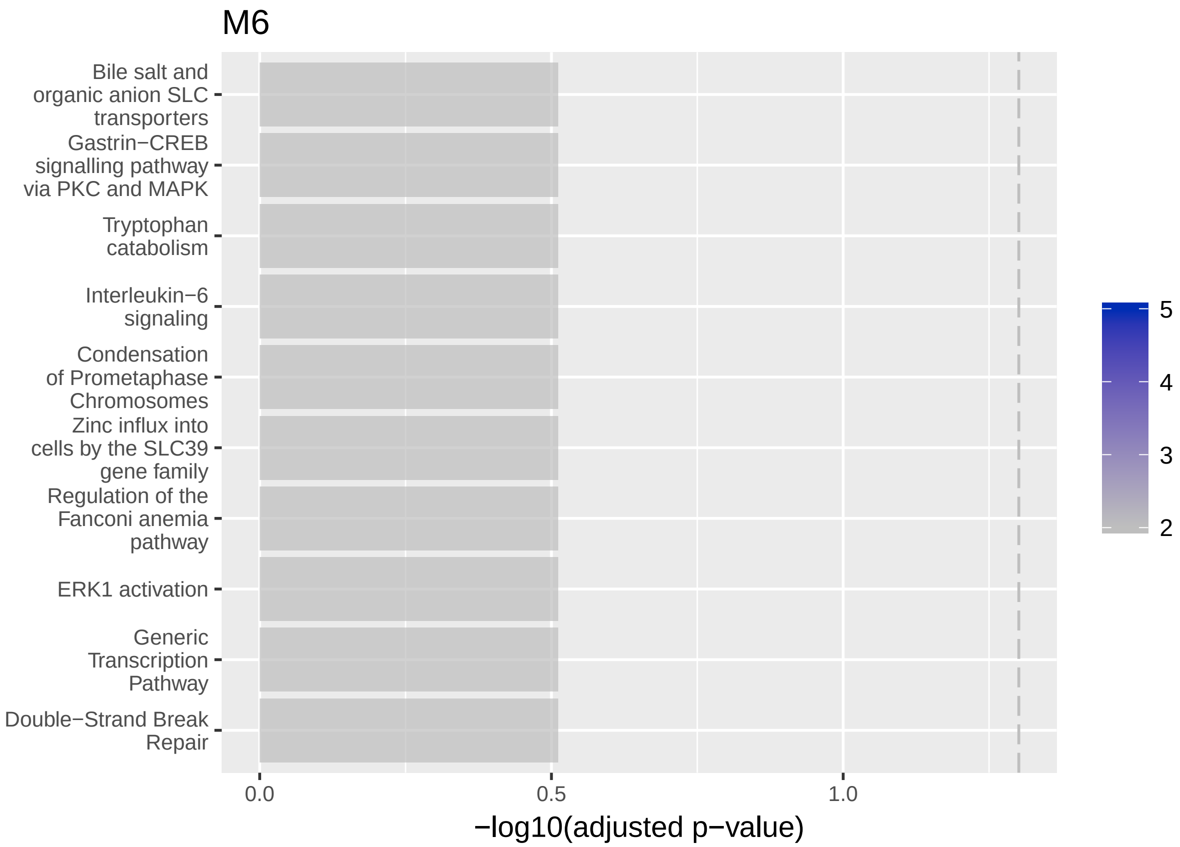
M6
Bile salt and organic anion SLC transporters
Gastrin−CREB signalling pathway via PKC and MAPK
Tryptophan catabolism
Interleukin−6 signaling
Condensation of Prometaphase Chromosomes
Zinc influx into cells by the SLC39 gene family
Regulation of the Fanconi anemia pathway
ERK1 activation
Generic Transcription Pathway
Double−Strand Break Repair
0.0
0.5
1.0
−log10(adjusted p−value)
5
4
3
2

1.4.7 M7

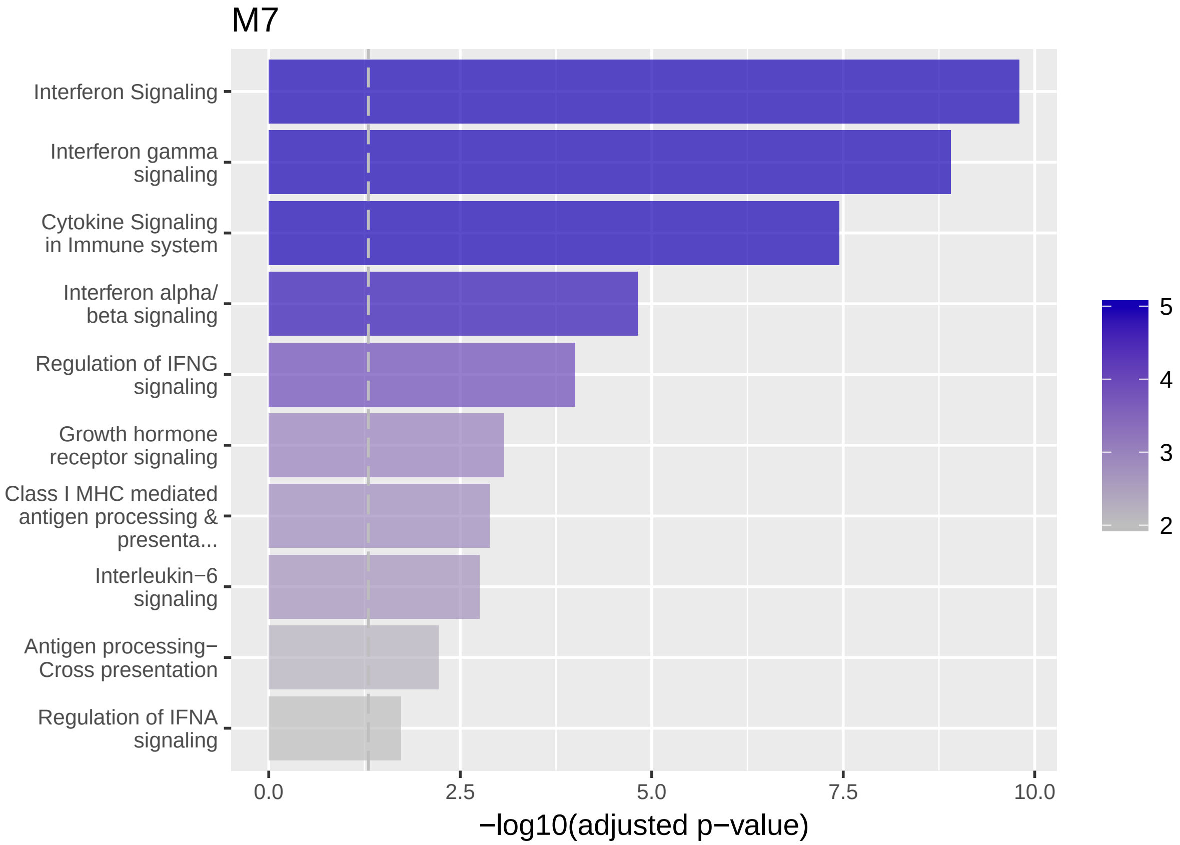

M7
Interferon Signaling
Interferon gamma signaling
Cytokine Signaling in Immune system
Interferon alpha/ beta signaling
Regulation of IFNG signaling
Growth hormone receptor signaling
Class I MHC mediated antigen processing & presenta...
Interleukin−6 signaling
Antigen processing− Cross presentation
Regulation of IFNA signaling
0.0
2.5
5.0
7.5
10.0
−log10(adjusted p−value)
5
4
3
2

1.4.8 M8

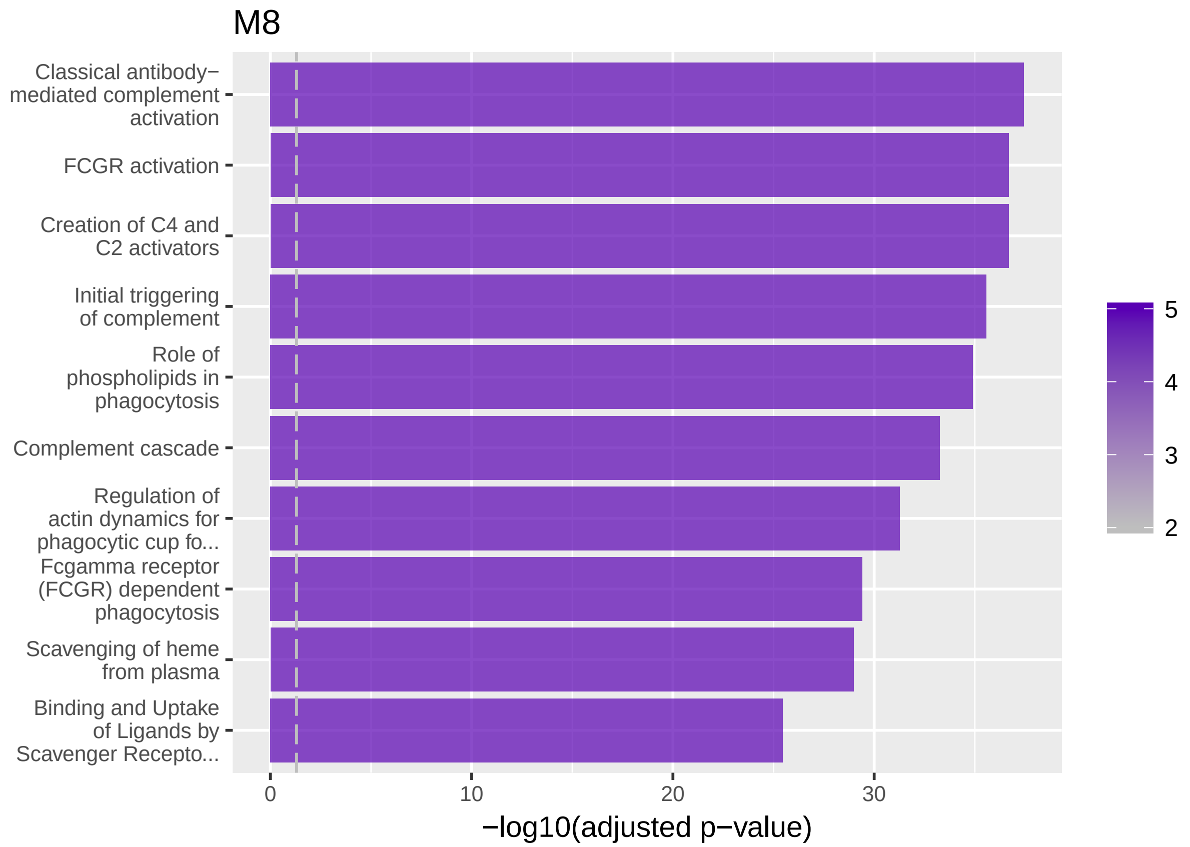

M8
Classical antibody−mediated complement activation
FCGR activation
Creation of C4 and C2 activators
Initial triggering of complement
Role of phospholipids in phagocytosis
Complement cascade
Regulation of actin dynamics for phagocytic cup fo...
Fcgamma receptor (FCGR) dependent phagocytosis
Scavenging of heme from plasma
Binding and Uptake of Ligands by Scavenger Recepto...
0
10
20
30
−log10(adjusted p−value)
5
4
3
2

1.4.9 M9

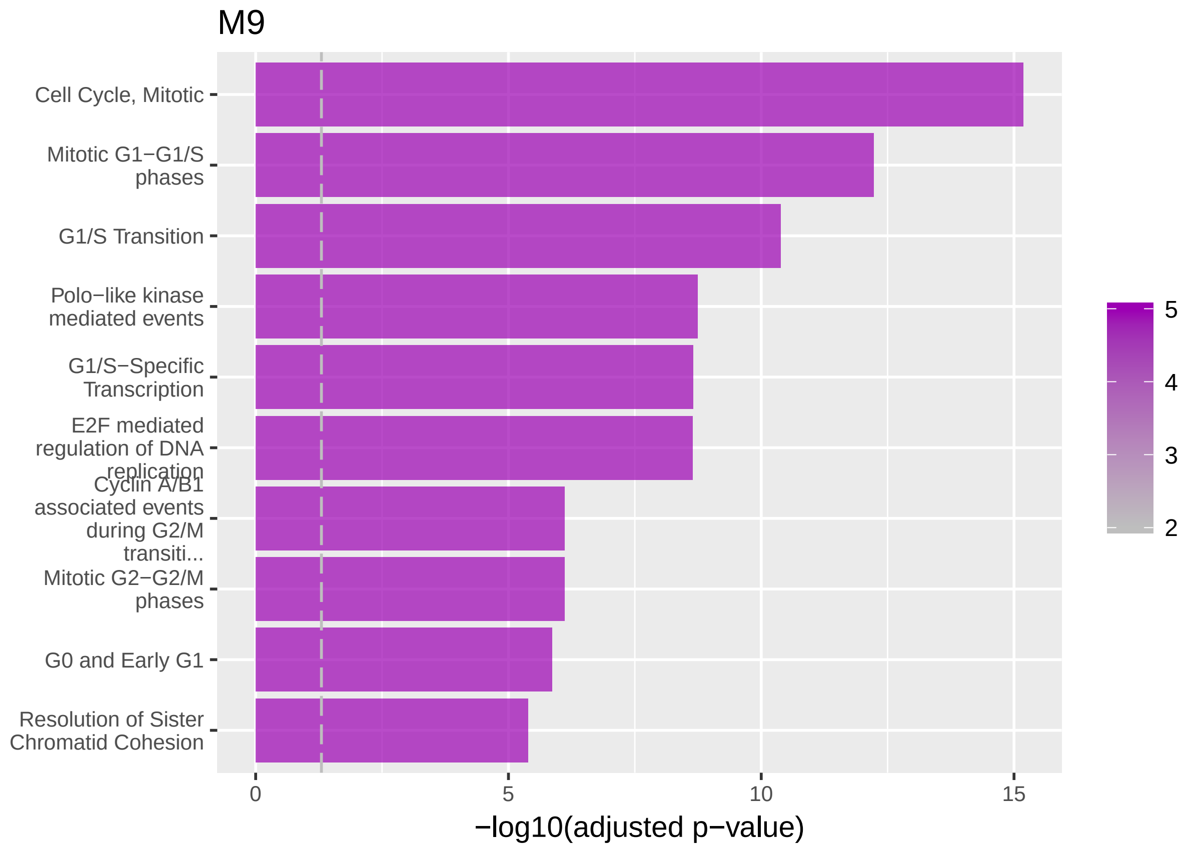

M9
Cell Cycle, Mitotic
Mitotic G1−G1/S phases
G1/S Transition
Polo−like kinase mediated events
G1/S−Specific Transcription
E2F mediated regulation of DNA replication
Cyclin A/B1 associated events during G2/M transiti...
Mitotic G2−G2/M phases
G0 and Early G1
Resolution of Sister Chromatid Cohesion
0
5
10
15
−log10(adjusted p−value)
5
4
3
2

1.4.10 M10

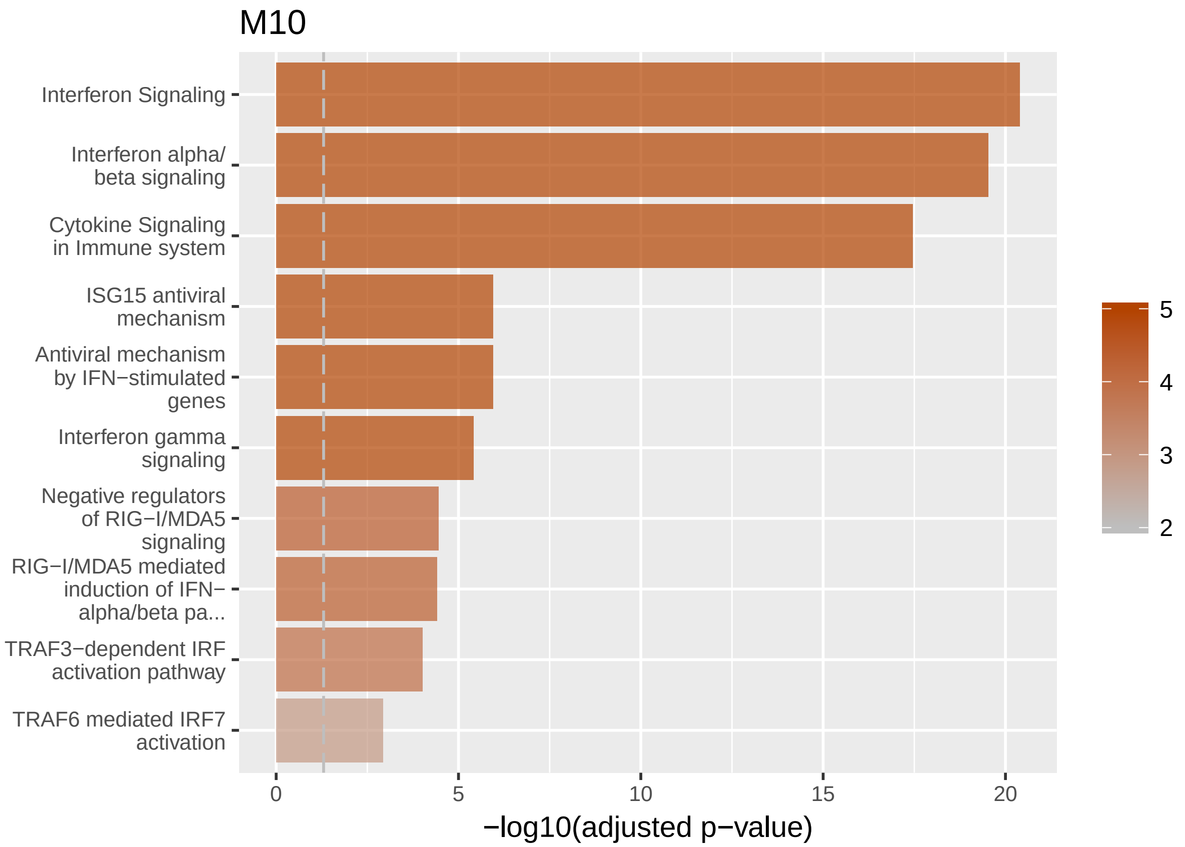
M10
Interferon Signaling
Interferon alpha/ beta signaling
Cytokine Signaling in Immune system
ISG15 antiviral mechanism
Antiviral mechanism by IFN−stimulated genes
Interferon gamma signaling
Negative regulators of RIG−I/MDA5 signaling
RIG−I/MDA5 mediated induction of IFN−alpha/beta pa...
TRAF3−dependent IRF activation pathway
TRAF6 mediated IRF7 activation
0
5
10
15
20
−log10(adjusted p−value)
5
4
3
2

1.4.11 M11

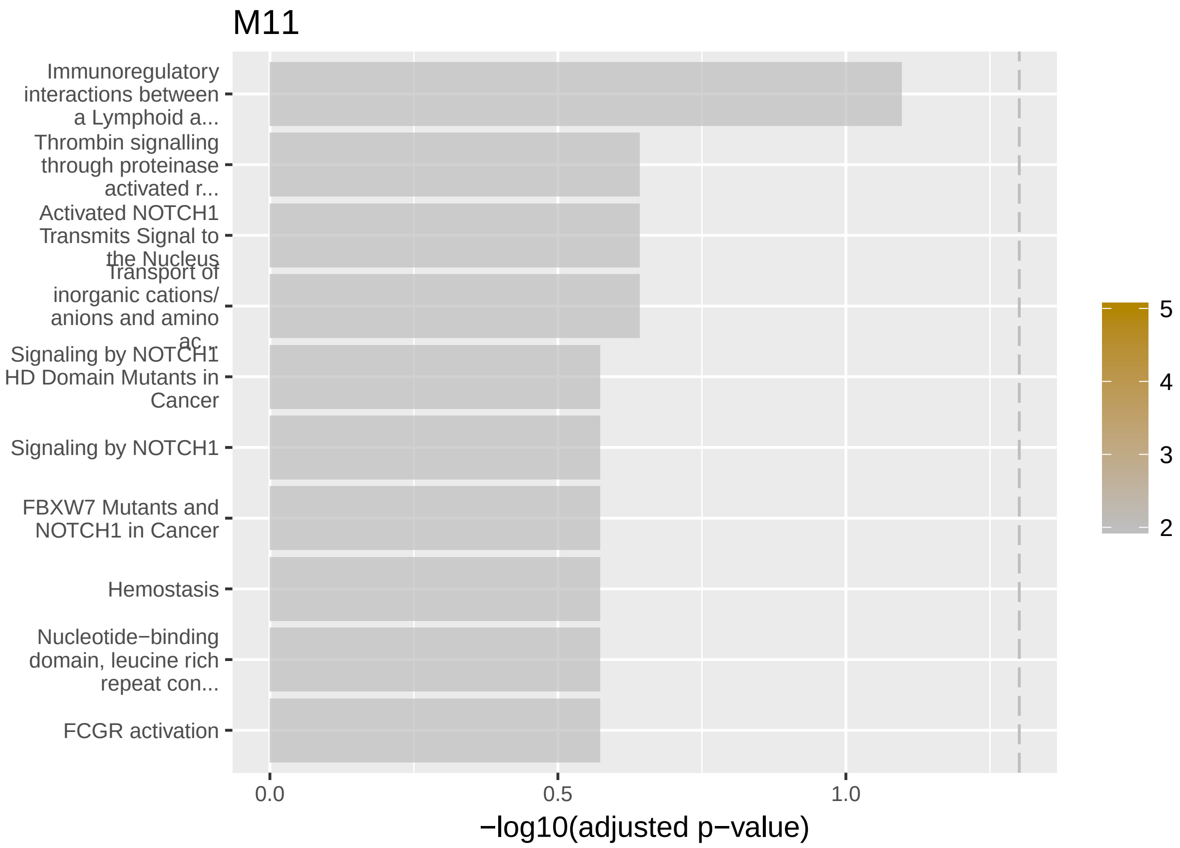

M11
Immunoregulatory interactions between a Lymphoid a...
Thrombin signalling through proteinase activated r...
Activated NOTCH1 Transmits Signal to the Nucleus
Transport of inorganic cations/ anions and amino ac...
Signaling by NOTCH1 HD Domain Mutants in Cancer
Signaling by NOTCH1
FBXW7 Mutants and NOTCH1 in Cancer
Hemostasis
Nucleotide−binding domain, leucine rich repeat con...
FCGR activation
0.0
0.5
1.0
−log10(adjusted p−value)
5
4
3
2

1.4.12 M12

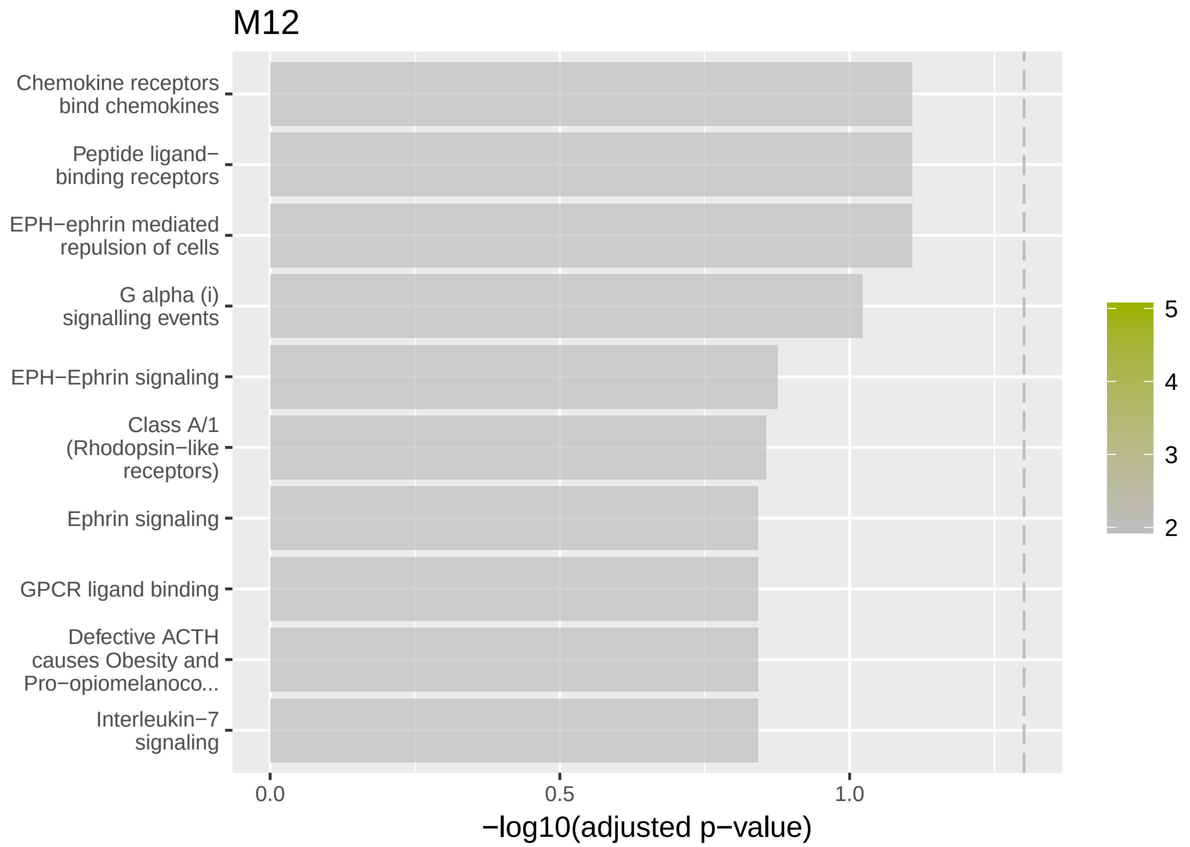
M12
Chemokine receptors bind chemokines
Peptide ligand−binding receptors
EPH−ephrin mediated repulsion of cells
G alpha (i) signalling events
EPH−Ephrin signaling
Class A/1 (Rhodopsin−like receptors)
Ephrin signaling
GPCR ligand binding
Defective ACTH causes Obesity and Pro−opiomelanoco...
Interleukin−7 signaling
0.0
0.5
1.0
−log10(adjusted p−value)
5
4
3
2

1.4.13 M13

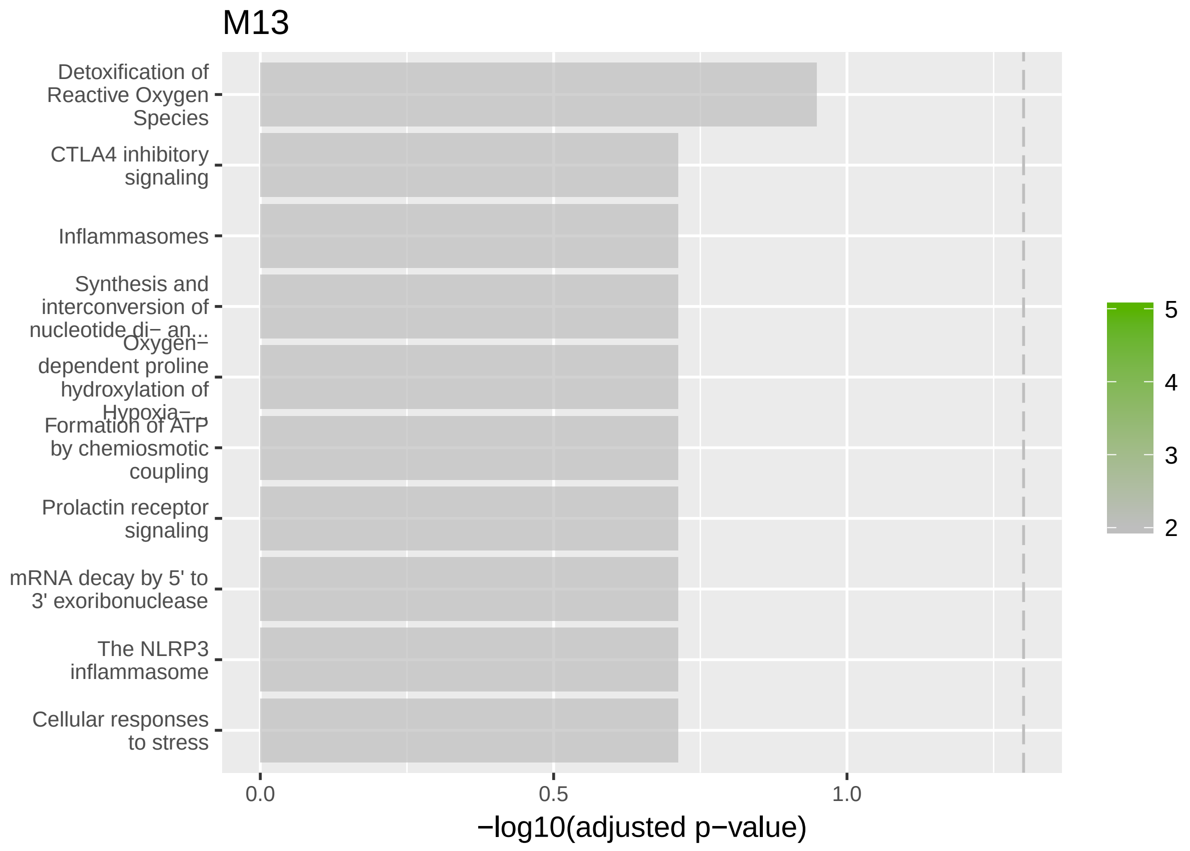
M13
Detoxification of Reactive Oxygen Species
CTLA4 inhibitory signaling
Inflammasomes
Synthesis and interconversion of nucleotide di- an...
Oxygen-dependent proline hydroxylation of Hypoxia-
Formation of ATP by chemiosmotic coupling
Prolactin receptor signaling
mRNA decay by 5' to 3' exoribonuclease
The NLRP3 inflammasome
Cellular responses to stress
0.0
0.5
1.0
−log10(adjusted p−value)
5
4
3
2

1.4.14 M14

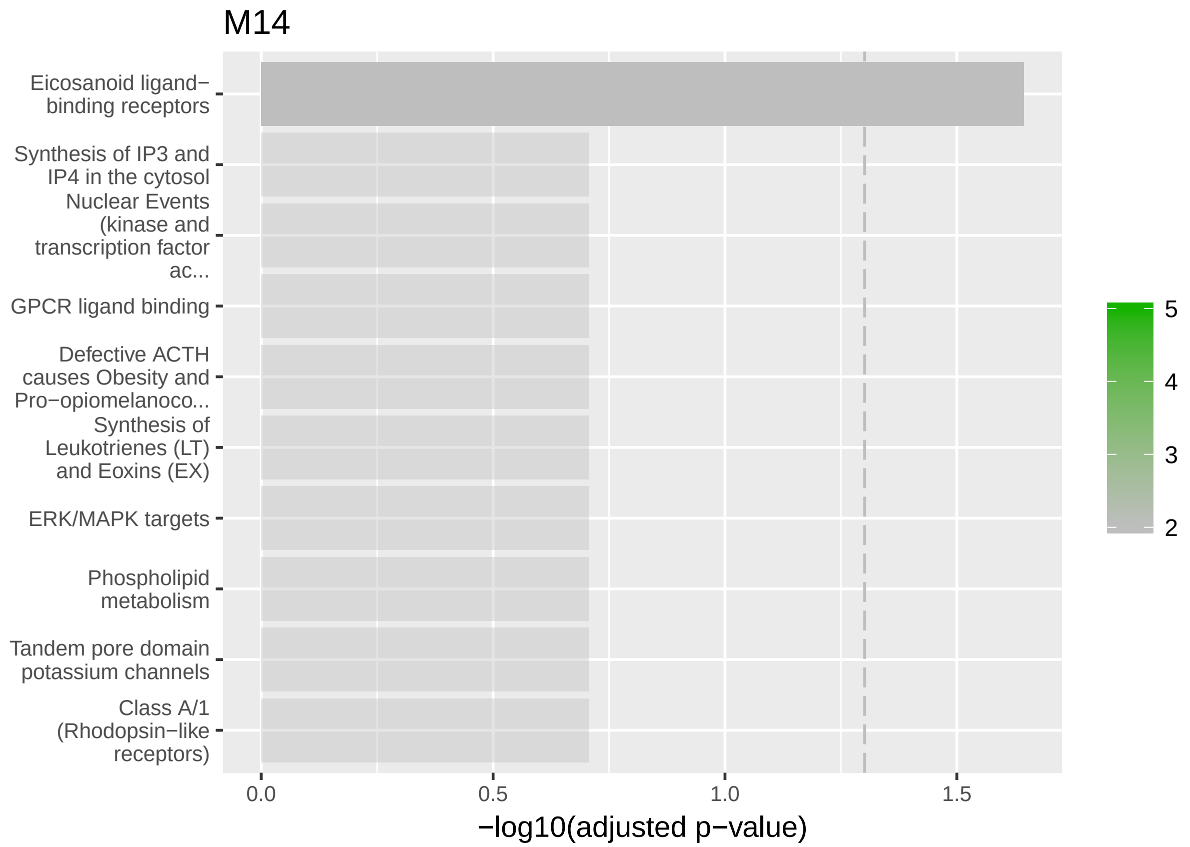

M14
Eicosanoid ligand−binding receptors
Synthesis of IP3 and IP4 in the cytosol
Nuclear Events (kinase and transcription factor ac...
GPCR ligand binding
Defective ACTH causes Obesity and Pro−opiomelanoco...
Synthesis of Leukotrienes (LT) and Eoxins (EX)
ERK/MAPK targets
Phospholipid metabolism
Tandem pore domain potassium channels
Class A/1 (Rhodopsin−like receptors)
0.0
0.5
1.0
1.5
−log10(adjusted p−value)
5
4
3
2

1.5 Interaction Network

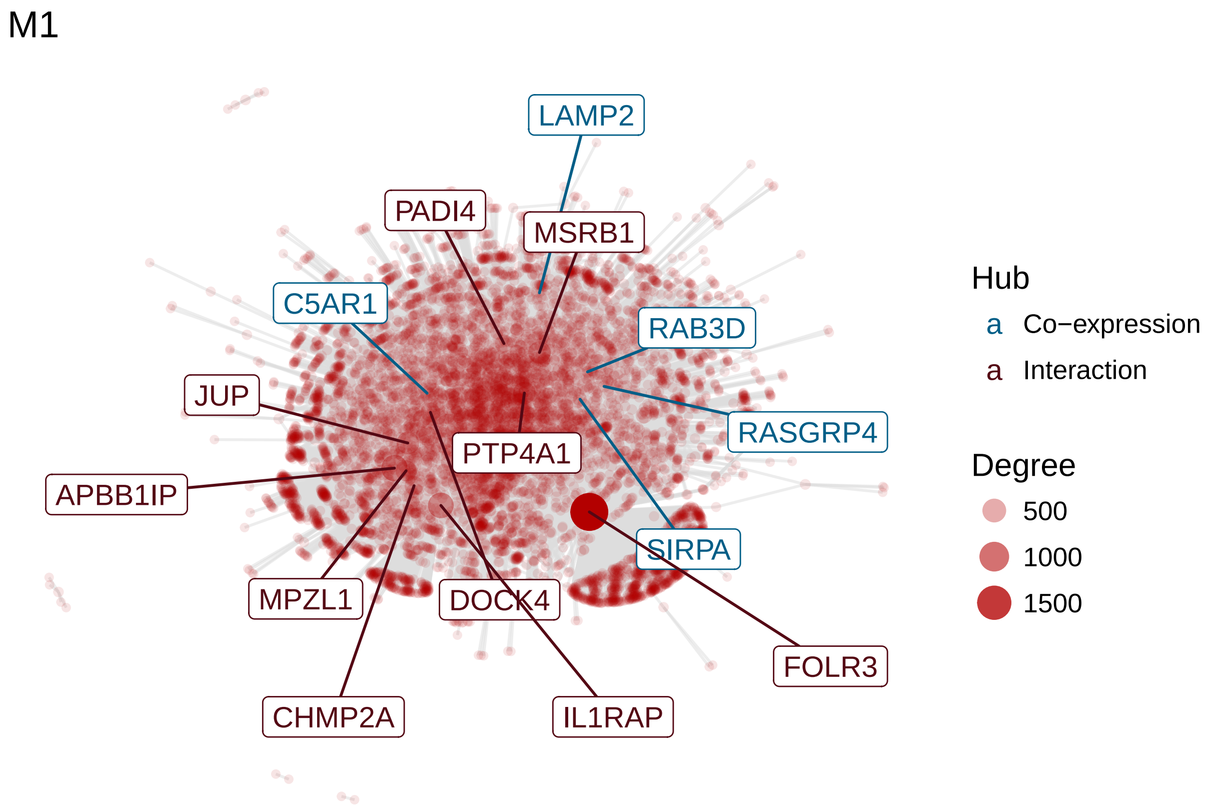

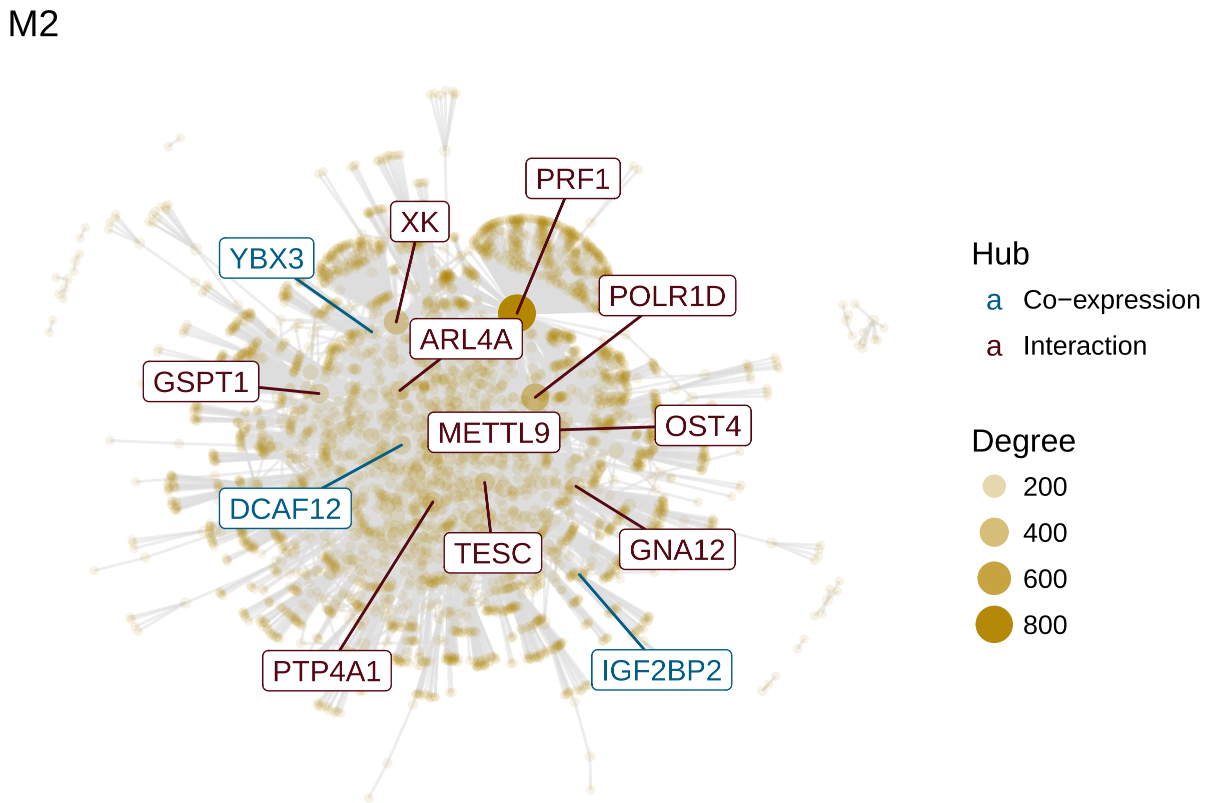

M3

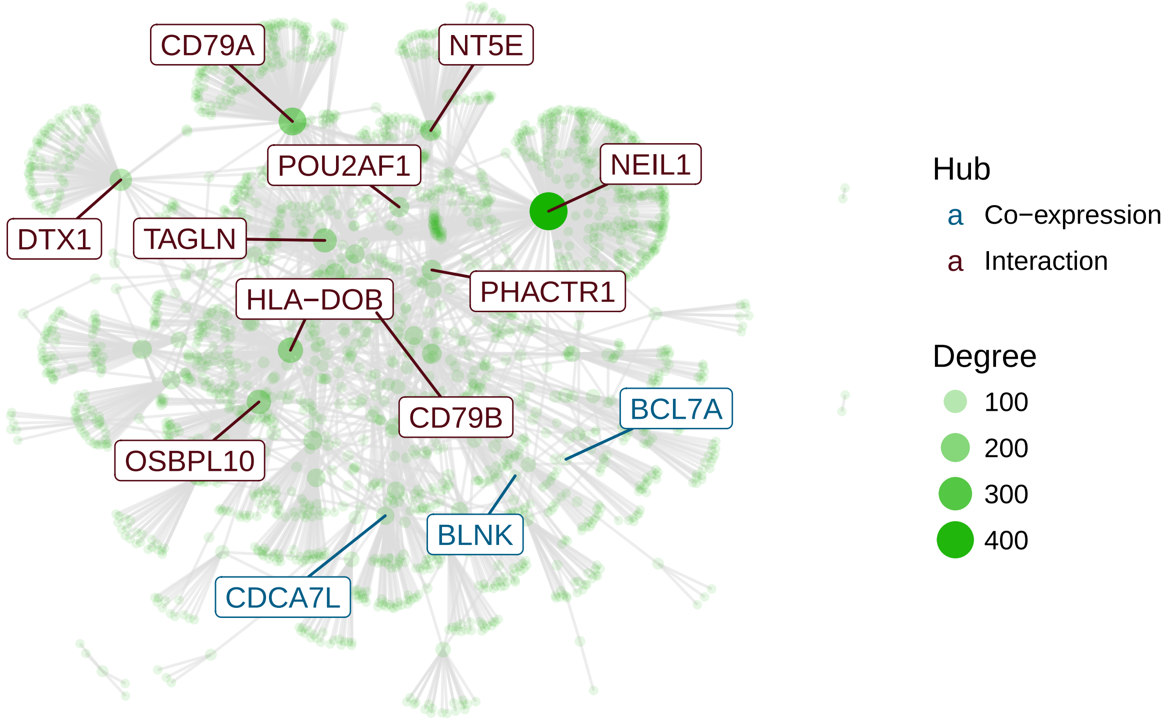

CD79A
NT5E
POU2AF1
NEIL1
DTX1
TAGLN
PHACTR1
HLA−DOB
CD79B
BCL7A
OSBPL10
BLNK
CDCA7L
Hub
a Co−expression
a Interaction
Degree
100
200
300
400

M4

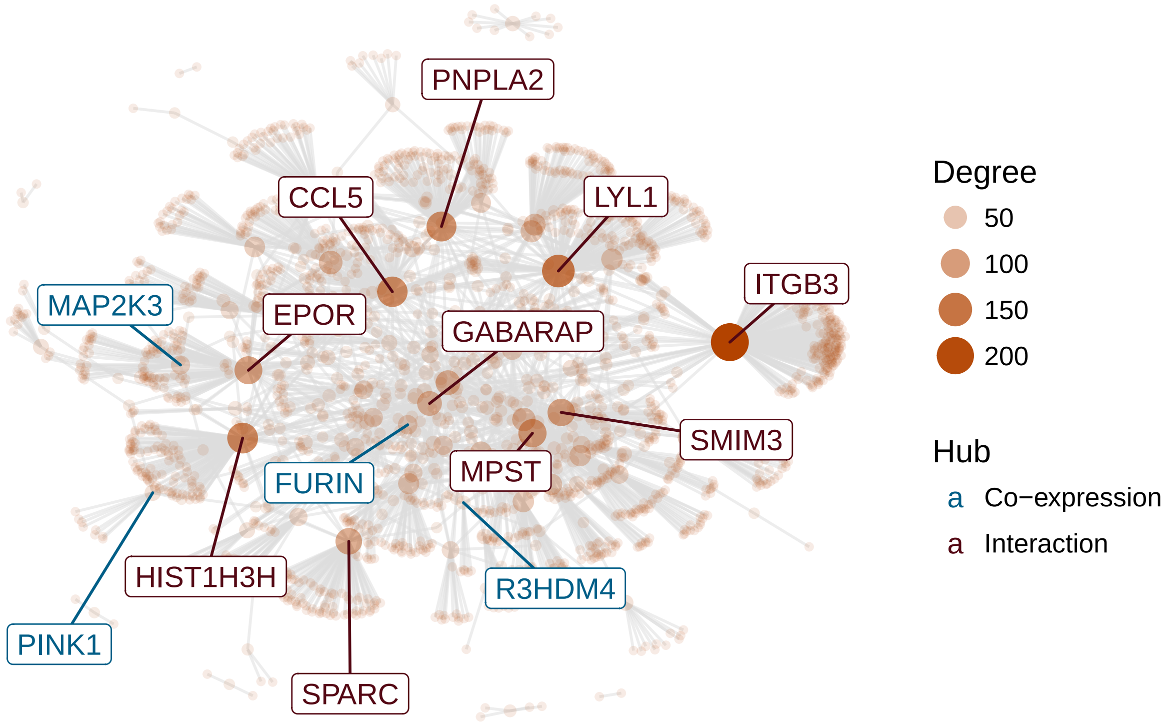

PNPLA2
CCL5
LYL1
MAP2K3
EPOR
ITGB3
GABARAP
SMIM3
FURIN
MPST
HIST1H3H
R3HDM4
PINK1
SPARC
Degree
50
100
150
200
Hub
a Co−expression
a Interaction

M5

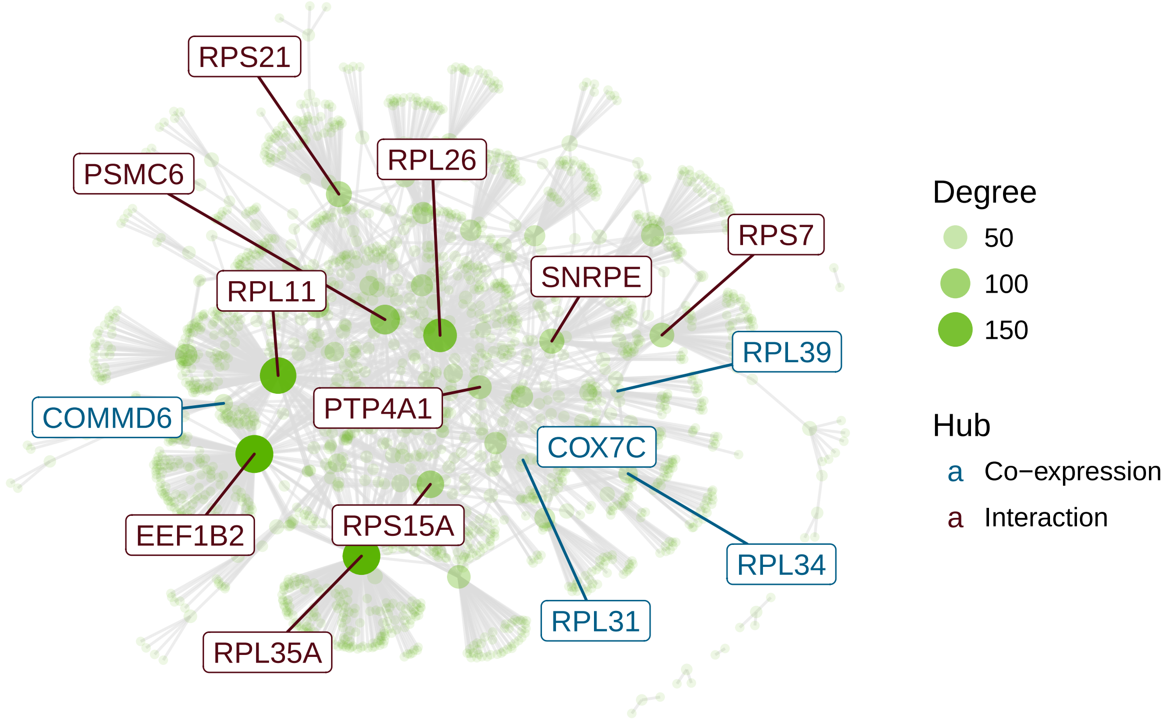
RPS21
PSMC6
RPL26
RPS7
SNRPE
RPL11
RPL39
COMMD6
PTP4A1
COX7C
EEF1B2
RPS15A
RPL34
RPL31
RPL35A
Degree
50
100
150
Hub
a Co−expression
a Interaction

M6

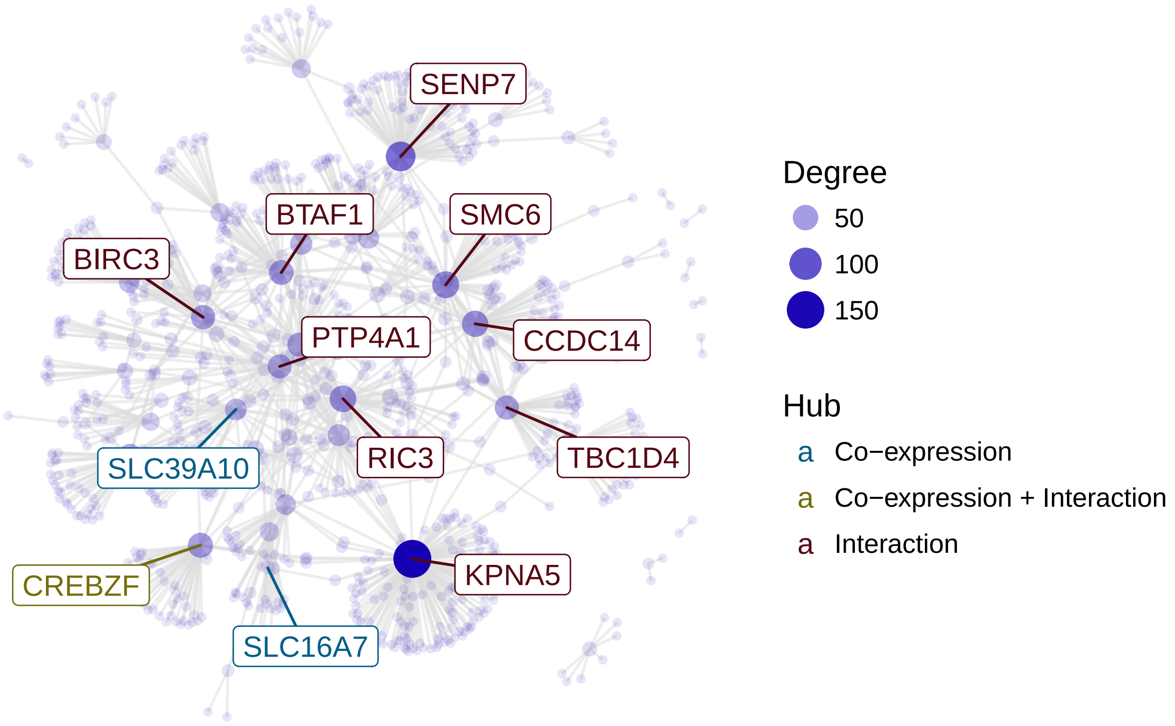
SENP7
BTAF1
SMC6
BIRC3
PTP4A1
CCDC14
SLC39A10
RIC3
TBC1D4
CREBZF
KPNA5
SLC16A7
Degree
50
100
150
Hub
a Co−expression
a Co−expression + Interaction
a Interaction

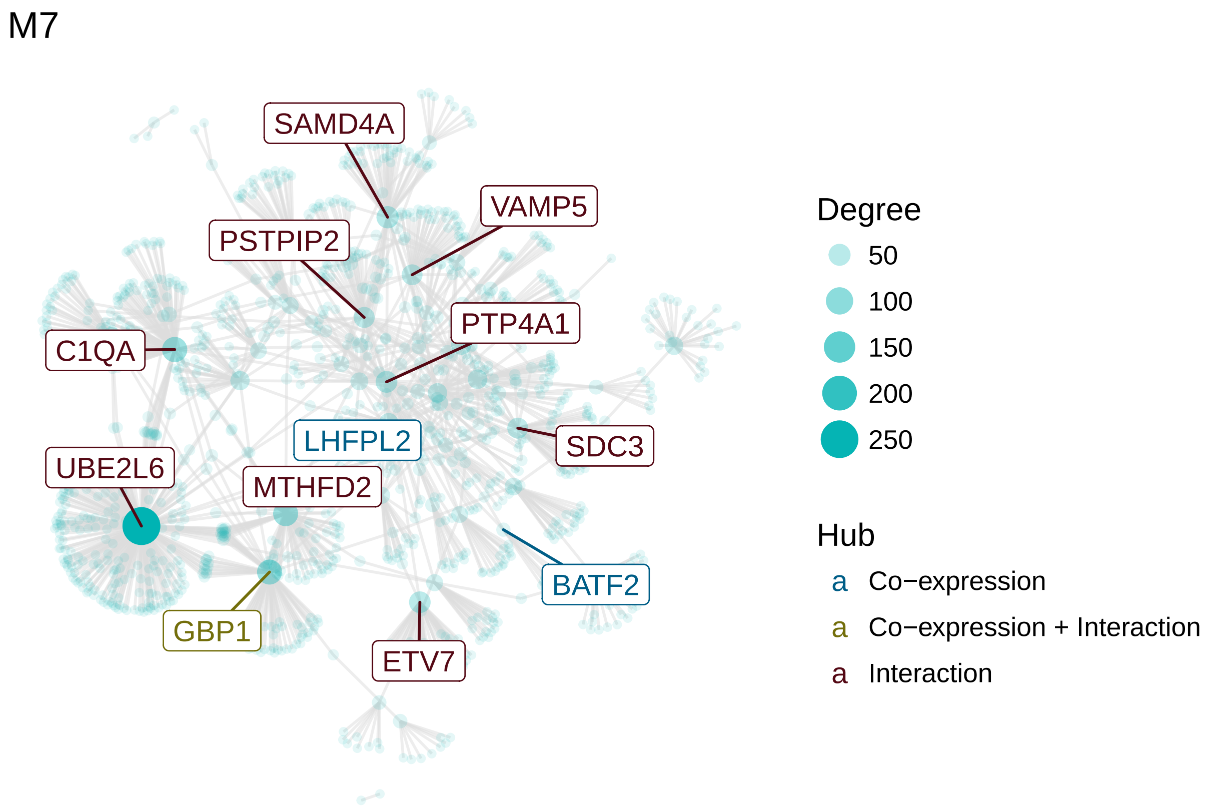
M7
SAMD4A
VAMP5
PSTPIP2
C1QA
PTP4A1
LHFPL2
SDC3
UBE2L6
MTHFD2
BATF2
GBP1
ETV7
Degree
50
100
150
200
250
Hub
a Co−expression
a Co−expression + Interaction
a Interaction

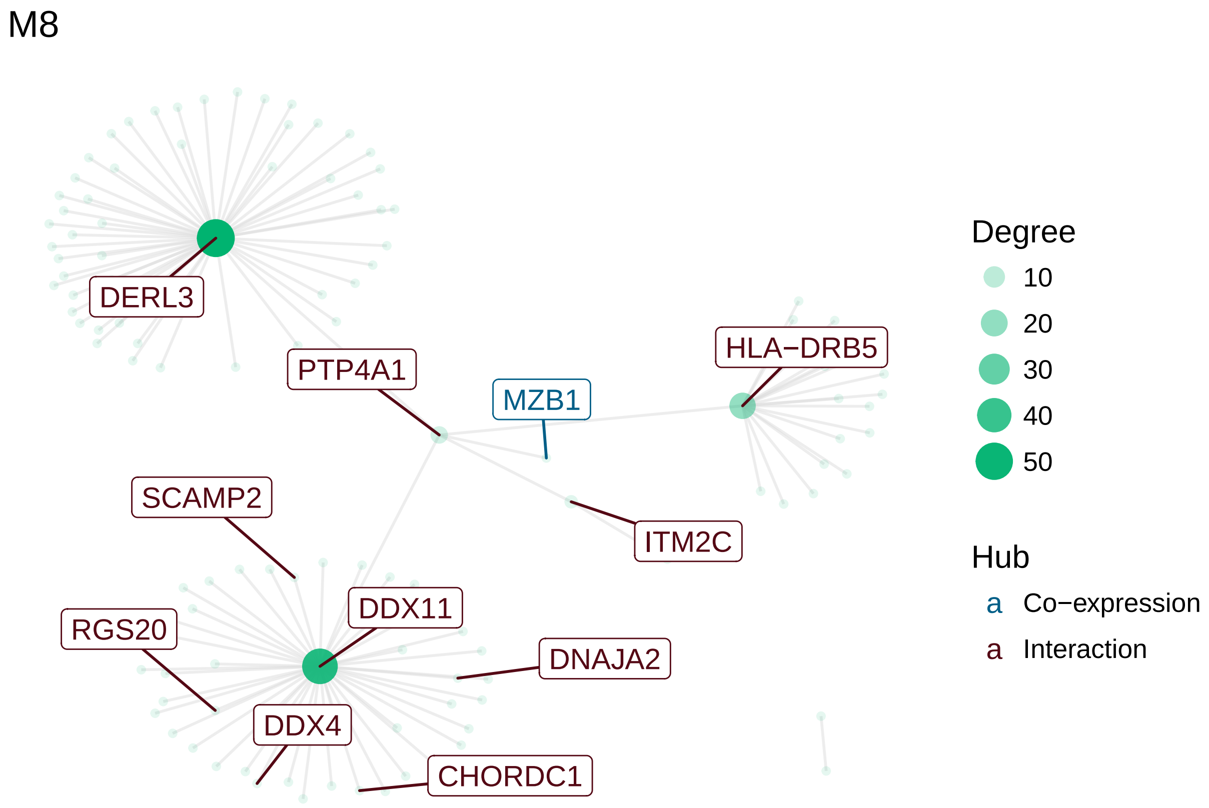
M8
DERL3
PTP4A1
MZB1
HLA−DRB5
SCAMP2
ITM2C
DDX11
RGS20
DNAJA2
DDX4
CHORDC1
Degree
10
20
30
40
50
Hub
a Co−expression
a Interaction

M9

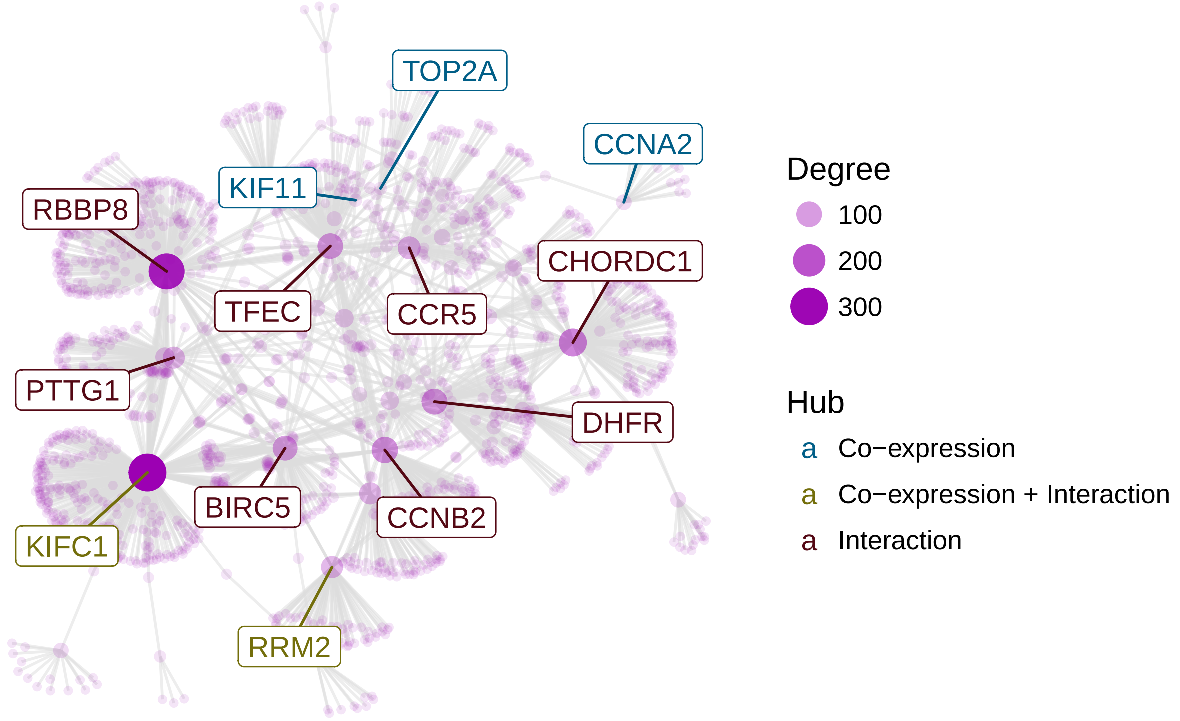
TOP2A
CCNA2
KIF11
RBBP8
CHORDC1
TFEC
CCR5
PTTG1
DHFR
BIRC5
CCNB2
KIFC1
RRM2
Degree
100
200
300
Hub
a Co−expression
a Co−expression + Interaction
a Interaction

M10

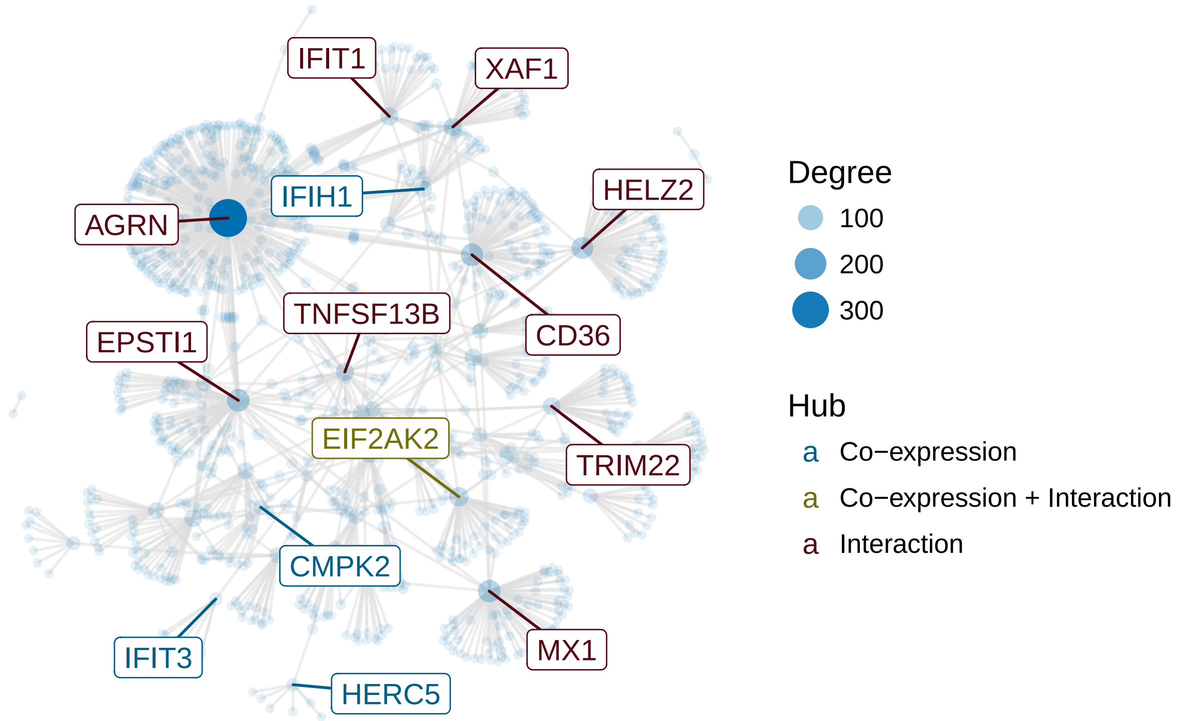
IFIT1
XAF1
HELZ2
IFIH1
AGRN
TNFSF13B
EPSTI1
CD36
EIF2AK2
TRIM22
CMPK2
MX1
IFIT3
HERC5
Degree
100
200
300
Hub
a Co−expression
a Co−expression + Interaction
a Interaction

M11

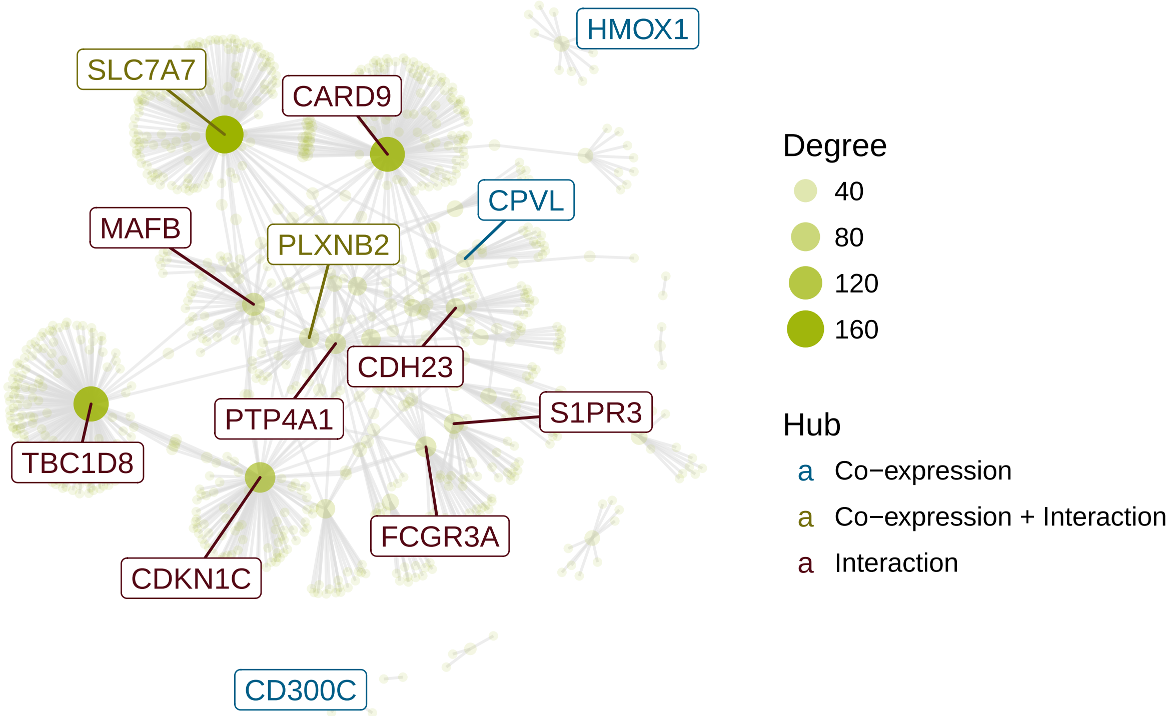

HMOX1
SLC7A7
CARD9
CPVL
MAFB
PLXNB2
CDH23
PTP4A1
S1PR3
TBC1D8
FCGR3A
CDKN1C
CD300C
Degree
40
80
120
160
Hub
a Co−expression
a Co−expression + Interaction
a Interaction

M12

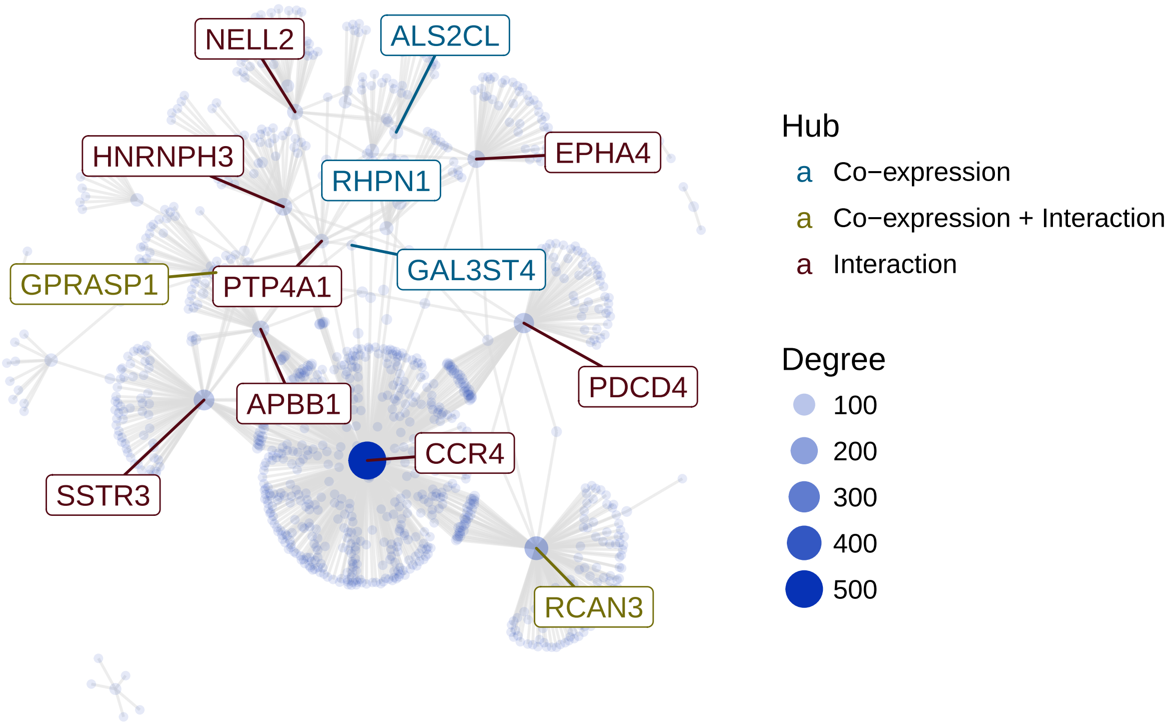

NELL2
ALS2CL
HNRNPH3
EPHA4
RHPN1
GPRASP1
PTP4A1
GAL3ST4
APBB1
PDCD4
CCR4
SSTR3
RCAN3
Hub
a Co−expression
a Co−expression + Interaction
a Interaction
Degree
100
200
300
400
500

M13

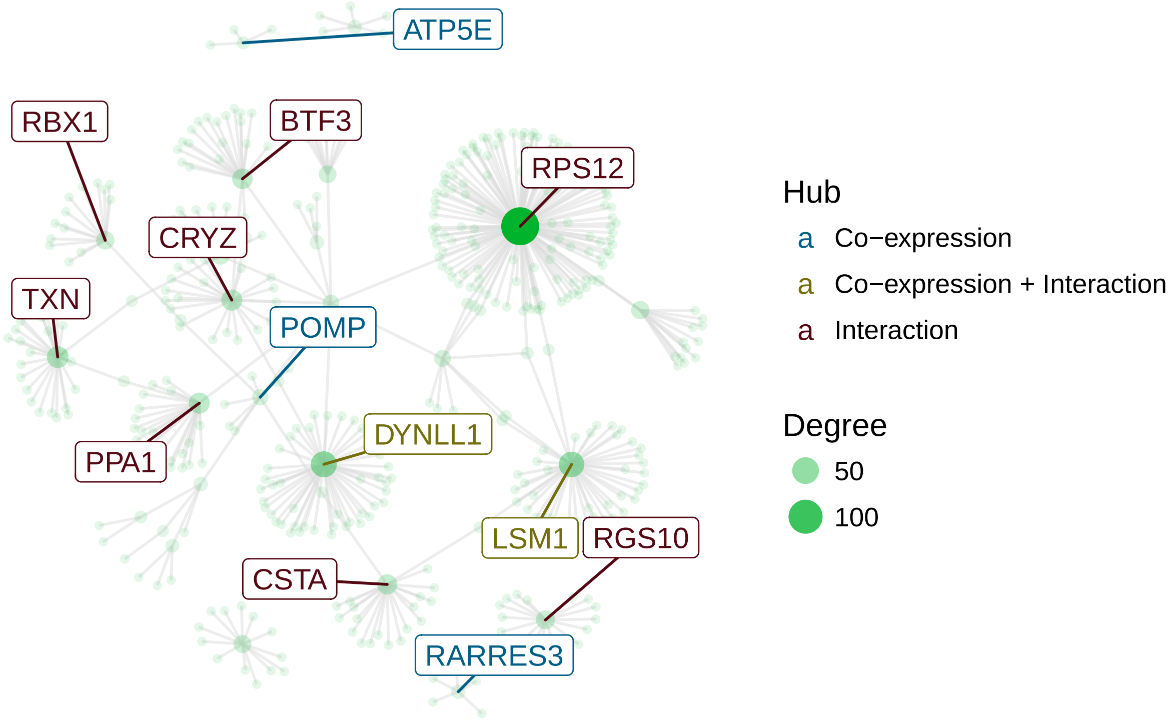

M14

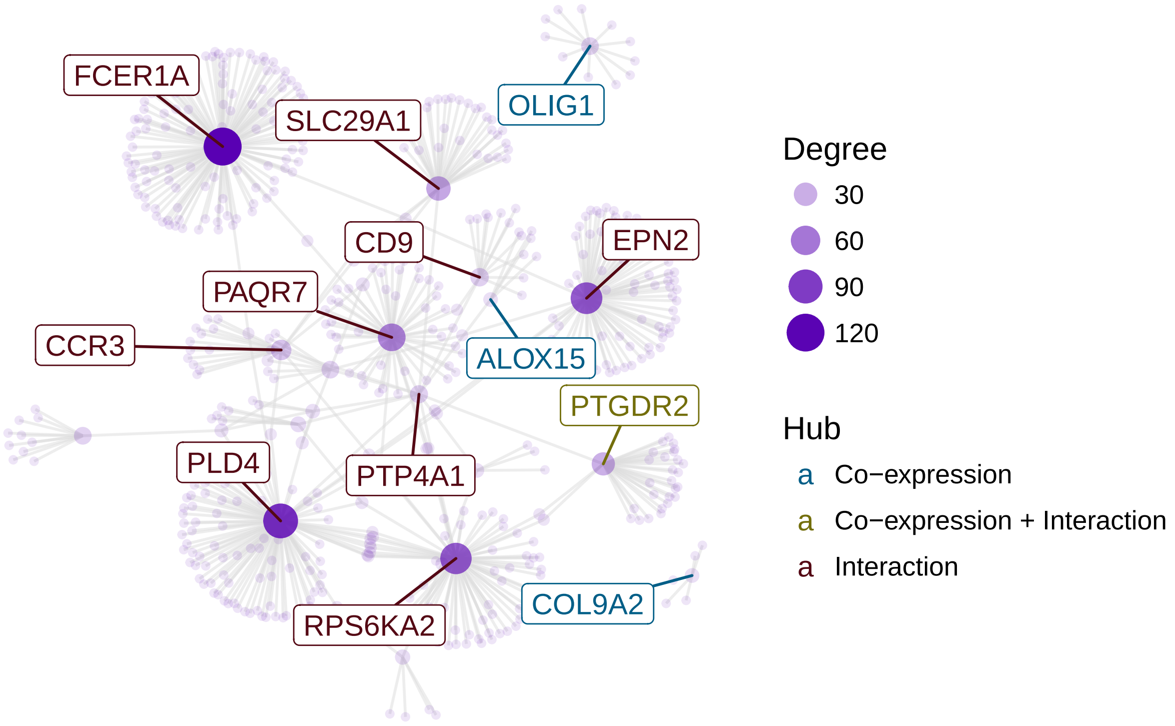

1.6 Parameters
